# Barbituric Acid Derivatives as Covalent Inhibitors of *Leishmania braziliensis* Dihydroorotate Dehydrogenase

**DOI:** 10.1021/acs.jmedchem.5c00462

**Published:** 2025-07-14

**Authors:** Thamires Quadros Froes, Temitayo Omowumi Alegbejo Price, Bruna Fleck Godoi, Miguel Menezes Vaidergorn, Thiago dos Santos, Pedro Ivo Palacio Leite, Daniel Gedder Silva, Aline Dias da Purificação, Leonardo Loch, Sergio Schenkman, Jadel M. Kratz, Flavio da Silva Emery, Maria Cristina Nonato

**Affiliations:** † Center for the Research and Advancement in Fragments and molecular Targets (CRAFT), School of Pharmaceutical Sciences at Ribeirao Preto, University of São Paulo, Ribeirão Preto, SP 14040-903, Brazil; ‡ Protein Crystallography Laboratory, Department of Biomolecular Sciences, School of Pharmaceutical Sciences of Ribeirao Preto, University of São Paulo, Ribeirão Preto, SP 14040-903, Brazil; § Departamento de Microbiologia Imunobiologia e Parasitologia, Disciplina de Biologia Celular, Universidade Federal de São Paulo, R. Pedro de Toledo 669 6A, Vila Clementino, São Paulo, SP 04039032, Brasil; & Drugs for Neglected Diseases initiative (DNDi) Latin America, Rio de Janeiro, SP 20010-020, Brazil

## Abstract

Covalent drug design applied to parasite proteins enables selective therapies by targeting nucleophilic residues of macromolecules. We present the first covalent inhibitors of *Leishmania braziliensis* dihydroorotate dehydrogenase (*Lb*DHODH), a key enzyme in pyrimidine biosynthesis with a reactive cysteine (Cys^131^) in its active site. From barbituric acid derivatives, we discovered **2i** as a *Lb*DHODH inhibitor with leishmanicidal activity, exhibiting an IC_5__0_ of 0.5 ± 0.1 μM, a K_inact_/K*I* of 767 M^–1^s^–1^, no inhibition of the human ortholog, and an EC_5__0_ of 11 ± 5 μM in *L. braziliensis* promastigotes, with no cytotoxicity in THP-1 cells and good passive permeability. X-ray crystallography confirms covalent bond formation with Cys^131^ and reveals active-site rearrangements. These findings support the proposed covalent inhibition mechanism and provide structural insights for further optimization. Our study validates *Lb*DHODH as a promising target for leishmaniasis therapy and highlights the potential of covalent inhibition in antiparasitic drug discovery.

## Graphical abstract

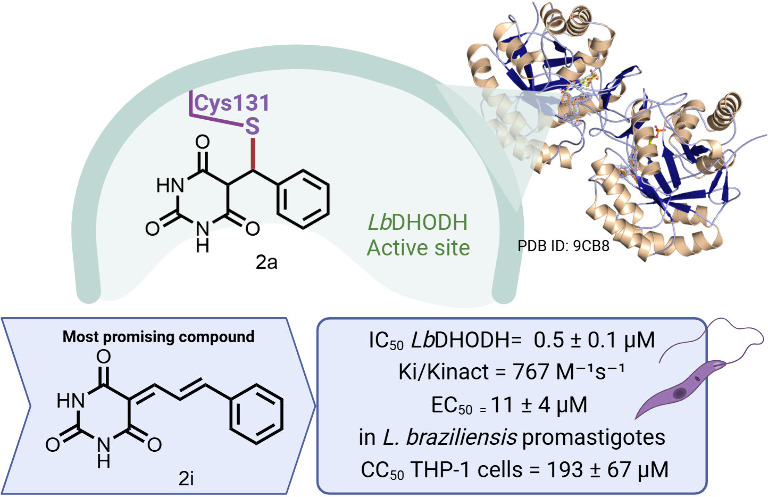

## Introduction

Historically, drug discovery prioritized covalent interactions with druggable targets, aiming for potent and selective inhibitors with prolonged target residence time.^1^ However, early concerns over the selectivity and off-target effects of covalent inhibitors, exemplified by nitrogen mustards and platinum-based chemotherapeutics, led to their exclusion from high-throughput and virtual screening campaigns.^1–5^ Exceptions like penicillins and acetylsalicylic acid, though not intentionally designed as covalent drugs, demonstrated their safety and efficacy.^6,7^ A deeper understanding of enzymatic mechanisms catalyzed a resurgence in covalent drug design, enabling the rational development of suicide inhibitors and mechanism-based electrophiles.^8,9^ This shift is reflected in the growing clinical impact of covalent drugs, with at least eight FDA approvals since 2013, primarily in oncology, though recent successes include covalent antivirals (nirmatrelvir, telaprevir) and beta-lactamase inhibitors (relebactam).^10^

Since covalent drugs offer a novel approach to targeting both druggable and potentially undruggable targets,^13^ efforts have also been directed toward developing covalent inhibitors as a strategy for discovering treatments for neglected tropical diseases, which urgently require new therapeutic options to address their significant global health burden.^14^ For example, *Trypanosoma brucei* targets such as trypanothione reductase,^11^ pteridine reductase 1,^12^ and GAPDH^13^ have been effectively targeted using highly potent inhibitors featuring reactive electrophilic warheads. Similarly, covalent inhibition has been achieved for *Trypanosoma cruzi* cruzain,^14^ as well as *Plasmodium falciparum* falcipain^15^ and FK506-binding protein,^16^ using electrophilic warhead-containing compounds. Notably, most efforts to develop covalent drugs against leishmaniasis have focused on cysteine protease inhibitors.^17^ For instance, azadipeptide nitrile has shown promising inhibitory activity against *L. mexicana* cysteine protease,^17^ while pyrrolidinone-containing peptide, aldehydes and vinyl sulfones targeting *L. major* cathepsin B-like cysteine protease have been previously described.^18^ To the best of our knowledge, no covalent drug design efforts have been explored for *Leishmania braziliensis* (*Lb*), one of the primary causative agents of cutaneous leishmaniasis (CL) in Latin America, alongside *Leishmania amazonensis*.^19^ This region reports approximately 54,000 new cases annually.^20^ Although cutaneous leishmaniasis (CL) is rarely fatal, the skin lesions it causes often lead to lifelong scarring, which can severely affect patients’ quality of life.^21^ Despite affecting 600,000 to 1 million people worldwide each year,^22^ current treatments for cutaneous leishmaniasis (CL) remain limited to a few drugs - pentavalent antimonials, pentamidine, miltefosine, and amphotericin B - which are associated with low efficacy and significant safety concerns, including urticaria, hepatotoxicity, and cardiotoxicity.^23^ Additionally, the increasing emergence of drug-resistant strains further complicates treatment, highlighting the urgent need for new therapeutic options. This growing resistance presents a significant challenge in managing leishmaniasis effectively.^24^ Despite the urgent need for new, effective, and safer treatments for CL, the global development pipeline for leishmaniasis remains scarce. DNDi, one of the leading product development partnerships (PDP) in the field, has seven new chemical entities in its clinical portfolio, mostly targeting visceral leishmaniasis.^25^

As part of our ongoing efforts to develop preclinical and clinical candidates for CL, we focused on the pyrimidine pathway, a key metabolic route in both mammalian and microbial cells, essential for DNA and RNA biosynthesis.^26^ This pathway has been widely studied as a drug target for various diseases that depend on cellular pyrimidine resources.^27^ Among its key enzymes, dihydroorotate dehydrogenase (DHODH) is a flavoenzyme that catalyzes the fourth step and serves as a rate-limiting factor in the de novo pyrimidine biosynthesis of parasites. It facilitates the stereospecific oxidation of the (*S*)-dihydroorotate (DHO) isomer to orotate (ORO) via a ping-pong mechanism (Figure 1a).^28^ The biochemical significance of the pathway involving DHODH, coupled with the distinct classification of DHODH from *Leishmania braziliensis* (*Lb*DHODH) as a class 1A homodimeric enzyme located in the cytosol, contrasting with the human class 2 monomeric enzyme found in the inner mitochondrial membrane, suggests that *Lb*DHODH-selective inhibitors could serve as promising lead compounds (Figure 1S and 2S).^28^ Moreover, *Lb*DHODH possesses a nucleophilic cysteine residue (Cys^131^) as its catalytic residue, whereas human DHODH (*Hs*DHODH) features a serine (Ser^215^) at the equivalent position.^28^

**Figure 1:**
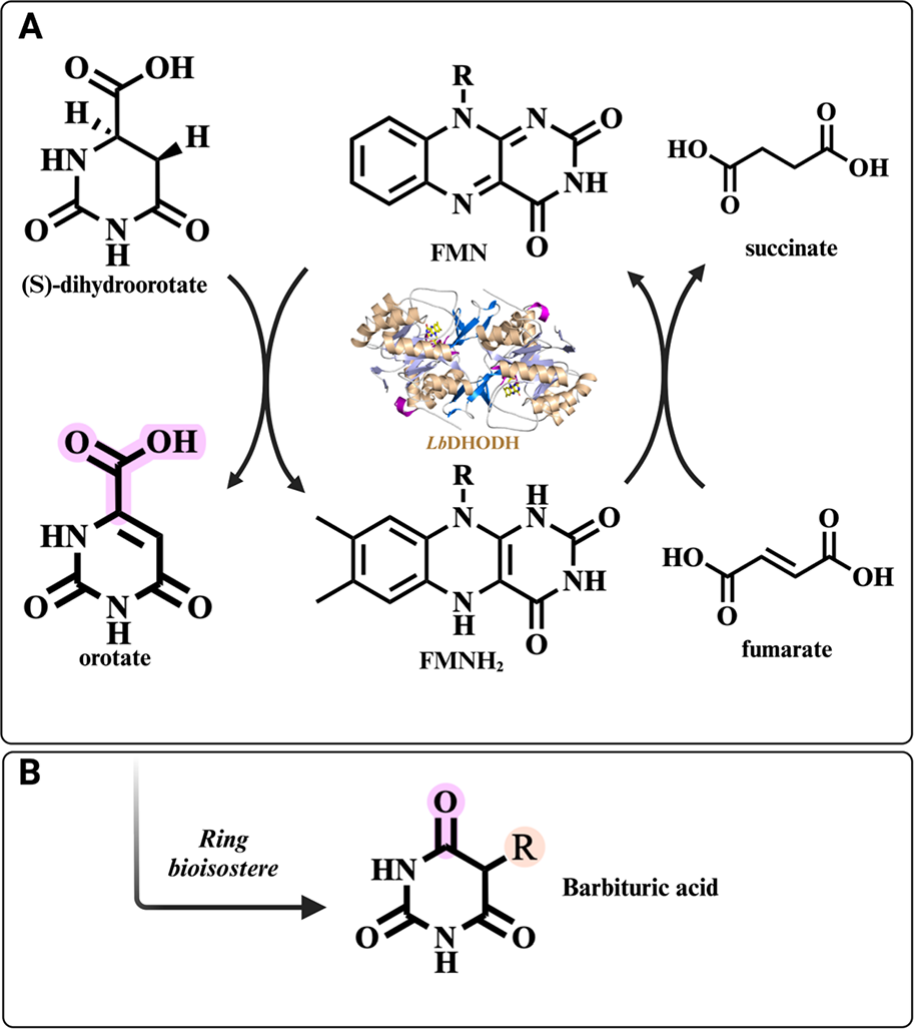
Ping-pong catalytic mechanism of *Lb*DHODH and BARB ring as an orotate bioisostere. (a) Following a ping-pong mechanism, in the first reaction, dihydroorotate is oxidized to orotate, whereas the prosthetic group flavin mononucleotide (FMN) is reduced to FMNH_2_. In the next half-reaction, FMNH_2_ is converted to FMN by the fumarate, which acts as electron acceptor. (b) BARB serves as a bioisostere of orotate, with the carbonyl group highlighted in pink and position 5, where the substituents on the BARB ring are attached, highlighted in orange. The cartoon representation of *Lb*DHODH (PDB ID: 4WZH) is shown at the center, created using PYMOL.

**Figure 2:**
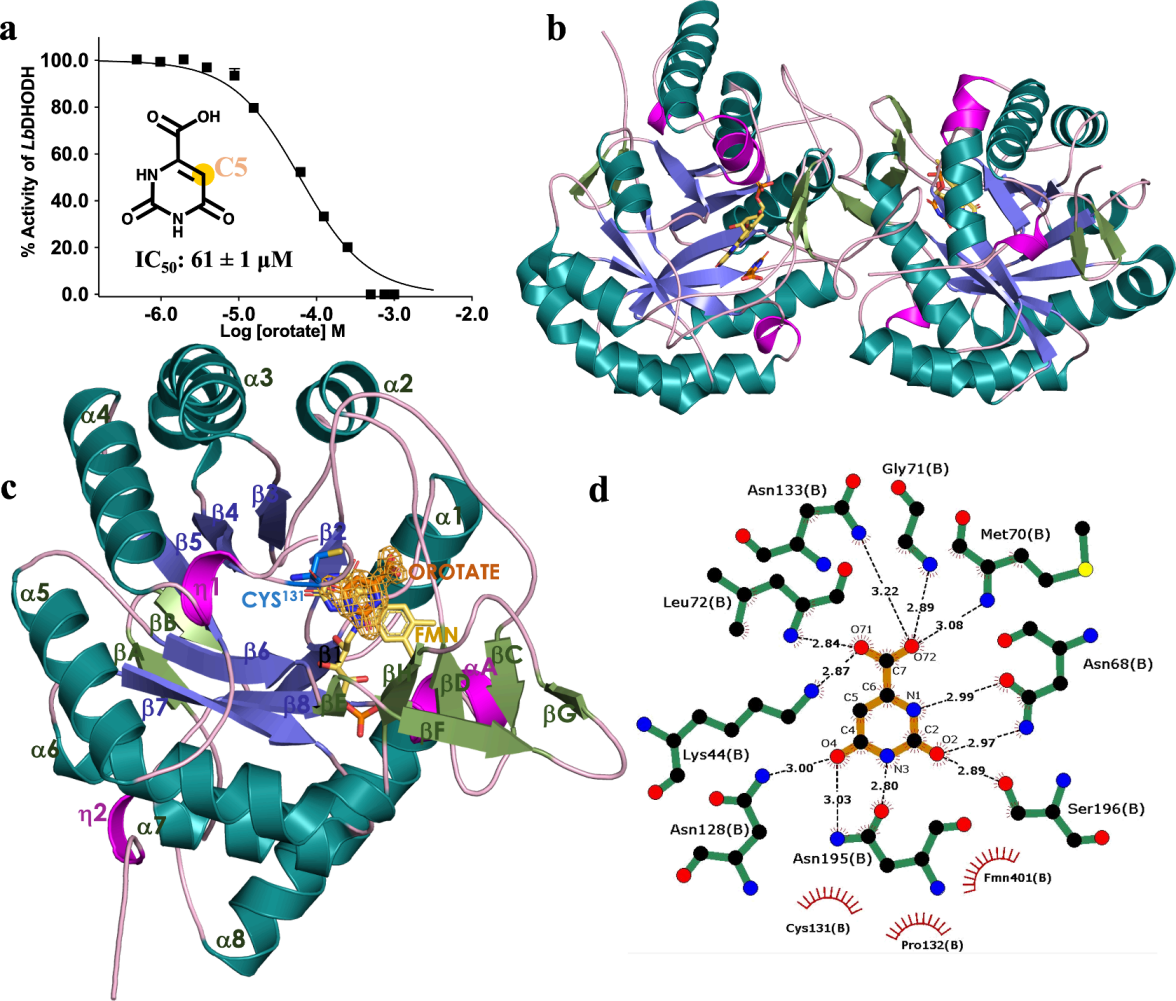
Comprehensive analysis of ORO binding to *Lb*DHODH: concentration–response, structural representations, and interaction schematic. (A) Concentration–response curve with calculated IC_50_ values and the chemical structure of orotate, highlighting the C5 position. (B, C) Ribbon representations of *Lb*DHODH in complex with orotate. The prosthetic FMN group is depicted in yellow, while organic compounds are shown in stick format. Panel (b) illustrates the native functional dimer present in the asymmetric unit. Panel (c) presents the monomeric structure, which comprises a central barrel formed by eight parallel strands (1 – 8, shown in light purple) surrounded by eight helices (1 – 8, shown in green). Two N-terminal antiparallel strands (βa – βb) are located at the bottom of the barrel, and additional secondary-structural elements and loops form a protruding subdomain at the top of the barrel (colored in light green, pink, and light pink). ORO is depicted in orange, and the refined 2*Fo-Fc* electron density map (contoured at 1.0 RMSD) appears as a brown mesh around orotate. The catalytic cysteine is represented in blue stick format. (D) Schematic diagram of the interactions between *Lb*DHODH and ORO. Atoms are color-coded as follows: oxygen in red, nitrogen in blue, carbon in blue for residues, and green for ORO. Hydrogen bonds are shown as green dashed lines. Image created with LigPlot+.^31^

These key differences provide an opportunity to achieve selectivity through the rational incorporation of structural modifications. In general, cysteine residues are suitable for labeling with targeted covalent inhibitors, but steric hindrance and electronic requirements within the active site can make designing selective inhibitors far from trivial.^29,30^ In our study, we aim to develop covalent inhibitors that selectively bind to the catalytic cysteine of *Lb*DHODH. Class 1A DHODH enzymes possess multiple free cysteines; specifically, *Lb*DHODH contains seven. To achieve selectivity for the catalytic cysteine, we hypothesized that exploring bioisosteres of orotate, the product of the first catalytic step (Figure 1a), in combination with a group susceptible to nucleophilic attack by the catalytic cysteine, could be effective. In this work, we present our findings on barbituric acid (BARB, Figure 1b), which was predicted to mimic the interactions of orotate within the enzyme’s active site. This design directs the warhead, chosen as an exocyclic double bond adjacent to the lactam moiety in the BARB ring, toward selective attack by the catalytic cysteine (Figure 1b).^3^

In this study, we integrated biochemical analyses with advanced structural data on ORO binding and BARB derivatives to guide the structure-based design of covalent *Lb*DHODH inhibitors. The *in vitro* inhibition profiles of the most promising compounds were thoroughly characterized by IC_5__0_ and K_inact_/K*I* measurements, while the molecular basis of their interactions with the target was validated by X-ray crystallography. X-ray structures of *Lb*DHODH in complex with ORO and BARB derivatives provided key insights into the structural requirements to find covalent inhibitors. The biological activity of these compounds was demonstrated by their inhibition of *L. braziliensis* promastigote growth, and further corroborated by their low cytotoxicity in THP-1 cells and characterized ADME profiles.

## Results and Discussion

### Biochemical and Structural Characterization of *Lb*DHODH Inhibition by ORO

Considering our initial hypothesis, that exploiting bioisosteres capable of mimicking ORO interactions within the *Lb*DHODH active site could selectively position the warhead for attack by the catalytic cysteine, our first step was to fully characterize the ORO-*Lb*DHODH system using biochemical and structural approaches. ORO was identified as an *Lb*DHODH inhibitor with an IC_50_ of 61 ± 1 μM (Figure 2a), a significant inhibitory activity given the fragment-like size of orotate. Next, to validate the mechanism of interaction between *Lb*DHODH and ORO and to identify specific moieties for potential modification to improve inhibitor potency, we solved the crystal structure of *Lb*DHODH in a complex with orotate.

The structure of the *Lb*DHODH-orotate complex was determined by X-ray crystallography using ammonium sulfate as the precipitating agent. The protein crystallized in the *P*2_1_2_1_2_1_ space group, and the three-dimensional coordinates have been deposited in the Protein Data Bank (PDB) under the accession number PDB ID: 9N67. Data collection parameters and structure refinement statistics are summarized in Supplementary Table S1.

The polypeptide chains of the dimeric *Lb*DHODH–orotate complex (chains A and B) exhibit well-defined electron density for residues Ser^0^ to Met^312^, with the exception of the catalytic loop, residues 135–138 in chain A, 135–136 in chain B, and Asp^313^ in both chains, which were not included in the final refined model due to the absence of clear electron density. This lack of density underscores the intrinsic flexibility of this region, which is functionally important for regulating access to the active site. Nevertheless, the electron density for the catalytic residues is well-defined, allowing the orotate binding profile to be accurately identified and ensuring that this limitation did not affect our analysis (Figure 2).

In addition, the final model contains two FMN, two ORO and three glycerol molecules, four sulfate ions, and 588 solvent sites treated as water oxygens. Each monomer of *Lb*DHODH-ORO displays the characteristic α/β-barrel fold, with a central barrel composed of eight parallel β-strands (β1−β8) surrounded by eight α-helices (α1−α8). Two short antiparallel β-strands, βA and βB, are located at the bottom of the barrel. The flavin binding site is situated between the top of the barrel and the subdomain formed by three antiparallel β-strands, βC, βD, and βH (Figure 2).^30^

The analysis of ORO binding profile reveals stacking interactions with FMN and an extensive hydrogen bonding network (Figure 2d). The oxygen atom (O71) of the ORO carboxylic acid group is an acceptor in hydrogen bonds with the nitrogen atoms of Lys^44^ (NZ) and Leu^72^ (N). Similarly, the other oxygen at carboxylate group (O72) is an acceptor of a hydrogen bond with the main chain nitrogen atoms of Met^70^, Gly^71^, and Asn^133^. ORO atoms N1 and O2, as well as N3 and O4, form hydrogen bonds with the oxygen (OD1) and nitrogen (ND2) of Asn^68^ and Asn^195^, respectively. ORO atoms O2 and O4 also participate in hydrogen bonds with the oxygen (OG) and nitrogen (ND2) of Ser^196^ and Asn^128^, respectively.

There are minimal changes in the three-dimensional structure when comparing the *Lb*DHODH-apo (PDB ID:4WZH)^32^ and *Lb*DHODH-ORO complex (PDB ID: 9N67), with an average Cα atoms RMSD of only 0.24 Å. This small deviation indicates that the overall protein structure is largely conserved upon ORO binding, whereas the largest differences are found at the catalytic loop (Leu^129^–Try^142^), which is completely absent in the apo structure. The dimeric structure of *Lb*DHODH complexed with orotate closely resembles that of other class 1 DHODH enzymes, such as *Leishmania major* DHODH (*Lm*DHODH, PDB ID: 3GZ3 ^33^) and *Trypanosoma cruzi* DHODH (*Tc*DHODH, PDB ID: 2E6A^34^). Superimposing the Cα atoms of all residues in the *Lb*DHODH-ORO, *Lm*DHODH-ORO, and *Tc*DHODH-ORO structures yields RMSD values of 0.80 Å for *Lm*DHODH and 0.67 Å for *Tc*DHODH. The most notable differences occur in the catalytic loop (residues Leu^129^ to Tyr^142^), which is present in *Tc*DHODH-ORO but absent in *Lm*DHODH-ORO and partially modeled in *Lb*DHODH-ORO structure.

### Compound Design and Synthesis

BARB moiety (Figure 1b) was previously identified as a competitive inhibitor of DHO in human DHODH.^35^ Building on this, we investigated the BARB ring as a potential ORO bioisostere for *Lb*DHODH. This was rationalized by the BARB scaffold’s three carbonyl groups, which can serve as hydrogen-bond acceptors, analogous to the carboxylate and two carbonyls of ORO (Figure 2d). The C5 position is critical in the catalytic mechanism of class 1 DHODHs, being directly involved in oxidizing the C5–C6 double bond of DHO to ORO. Consequently, modifications at this catalytically essential site can diminish enzyme activity, as demonstrated for *Lm*DHODH^36^ and *Tc*DHODH.^37^ Notably, these enzymes share 100% sequence identity at the active site with *Lb*DHODH, suggesting that this enzyme active site can also accommodate bulky substituents at the C5 position of the BARB ring (Figure 2a).

To evaluate BARB as an ORO bioisostere and to identify optimized and selective *Lb*DHODH inhibitors, we designed a series of compounds intended to mimic ORO while incorporating an electrophilic warhead at the crucial C5 position of the BARB ring. This compound series was systematically designed, starting with the parent structure **2a**, to elucidate the impact of C5-substituent diversity on binding interactions and electrophilic reactivity. Accordingly, we incorporated electron-donating groups (EDGs; **2c**, **2g**), electron-withdrawing groups (EWGs; **2b**), and substituents with mixed electronic properties (**2d**, **2e**) at the benzene ring to modulate warhead reactivity (as discussed in the intrinsic reactivity section). Furthermore, to probe the conformational flexibility of the *Lb*DHODH active site and its capacity to accommodate larger moieties, substituents such as *p*-morpholine (**2e**), biphenyl (**2f**), a pyrrole-containing biaryl (**2h**), and conjugated systems (**2i**) were introduced.

Compounds **2a–2i** were synthesized by reacting the heterocycle with various aldehydes via Knoevenagel condensation, introducing an exocyclic double bond at **C-5** (Scheme 1). Attempts to reduce compound **2d** were unsuccessful, resulting in a complex mixture of products. These compounds were then reduced to generate the benzylic derivatives **3a**–**3c** and **3e-3ii** (Scheme 1). The design of this series was inspired by previous studies showing that exocyclic double bonds in BARB rings are prone to nucleophilic thiol addition,^38^ allowing us to evaluate **2a–2i** as potential covalent inhibitors of *Lb*DHODH. To assess the impact of spacers, we also synthesized compound **2i**, incorporating a diene spacer between the two rings using the same strategy. In these compounds, the Michael acceptor moiety is expected to be optimally positioned for nucleophilic attack by Cys^131^. Conversely, the reduced compounds **3a-3c, 3d–3h and 3ii**, which lack the exocyclic double bond, are anticipated to act as noncovalent inhibitors. All synthesized compounds were comprehensively characterized by NMR spectroscopy (All spectra are available in the Supporting Information). The absence of the Michael acceptor moiety in the reduced compound series **3a**–**c** and **3e-3ii** was unambiguously confirmed through comprehensive ^13^C and ^1^H NMR analysis. The characteristic presence of a proton peak (**3a**-**3ii,** H^1’^, Scheme 1) corresponding to the methylene at C-5 position in ^1^H NMR spectra confirms the hybridization change from sp^2^ to sp^3^ to at this carbon center. Notably, the hydrogen in the exocyclic double bonds exhibits a characteristic singlet with chemical shifts ranging from 8.3 to 8.9 ppm in the warhead-containing compounds (**2a**-**2i**, Scheme 1), whereas the corresponding proton connected to the sp^3^ carbon at the analogous position in the reduced derivatives displays significantly upfield chemical shifts ranging from 2.8 to 3.8 ppm. These substantial differences in peaks and chemical shifts values provide definitive confirmation of the successful removal of the covalent warhead functionality.

**Scheme 1:**
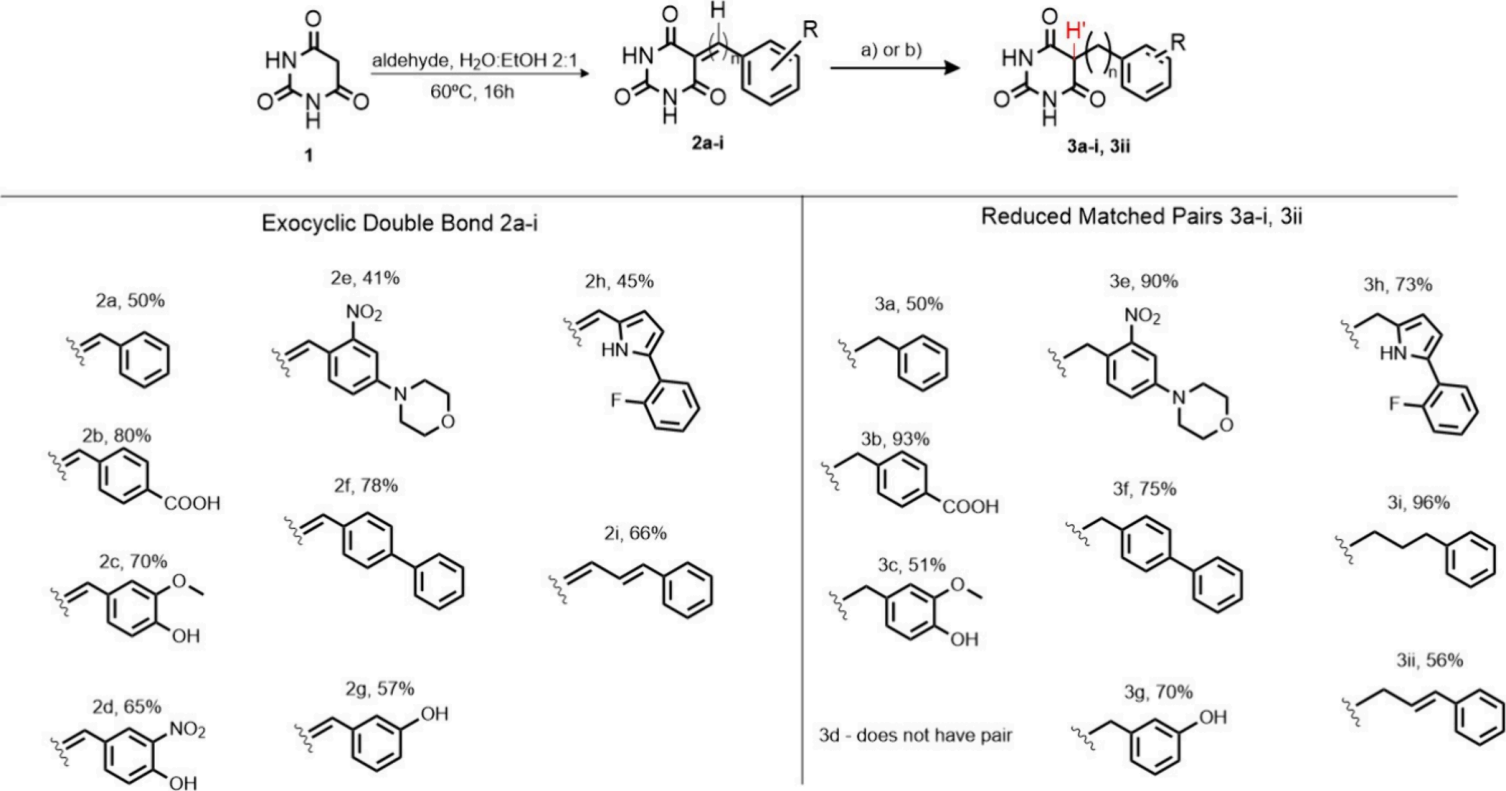
Synthesis of barbituric acid derivatives: (a) NaBH_4_, EtOH, 15 min (**3a–3c**, **3e–3i**); or (b) Pd/C, H_2_, EtOH, 15 min (**3ii**)

Furthermore, comparing compounds **2a–2i** with their more flexible, molecularly matched counterparts **3a-3c, 3d–3ii** provides valuable insights into active-site complementarity and clarifies the structural features necessary for effective *Lb*DHODH inhibition.

### Evaluation of **2a**–**2i** as *Lb*DHODH and *Hs*DHODH Inhibitors

Covalent inhibitors exhibit time-dependent potency because their effectiveness relies on the rate of covalent bond formation.^39^ To assess the ability of compounds **2a–2i** to inhibit *Lb*DHODH via covalent bond formation with Cys^131^, we initially screened them at a concentration of 100 μM under two conditions: with and without a 4-h preincubation with the enzyme. The *Lb*DHODH inhibitory activity assay was performed using an indirect method that monitors DCIP reduction at 610 nm, a process that is stoichiometrically equivalent to orotate formation.^28,40^ For compounds showing at least 50% inhibition of *Lb*DHODH activity, the concentration–response curves were determined, and IC_50_ values were calculated (Table 1).

**Table 1:** Inhibitory Profile against *Lb*DHODH and Leishmanicidal Activity of BARB Derivatives^a^

ID	single dose (0 h)* % activity	single dose (4 h)* % activity	IC_50_ 0 h (μM)	IC_50_ 4 h (μM)	K_*inact/*_KI (M^–1^s^–1^)	EC_50_ (μM)	CC_50_ (μM)
**2a**	11 ± 9	3.5 ± 0.2	56 ± 5	13 ± 2	17	>200	>200
**2b**	70 ± 10	10 ± 2	>100	22 ± 1	ND	>200	>200
**2c**	7 ± 3	21 ± 5	7 ± 1	89 ± 4	ND	>200	>200
**2d**	5 ± 2	16 ± 3	3 ± 1	13 ± 2	73	152 ± 28	>200
**2e**	10 ± 1	7 ± 2	5 ± 1	28 ± 3	ND	17 ± 11	>200
**2f**	62 ± 2	3.8 ± 0.1	160 ± 10	7.7 ± 0.3	ND	19 ± 14	12 ± 3
**2g**	22 ± 4	8 ± 2	90 ± 10	13 ± 1	41	>200	76 ± 12
**2h**	90 ± 10	60 ± 10	>100	>100	ND	5 ± 2	>200
**2i**	1.1 ± 0.2	3 ± 2	0.9 ± 0.3	0.5 ± 0.1	767	10.5 ± 3.5	193 ± 67
**3a**	90 ± 10	70 ± 8	>100	>100	NA	>200	>200
**3b**	109 ± 3	69 ± 2	>100	>100	NA	>200	>200
**3c**	98 ± 7	60 ± 10	>100	>100	NA	>200	>200
**3e**	90 ± 10	80 ± 20	>100	>100	NA	23 ± 16	>200
**3f**	83.0 ± 0.2	34 ± 6	ND	ND	NA	>200	>200
**3g**	93 ± 5	36 ± 3	ND	ND	NA	>200	>200
**3h**	80 ± 10	80 ± 20	>100	>100	NA	>200	>200
**3i**	103 ± 3	21 ± 7	50 ± 10	>100	NA	>200	193 ± 67
**3ii**	98 ± 5	21 ± 7	ND	ND	NA	23 ± 6	>200
**Orotate**	35 ± 4	27 ± 2	57 ± 2	27 ± 1	NA	ND	ND
**Amp. B**	NA	NA	NA	NA	NA	0.4 ± 0.3	6 ± 2

a *Compounds were tested at 100 μM; NA: not applicable; ND: not determined; EC_50_ determined for *Leishmania braziliensis* promastigotes; CC_50_ determined for the THP-1 cell line. Orotate and Amphotericin B (Amp. B) was used as control.

Single-dose screening of compounds **2a**-**2i** revealed time-dependent inhibition patterns, suggesting potential covalent interactions with *Lb*DHODH. Compounds **2b** (*p*-benzoic acid substitution) and **2f** (biphenyl substitution) demonstrated the most significant increase in inhibition between 0 and 4 h assays (Δ*a*ctivity ≈ 60 μM). However, **2c** and **2d**, containing 4-hydroxy-3-methoxy-benzylidene and 4-hydroxy-3-nitro-benzylidene substituents respectively, showed slightly decreased inhibition after incubation (Δ*a*ctivity ≈ −14 μM and −11 μM).

Most compounds exhibited potent enzyme inhibition (>90%), with **2i** (phenylallylidene), **2a** (benzylidene), and **2f** (biphenyl) showing the highest levels at 97%, 96.5%, and 96.2%, respectively. Other benzylidene derivatives (**2b**, **2e**, and **2g**) demonstrated comparable activity (90–93% inhibition). Calculated IC_50_ after 4 h preincubation identified **2i** as the most potent inhibitor (0.5 ± 0.1 μM), maintaining similar potency to 0 h assays (0.9 ± 0.3 μM). The remaining compounds showed IC_50_ values ranging from 7.7 ± 0.3 μM (**2f**) to 89 ± 4 μM (**2c**), while **2h** was inactive under assay conditions.

Preliminary analysis revealed several key features affecting inhibitor potency. The electron-rich pyrrole moiety in **2h** led to a complete loss of activity, while the extended conjugation in **2i** enhanced potency, possibly through increased molecular volume or altered nucleophilic attack sites. Disubstituted compounds (**2c**, **2d**, and **2e**) showed higher potency than the unsubstituted **2a** without incubation, but demonstrated reduced activity after 4h. Interestingly, compounds **2c**–**e** contain EDG substituents on the benzene ring, which may modulate the electrophilicity of the warhead and consequently affect their reactivity with the catalytic cysteine residue, thereby impacting the stability of the covalent inhibitor-enzyme adduct and ultimately influencing their inhibitory profiles. Comparison with reduced analogs (**3a**-**3c**, **3e**-**3i**) further supported the importance of the exocyclic double bond for activity. Despite containing the barbituric acid moiety, reduced compounds were largely inactive, suggesting the Michael acceptor system’s crucial role in binding to Cys^131^. This observation aligns with the rational design strategy, while the enhanced potency of high-molecular-volume substituents (diene **2i**, biphenyl **2f**) emphasizes the importance of substitution patterns (Table 1).

All synthesized compounds were tested against the human ortholog *Hs*DHODH to evaluate their selectivity for *Lb*DHODH. Interestingly, none of the compounds exhibited significant inhibition (>50%) in single-dose assays at 100 μM, either with or without a 4-h incubation (Table 2S). This finding highlights the potential of barbituric acid derivatives as selective inhibitors of *Lb*DHODH.

**Table 2:** ADME Studies of Compounds **2c**, **2d**, **2e**, **2h**, and **2i** ^a^

ID	*c*logP*	kinetic solubility PBS pH 7.4 (μM)	permeability (nm/s)	mouse liver microsomes *T*-half (min)	mouse liver microsomes CL_int,_ app (μL/min/mg)
**2c**	0.4	194.64	8.4	6.0	290.4
**2d**	0.5	190.05	n.d.	n.d.	n.d
**2e**	0.5	178.35	n.d.	>120.0	<14.4
**2h**	2	4.63	15.4	<3.0	>577.5
**2i**	1.7	41.38	57.5	<3.0	>577.5

a n.d. not determined, due to instability in the assay matrix or poor detection sensibility in LC/MS. *Calculated with CDD Vault (Collaborative Drug Discovery Ltd.).

### Intrinsic Reactivity Studies

The K_inact_/Ki ratio is a key parameter for evaluating the potency of covalent inhibitors, as it quantitatively measures both their reactivity and affinity for the target enzyme.^41^ This parameter integrates two crucial aspects: KI, which reflects the inhibitor’s binding affinity, and K_inact_, which describes the rate at which the covalent bond is formed. A high K_inact_/KI value indicates that a compound not only binds effectively but also rapidly forms a stable covalent adduct, leading to sustained enzyme inhibition.^42^ Therefore, this ratio is essential for ranking covalent inhibitors, optimizing their potency, and guiding structure-based drug design.

For the most potent *Lb*DHODH inhibitor, **2i**, the calculated K_inact_/KI ratio is 767 M^–1^s^–1^, which is approximately ten times higher than that of **2d** (73 M^–1^s^–1^) and 19 times higher than **2g** (13 ± 1 M^–1^s^–1^). A structural comparison among these inhibitors, combined with IC_50_ and strong difference in K_inact_/KI values, strongly suggests that potency within this series is directly correlated with their reactivity. For example, the extended conjugation in compound **2i** enables a 1,6-Michael addition pathway, enhancing its reactivity profile, while the electron-rich aromatic system in **2d** and **2g** decreases the electrophilicity of the exocyclic double bond, thereby reducing its susceptibility to nucleophilic attack.

Therefore, to better understand the reactivity of BARB derivatives with *Lb*DHODH catalytic Cys^131^, we employed density functional theory (B3LYP/TZ2P) to optimize the ground-state geometry and examine the frontier molecular orbitals (HOMO/LUMO) of representative barbituric derivatives **2a** and **2i** (Figure 3 and Supporting Information).^43^ Comparing **2a**, the simplest derivative, with **2i**, the most potent diene, highlights distinct differences in their frontier orbitals. In **2a**, the HOMO is largely localized at C-5 of the barbiturate ring, whereas the LUMO (approximately 30%) resides on the exocyclic double-bond carbon (C-1) linked to the phenyl ring. By contrast, **2i** displays a π-type HOMO delocalized across the diene spacer, while its LUMO receives notable contributions from the C-1 linked to the phenyl ring (17%) and C-3 (17%) double-bond carbons adjacent to the barbituric ring (∼8%). This distribution indicates that both π-antibonding orbitals in the diene have comparable susceptibility to nucleophilic attack by the catalytic cysteine thiol. Notably, **2i** also has a more negative LUMO (−3.31 eV) than **2a** (−2.96 eV), suggesting a smaller energy gap with the thiolate HOMO of Cys^131^ and thus favoring nucleophilic attack. Although additional factors (e.g., partial charges, molecular conformation, solvation) can further modulate reactivity, these combined insights from orbital localization and LUMO energies strongly support **2i** as the more electrophilic Michael acceptor, which is coherent with highest ability of this compound to inhibit *Lb*DHODH. Analysis of the remaining derivatives (**2d** and **2g** - Supporting Information) revealed frontier orbital characteristics broadly like those of **2a**, helping to explain their weaker inhibitory potencies compared to the more delocalized and potent **2i.**

**Figure 3:**
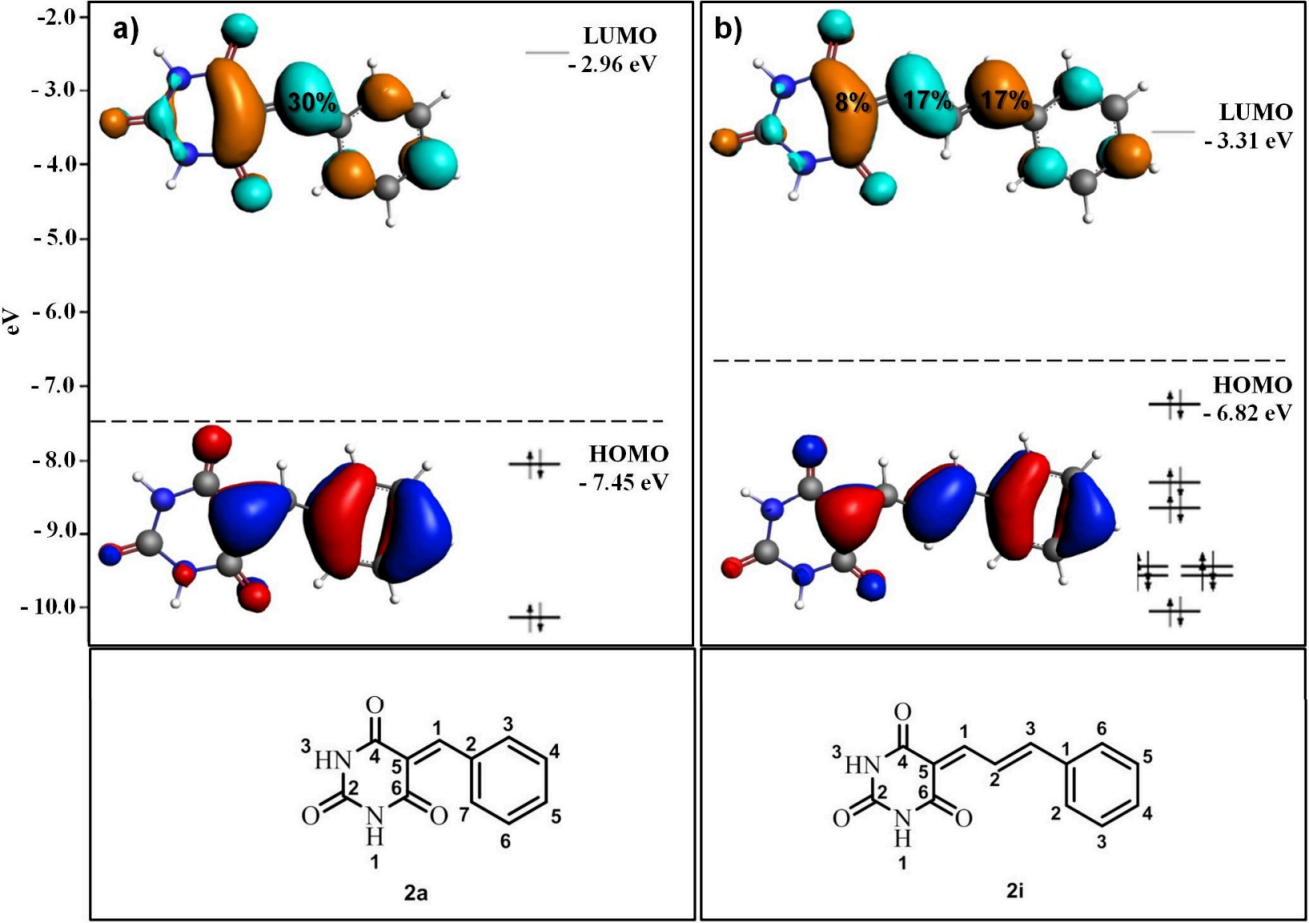
Computed frontier molecular orbital energy levels for BARB derivatives **2a** (A) and **2i** (B). The HOMO–LUMO energy gaps demonstrate the influence of conjugation and substitution patterns on electronic properties. Calculations were performed at the B3LYP/TZ2P level of theory. Energies are reported in electron volts (eV) relative to vacuum.

### Structural Characterization of Barbituric Acid Derivatives as *Lb*DHODH Inhibitors

Although our results highlight the importance of the reactivity of the covalent warhead with Cys^131^ for *Lb*DHODH inhibition, structural studies were conducted to further evaluate the binding mode and complementarity of these inhibitors within the enzyme’s active site. To elucidate the interaction of BARB derivatives with *Lb*DHODH, compounds **2a**, **2c**, **2g**, and **3c** were cocrystallized with the enzyme under similar conditions as described for orotate. The crystal structures were determined by molecular replacement using 4WZH^32^ as the search model and refined to high resolution, 1.75 Å, 1.9 Å, 1.45 Å and 1.85 Å for **2a**, **2c**, **2g**, and **3c**, respectively, all with excellent geometric validation (Table 1S). The coordinates for the *Lb*DHODH–ligand complexes were deposited in PDB under accession codes PDB ID: 9CB8 (**2a**), 9N6O (**2c**), 9N68 (**2g**), and 9N6Q (**3c**), respectively.

Overall, in all four complexes (**2a, 2c, 2g, and 3c**), the *Lb*DHODH monomer adopts an α/β-barrel fold, consisting of eight parallel β-strands (β1−β8) surrounded by eight α-helices (α1−α8), with two short antiparallel β-strands (βA and βB) positioned at the bottom of the barrel, as previously described for the *Lb*DHODH–orotate complex (Figure 2b). The dimeric structures of these complexes closely resemble that of unbound *Lb*DHODH (PDB ID: 4WZH), with average Cα RMSD values of 0.25 Å. Structural alignment with the *Lb*DHODH–orotate complex (PDB ID: 9N67) yielded average Cα RMSD values of 0.22 Å, indicating a highly conserved overall fold across the complexes.

All *Lb*DHODH complexes (chains A and B) span residues from Ser^0^ to Met^312^. *Lb*DHODH-**2a** contains two FMN cofactors, two **2a** molecules, four glycerol molecules, and 272 solvent sites; *Lb*DHODH-2c includes two FMN cofactors, two **2c** molecules, two glycerol molecules, three sulfate ions, and 148 solvent sites; *Lb*DHODH-**2g** consists of two FMN cofactors, two **2g** molecules, four glycerol molecules, and 457 solvent sites; and *Lb*DHODH-**3c** features two FMN cofactors, two **3c** molecules, one glycerol molecule, five sulfate ions, and 278 solvent sites. All solvent sites were modeled as water oxygens. Due to the absence of clear electron density, the following fragments were omitted from the final models: in *Lb*DHODH-**2a**, Ala^50^–Ala^56^ and Leu^72^ in chain A; in *Lb*DHODH-**2c**, Arg^51^–Gly^53^ and Leu^72^ in chain A; in *Lb*DHODH-**2g**, Leu^51^–Pro^57^ and Gly^71^–Pro^73^ in chain A; and in *Lb*DHODH-**3c**, residues 131–138 in chain A and 134–138 in chain B. Additionally, for all complexes, the C-terminal residue Asp^313^ from both chains was excluded from the final model.

Overall, analysis of the B-factor distribution and per-residue RMSD indicates that chain A is generally more disordered than chain B. As previously described for the unbound *Lb*DHODH structure (PDB ID: 4WZH)^30^, this disorder is attributed to crystal packing, which allows chain B to make contacts not present in chain A. The most pronounced differences occur in the β2- βc connecting loop (cis-proline loop: residues Thr^47^-Arg^58^), the βd-α2 connecting loop (residues Met^70^-Asp^78^), and the catalytic loop (Ser^130^- Gln^139^) (Figure 4). Similar structural features have been reported for other class 1A DHODH including *Lactococcus lactis* DHODH (*La*DHODH)^45^, where the cis-proline loop near the active site is also identified as a flexible region.^41^ Our structural analysis of *Lb*DHODH complexes suggests that the flexibility of the βd−α2 connecting loop (residues Met^70^–Asp^78^) is modulated by direct interactions with the β2−βc loop (residues Thr^48^–Arg^58^) and the catalytic loop (Ser^130^–Gln^139^) (Figure 4b). The latter undergoes conformational shifts to facilitate substrate binding and product release from the active site.

**Figure 4:**
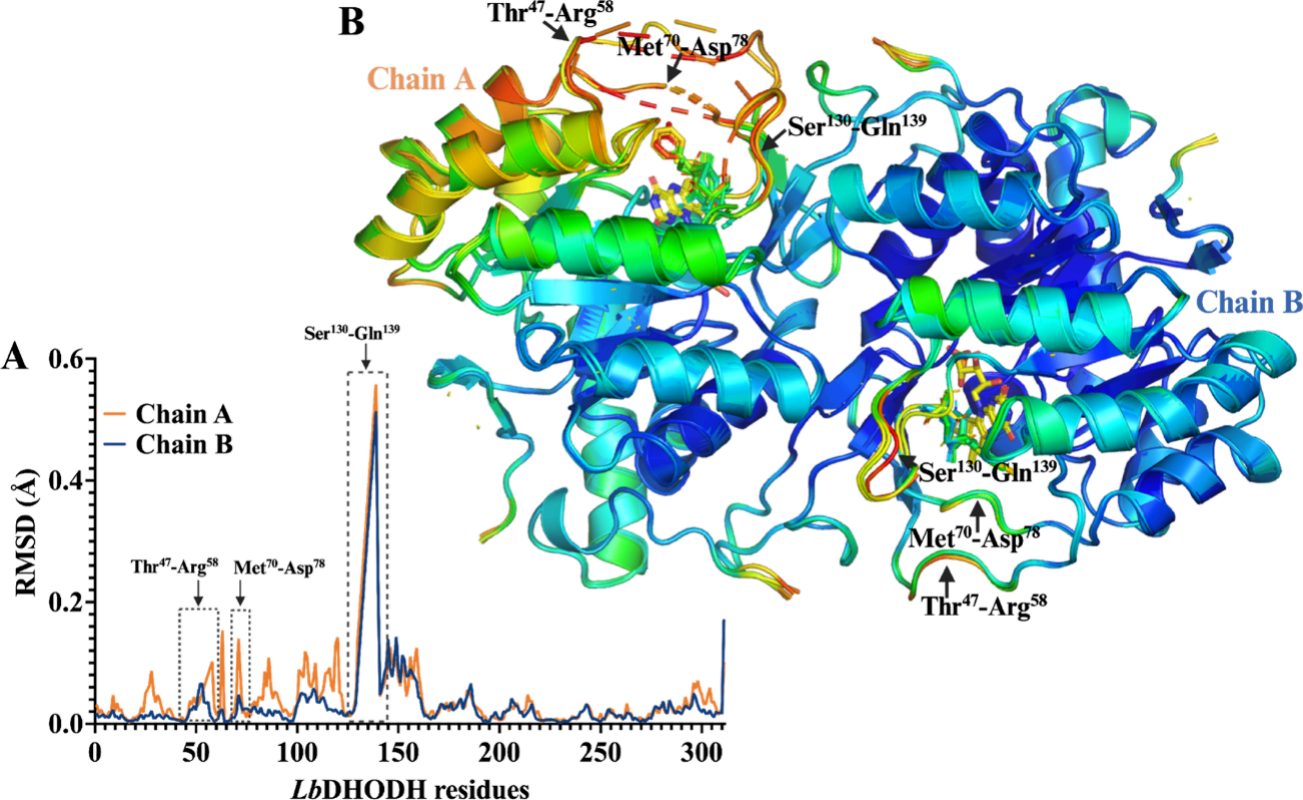
Structural flexibility of *Lb*DHODH analyzed by RMSD and B-factor mapping. (A) Per-residue average RMSD of the Cα positions for chains A (orange) and B (blue) compared to the *Lb*DHODH-orotate complex, highlighting regions of increased structural variability: Thr^48^–Arg^58^, Met^70^–Asp^78^, and Ser^130^–Gln^139^. (B) Cartoon representation of *Lb*DHODH in complex with **2a**, **2c**, **2g**, and **3c**, color-coded by B-factor to visualize flexibility, where dark blue represents low flexibility, green indicates intermediate flexibility, and red denotes high flexibility. The regions with major structural variability are highlighted.

Interestingly, the crystal structures of the *Lb*DHODH complexes reveal well-defined electron density for the BARB core, confirming that it adopts the same hydrogen bonding network as orotate (Figures 2d, **5b, 5d, 5g** and **6b**). The carbonyl groups (O8 and O5), along with the amine groups (N1 and N6), form hydrogen bonds with Asn^68^, Asn^128^, Asn^195^, and Ser^196^ (Figures **2d, 5a,5c,5e** and **6a**). Notably, the absence of the carboxylic acid (O71 and O72 in ORO, Figure 2d) is compensated by a water molecule (W268, following *Lb*DHODH-**2a** complex nomenclature), which bridges the carbonyl group to Leu^72^ and Lys^45^ in the active site. In the crystal structures of *Lb*DHODH in complex with **2a**, **2c**, and **2g**, a continuous electron density is observed between the side-chain thiol of Cys^131^ and the exocyclic moiety of the inhibitors, indicating covalent bond formation (Figure 5). This covalent interaction induces the catalytic loop (residues 130–139) to adopt a closed conformation. The loop closure is driven by a ∼ 2.8 Å displacement of Cys^131^ compared to the *Lb*DHODH-**3c** complex, where the reduced compound binds to the catalytic pocket in a noncovalent manner (Figure 6).

**Figure 5:**
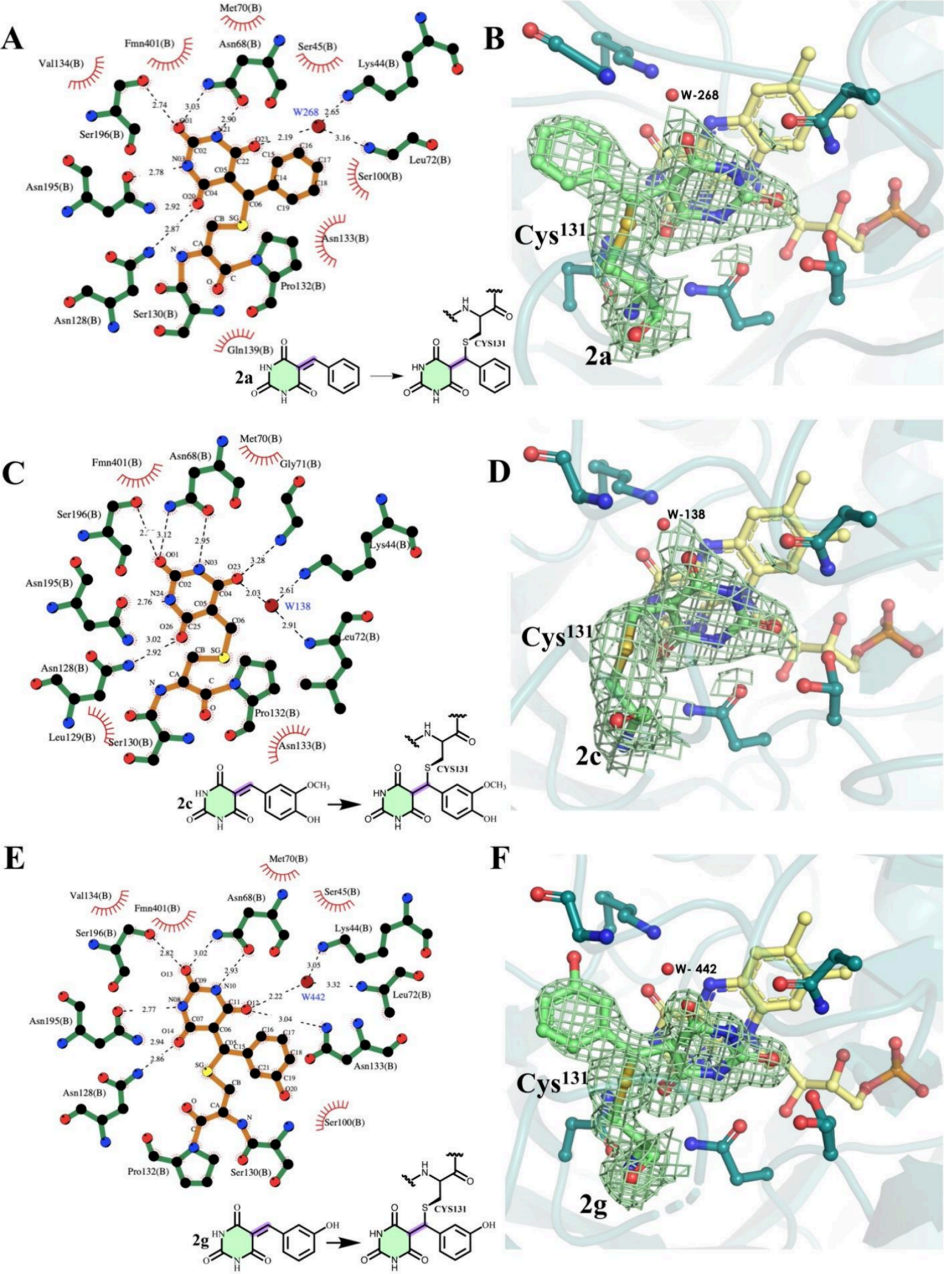
Interaction profile and binding mode of BARB derivatives **2a**, **2c**, and **2g** within *Lb*DHODH active site. The left panels (A, C, E) show schematic representations of key interactions, generated with LigPlot+ 2.2.9.^31^ The right panels (B, D, F) display the crystallographic conformations of the inhibitors. The inset shows a 2D representation of barbituric acid attached to Cys^131^. Atoms are color-coded as follows: oxygen (red), nitrogen (blue), and carbon (green for *Lb*DHODH, orange for inhibitors). Hydrogen bonds are represented by black dashed lines. The 2*Fo-Fc* electron density maps (contoured at 1.0 RMSD) highlight the covalent bond formation between the Cys^131^ side-chain thiol and the exocyclic moiety of inhibitors **2a**, **2c**, and **2g.**

**Figure 6:**
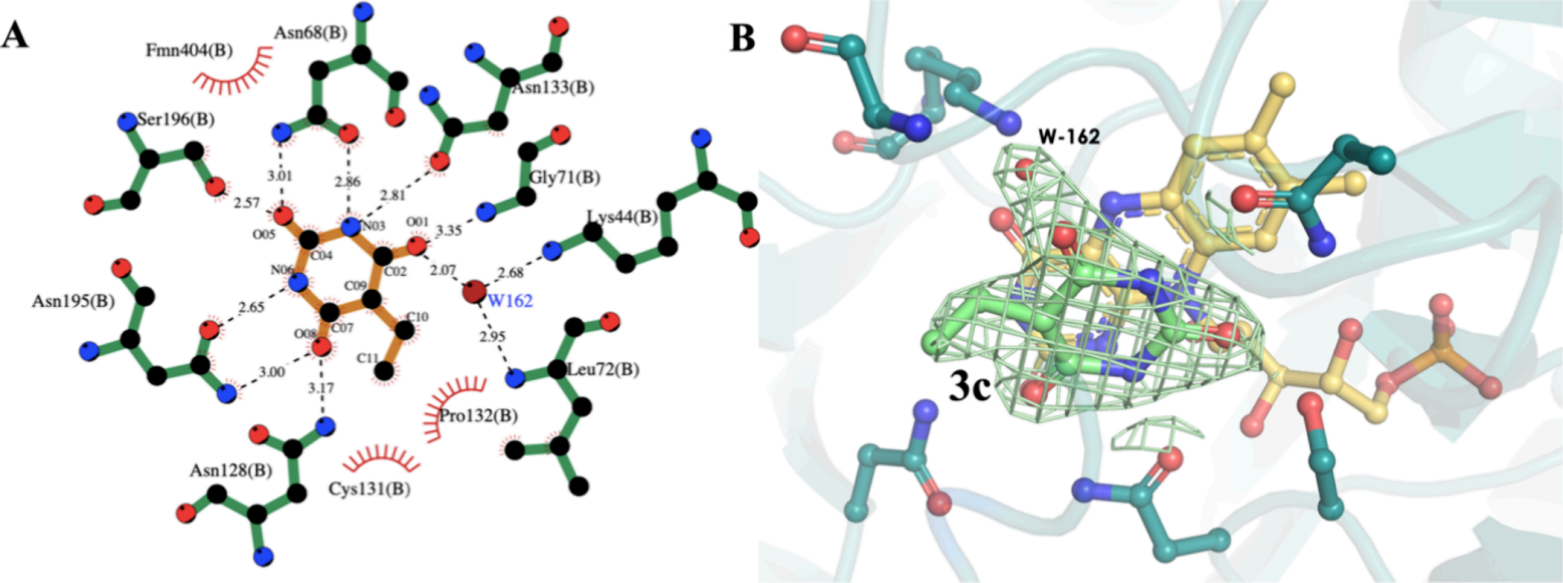
Interaction profile and binding mode of the BARB derivative **3c** within *Lb*DHODH. (A) Schematic representations of key interactions generated using LigPlot+ 2.2.9.^31^ (B) Crystallographic conformation of the inhibitor. The 2*Fo-Fc* electron density map (contoured at 1.0 RMSD) supports the reversible binding mode and indicates that the catalytic loop (residues 130–139) is in an open conformation. The absence of the carboxylic acid group in **3c**, which is present in ORO, is compensated by a bridging water molecule (162), facilitating interactions with Leu^72^ and Lys^45^. Atoms are color-coded as follows: oxygen (red), nitrogen (blue), carbon (green for *Lb*DHODH, orange for inhibitors), and hydrogen bonds are represented by black dashed lines.

In the *Lb*DHODH-**2a** and **-2g** complexes, the side chain of the BARB derivative partially occupies the pocket normally occupied by the side chain of Leu^72^ (chain B), leading to the absence of electron density for the side chain of this residue and highlighting the conformational flexibility of this region. Previous studies on *La*DHODH have demonstrated that Leu^71^, which corresponds to Leu^72^ in *Lb*DHODH, plays a crucial role in positioning the ORO pyrimidine ring above the flavin isoalloxazine. This finding underscores the importance of this residue not only for enzyme catalysis but also for inhibitor binding.^44^ In contrast, the bulky methylene-phenyl ring of **2c** and **3c** appears poorly resolved in both structures, presumably due to its flexibility and due to the lack of interactions with *Lb*DHODH residues.

It is important to highlight that the structure of the *Lb*DHODH–2i complex, involving the most potent inhibitor, was reproducibly obtained with high-resolution data. However, the electron density for the ligand was clearly observed only for the barbituric head and a water molecule, leaving uncertainties regarding both the site of nucleophilic attack, whether 1,4- or 1,6-addition, as both positions are partially supported by the electron density, as well as the orientation of the ring. The ambiguity in the electron density, reproducibly observed across different data sets, while limiting the complete refinement of the data and more detailed assessments of the interaction mechanism, highlights the conformational flexibility of the active site region induced by the ligand, potentially leading to multiple ligand conformations. At this point, we cannot rule out the hypothesis that both 1,4- and 1,6 additions are structurally and chemically possible, and that the ligand may adopt multiple reactive conformations leading to concomitant 1,4- and 1,6 additions.

When we combine the structural findings with the biochemical data on enzymatic inhibition, it becomes evident that the potency of BARB-derived compounds is directly linked to the presence of the exocyclic double bond and its nucleophilic attack by the catalytic cysteine, which locks the active site loop in a closed conformation. The lack of significant interactions involving the side chains of these compounds, despite observable electron density for some, highlights that the potency differences among covalently bound inhibitors are primarily driven by the compound’s reactivity, as evaluated by K_inact_/KI and DFT, with the enzyme rather than enthalpic contributions. This is because, in all cases, the observed interactions are predominantly between the enzyme and the barbituric core. Another key insight from the crystallographic structures is the flexibility of the loop containing Leu^72^, which influences the movement of the cis-proline loop and the catalytic loop (Figure 7). This structural adaptability provides valuable guidance for designing new generations of ligands.

**Figure 7:**
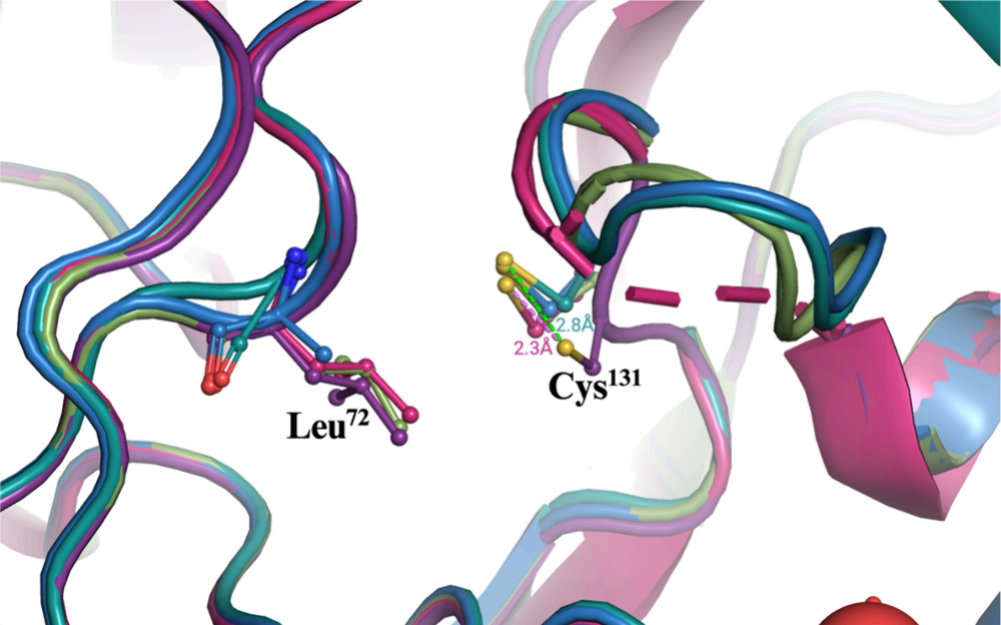
Superposition of five structures resolved in this study, highlighting the conformational variation of the catalytic loop in *Lb*DHODH across different ligand classes. The enzyme was crystallized with ORO (pink, PDB ID: 9N67) and barbiturate-derived ligands featuring an exocyclic double bond as an electrophile: **2a** (dark green, PDB ID: 9CB8), **2c** (olive green, PDB ID: 9N6O), and **2g** (sky blue, PDB ID: 9N68), as well as the reduced compound **3c** (purple, PDB ID: 9N6Q). The cysteine residue in the covalent ligands **2a**, **2c**, and **2g** adopts a conformation similar to that observed with orotate. In contrast, the binding of **3c** positions the cysteine in a conformation that is not optimal for nucleophilic attack on the C5 substituent of the ligands, with a displacement of 2.8 Å from the covalent ligand conformation and 2.3 Å from the cysteine conformation in the orotate complex.

### Leishmanicidal Activity of Barbituric Acid Derivatives

The leishmanicidal activity of all compounds was evaluated against *L. braziliensis* promastigotes, as shown in Table 1, alongside their cytotoxicity in THP-1 monocyte-derived leukemia cells. Of the 18 compounds tested at 200 μM), 12 (**2a**, **2b**, **2c**, **2d**, **2g**, **2j**, **3a**, **3b**, **3c**, **3f**, **3g**, and **3h**) were inactive against the parasite. Six compounds (**2h**, **2i**, **2e**, **2f**, **3e**, **3ii**) exhibited EC_50_ values ranging from 5.4 to 23 μM (Table 1). This is the first evidence that *Lb*DHODH inhibition can translate into antiparasitic activity, although the full characterization of the mode of action of these compounds is warranted to rule out possible interaction with other targets.

Overall, barbituric acid derivatives showed low cytotoxicity. Compounds lacking exocyclic double bonds (**3a-3h, 3ii**) were noncytotoxic in THP-1 cell lines (CC_50_ > 200 μM). By contrast, compounds bearing exocyclic double bonds (e.g., **2f**, **2g**) displayed higher cytotoxicity. In particular, **2f** exhibited strong cytotoxicity and a low selectivity index. However, the most promising *Lb*DHODH inhibitor, **2i** (IC_50_ = 0.5 ± 0.1 μM), retained leishmanicidal activity (EC_50_ of 10.5 ± 3.5 μM) while showing no cytotoxicity. These findings suggest that potent covalent inhibitors of *Lb*DHODH, bearing a moiety mimicking ORO connected to a covalent warhead, are promising leishmanicidal agents.

### Early ADME Profile

In order to evaluate the early ADME properties (Table 2 Supporting Information) of the BARB series, we selected compounds showing activity both in parasite and *Lb*DHODH (**2e** and **2i**) assays, active on the enzyme inhibition and inactive on the phenotypic assays (**2c** and **2d**), and inactive on the enzyme inhibition but active against the parasite (**2h**). Interestingly, most of the compounds showed good kinetic solubility, but at the same time some degree of chemical and/or metabolic instability. High intrinsic clearance (Cl_int_) values were reported in the mouse microsomes assay for all compounds except for **2e** (IC_50_ = 28 ± 3 μM, EC_50_ = 17 ± 11 μM). Additionally, high Cl_int_ values were obtained even in the absence of cofactors (NCF, SI2) and overall low recovery under the different ADME assay conditions, indicative of chemical instability. Particularly, compound **2d** (IC_50_ = 13 ± 2 μM, EC_50_ = 152 ± 28 μM) was shown to be very unstable under the assay matrices, and data for this compound should be carefully considered.

Compound **2h** (IC_50_ = >100, EC_50_ = 5 ± 2 μM) showed good passive permeability and the highest lipophilicity among the subset of compounds (cLogP 2.0), but was poorly soluble (4.63 μM) and metabolically unstable. This profile might explain the lack of *in vitro* potency seen for this compound. For **2i,** the most potent compound in the series, a similar profile of high permeability and poor chemical/metabolic stability was identified, but with a 10x higher solubility. Interestingly, the higher permeability of the most potent *Lb*DHODH inhibitor **2i** is coherent with its antiparasitic activity (IC_50_ = 0.5 ± 0.1 μM, EC_50_ = 10 ± 3.5 μM), while compound **2c**, which showed moderate potency against the enzyme (IC_50_ = 7 ± 1 μM) and was inactive in the phenotypic assay has the lowest permeability (8.4 nm/s).

This preliminary ADME data set helps further understand the *in vitro* potency data and clearly highlights the importance of considering physicochemical properties while investigating the compound’s biological activity. To balance potency with other properties such as permeability, solubility, and stability, is one of the goals to be pursued by rational modifications on the aryl ring to get more drug-like compounds.

The discrepancies between enzymatic and cellular potency reported in this study highlight the complexity of translating biochemical activity into antiparasitic effects. This disconnection occurs frequently in early stage drug discovery programs, particularly in target validation efforts for complex infectious agents. Future studies in this project will expand the derivative series, looking to improve compound physicochemical properties and potency (enzymatic inhibition and parasite growth inhibition) to enable more comprehensive structure–activity relationship analysis, while evolving to a lead optimization campaign. Furthermore, the essentiality of *Lb*DHODH for parasite survival requires additional validation, as alternative metabolic pathways or off-target effects may contribute to the observed cellular responses. While definitive target validation requires clinical evaluation, additional studies are needed to bridge the gap between enzymatic inhibition and cellular activity. These include target engagement assays in intact *Leishmania* spp., resistance selection experiments, and pharmacokinetic-pharmacodynamic characterization. Such studies will be necessary to determine the therapeutic potential of this target and optimize the translation from enzymatic inhibition to antiparasitic activity toward the identification of preclinical candidates.

## Conclusions

In this study, we designed and evaluated a series of barbituric acid derivatives as covalent inhibitors of *Lb*DHODH. Among them, compound **2i** demonstrated remarkable potency, with an IC_50_ of 0.5 ± 0.1 μM, significant antiparasitic activity against *Leishmania braziliensis* promastigote, and no cytotoxicity in mammalian cells. X-ray crystallography unambiguously confirmed the covalent binding mechanism of these inhibitors, highlighting the critical role of the exocyclic double bond in forming a covalent linkage with *Lb*DHODH. The structural analysis further revealed key interactions at the enzyme’s ORO-binding site, providing a solid foundation for the rational design of covalent inhibitors targeting this enzyme.

Our findings validate the hypothesis that the barbituric acid core mimics orotate binding while the covalent warhead establishes barbituric acid derivatives with an exocyclic double bond as a novel class of covalent inhibitors for *Lb*DHODH. This represents a promising avenue for the validation of new drug targets and ultimately generate candidates that could fulfill the urgent need for new treatments for neglected tropical diseases. The combination of **2i**’s potency, selectivity, and favorable cytotoxicity profile, together with the structural insights obtained in this study, underscores its potential for further optimization. Important next steps include structural refinements guided by the elucidated binding mode, additional chemical expansion and SAR studies to enhance both target and cellular potency (including testing against intracellular amastigotes) and assessment of the physicochemical and ADME properties of compounds to enable future *in vivo* studies.

Beyond advancing therapeutic strategies against parasitic infections, this work broadens the scope of covalent inhibitor development, demonstrating the potential of targeting dehydrogenases through covalent inhibition and offering broader implications for drug discovery in infectious diseases.

## Experimental Section

### Chemistry

Unless otherwise stated, all the solvents and reagents were obtained from commercial suppliers and used without prior purification. Chromatographic purification of the products was performed by flash column chromatography on silica gel (Sigma–Aldrich, particle size 0.040–0.063 nm) or using TELEDYNE ISCO CombiFlash Rf+ automatic chromatography column. Thin-layer chromatography (TLC) was carried out on silica plates (TLC Silica 60 F254 by Merck) and analyzed by UV light or by staining upon heating with vanillin or ninhydrin. NMR spectra were recorded on a Bruker Ultrashield 300-MHz NMR system (^1^H NMR: 300 MHz, ^13^C NMR: 75 MHz) or on a Bruker Ultrashield Avance 400-MHz NMR system (^1^H NMR: 400 MHz, ^13^C NMR: 101 MHz). Chemical shifts are referenced to residual solvent signals (DMSO-*d*_6_: 2.50 and 39.52 ppm for ^1^H NMR and ^13^C NMR, respectively), and reported in parts per million (ppm). Coupling constants (*J*) are reported in Hz, and multiplicities of NMR signals are abbreviated as follows: bs = broad singlet, s = singlet, d = doublet, dd= doublet of doublets, ddd = doublet of doublet of doublets, t = triplet, td = triplet of doublets, m = multiplet and combinations thereof, app = apparent. The purity of final compounds were determined via quantitative ^1^H NMR analysis 1,2,4,5-Tetrachloro-3-nitrobenzene and Maleic acid as an internal standard and achieved a minimum of 95%. Melting points were determined in open capillary tubes by using a BÜCHI Labortechnik M-560 melting point meter. The record of high-resolution masses (HRMS) was made via a Bruker Daltonics micrOTOF QII/ESI-TOF in positive mode.

### General Procedure 1

#### Pyrimidine-2,4,6­(1*H*,3*H*,5*H*)-trione (Barbituric Acid) Synthesis (**1**)

To a sealed tube (10 mL) was added malonic acid (1.04 g, 10 mmol, 1 equiv), urea (600.56 g, 10 mmol, 1 equiv) and acetic anhydride (∼2 mL, 20 mmol, 2 equiv). The reaction mixture was heated to 60 °C and kept under stirring for 3 h. The suspension was dried under reduced pressure. The solid residue was solubilized in hot ethanol (30 mL) and left to recrystallize at room temperature to furnish the product as a white solid (900 mg, 7 mmol, 70% yield).^45^ ^1^H NMR (300 MHz, DMSO-*d*_6_): δ 11.13 (s, 2H), 3.45 (s, 2H). ^13^C NMR (75 MHz, DMSO-*d*_6_) δ 168.3, 152.1, 45.5. HRMS (ESI-TOF) *m*/*z*: [M + H]^+^ calculated for C_4_H_5_N_2_O_3_: 129.0295; Found 129.0298. Data in accordance with the literature.

### General Procedure 2

To a round-bottom flask (25 mL) with barbituric acid (1.0 mmol, 1 eq., 0.1281 g) was added distilled water (10 mL) at 60 °C. To this mixture was added the desired aldehyde (1.2 mmol or1.2 equiv) in ethanol (2.0 mL). The reaction mixture was kept stirring at 60 °C during 16 h. The precipitate was filtered and washed with water and an abundant quantity of ethyl acetate to furnish the desired compounds **(2a-2f, 2h, 2i**).^45^

#### 5-Benzylidenepyrimidine-2,4,6­(1*H*,3*H*,5*H*)-trione (**2a**)

Benzaldehyde 1.2 eq was used in accordance to general procedure 2 as described above to give **2a** as a yellow solid (64 mg, 0:5 mmol, 50% yield. MP: 247.1–255.1 °C. 1H NMR (400 MHz, DMSO-d6) δ 11.42 (bs, 1H), 11.26 (bs, 1H), 8.29 (s, 1H), 8.08 (*dd*, *J* = 7.2, 1.8 Hz, 2H), 7.57–7.51 (m, 1H), 7.48 (m, 2H). 13C NMR (75 MHz, DMSO-d6) δ 163.9, 162.0, 155.2, 150.7, 133.6, 133.1, 132.7, 128.5, 119.6. HRMS (ESI-TOF) *m*/*z*: [M + H]+ calculated for C_11_H_9_N_2_O_3_: 217.0608; Found: 217.1062. Purity- 96.14%.

#### 4-((2,4,6-Trioxotetrahydropyrimidin-5­(2*H*)-ylidene)­methyl)­benzoic Acid (**2b**)

4-formylbenzoic acid 1.2 eq was used in accordance to general procedure 2 as described above to give **2b** as an orange solid in 80% yield. MP: 343.5–348.3 °C. 1H NMR (400 MHz, DMSO-d6) δ 13.28 (s, 1H), 11.47 (s, 1H), 11.30 (s, 1H), 8.31 (s, 1H), 8.05–7.94 (m, 4H). 13C NMR (75 MHz, DMSO-d6) δ 167.21, 163.45, 161.75, 153.32, 150.67, 137.57, 133.07, 132.46, 129.01, 121.39. HRMS (ESI-TOF) *m*/*z*: [M + H]+ calculated for C_12_H_8_N_2_O_5_: 261.0506; Found: 261.1408. Purity- 95.18%.

#### 5-(4-Hydroxy-3-methoxybenzylidene)­pyrimidine-2,4,6­(1*H*,3*H*,5*H*)-trione (**2c**)

4-hydroxy-3-methoxybenzaldehyde 1.2eq was used on accordance to general procedure 2 as described above to give **2c** as an orange solid (115 mg, 0.43 mmol, 70% yield). MP: 287.3–291.0 °C. 1H NMR (400 MHz, DMSO-d6) δ 11.29 (bs, 1H), 11.16 (bs, 1H), 11.16 (d, J = 2.0 Hz, 1H), 10.59 (s, 1H), 8.49 (d, J = 2.0 Hz, 1H), 8.23 (s, 1H), 7.81 (*dd, J* = 8.5, 2.1 Hz, 1H), 6.91 (d, J = 8.4 Hz, 1H), 3.83 (s, 3H). 13C NMR (75 MHz, DMSO-d6) δ 164.6, 162.9, 156.4, 153.5, 150.7, 147.4, 133.0, 124.6, 118.4, 115.8, 114.4, 55.9. HRMS (ESI-TOF) *m*/*z*: [M + H]+ calculated for C_12_H_11_N_2_O_5_: 263.0662; Found: 263.0590. Purity- 97.20%.

#### 5-(4-Hydroxy-3-nitrobenzylidene)­pyrimidine-2,4,6­(1*H*,3*H*,5*H*)-trione (**2d**)

4-hydroxy-3-nitrobenzaldehyde 1.2 eq was used in accordance to general procedure 2 as described above to give **2d** as an orange solid (113 mg, 0.40 mmol, 65% yield). MP: 258.1–260.1 °C. 1H NMR (400 MHz, DMSO-d6) δ 11.40 (bs, 1H), 11.29 (bs, 1H), 9.14 (d, *J* = 2.2 Hz, 1H), 8.28 (*dd, J* = 9.0, 2.3 Hz, 1H), 8.23 (s, 1H), 7.19 (d, *J* = 8.8 Hz, 1H). 13C NMR (75 MHz, DMSO-d6) δ 163.9, 162.6, 155.9, 153.0, 150.6, 141.6, 137.1, 131.8, 123.9, 118.9, 118.0. HRMS (ESI-TOF) *m*/*z*: [M + H]+ calculated for C_11_H_8_N_3_O_6_: 278.0408; Found:. Purity- 95.20%.

#### 5-(4-Morpholino-2-nitrobenzylidene)­pyrimidine-2,4,6­(1*H*,3*H*,5*H*)-trione (**2e**)

4-morpholino-2-nitrobenzaldehyde 1.2 eq was used in accordance to general procedure 2 as described above to give **2e** as a red solid (112 mg, 0.32 mmol, 41% yield). MP: 213.4–219.8 °C. 1H NMR (400 MHz, DMSO-d6) δ 11.41 (bs, 1H), 11.21 (bs, 1H), 8.37 (s, 1H), 7.73 (d, *J* = 8.9, 1H), 7.58 (d, *J* = 2.6 Hz, 1H), 7.29 (dd, *J* = 9.0, 2.7 Hz, 1H), 3.89–3.62 (m, 4H). 13C NMR (75 MHz, DMSO-d6) δ 163.5, 161.9, 152.8, 151.9, 150.7, 150.4, 134.4, 118.3, 117.9, 117.2, 108.4, 66.2, 47.2.

#### 5-([1,1′-Biphenyl]-4-ylmethylene)­pyrimidine-2,4,6­(1*H*,3*H*,5*H*)-trione (**2f**)

[1,1’-biphenyl]-4-carbaldehyde 1.2 eq was used in accordance to general procedure 2 as described above to give **2f** as an orange solid (113 mg, 0.40 mmol, 65% yield). MP: 258.1–260.1 °C. 1H NMR (300 MHz, DMSO-d6) δ 11.41 (s, 1H), 11.27 (s, 1H), 8.32 (s, 1H), 8.25 (d, *J* = 8.4 Hz, 2H), 7.84–7.75 (m, 4H), 7.56–7.40 (m, 3H). 13C NMR (75 MHz, DMSO-d6) δ 163.9, 162.2, 154.6, 150.7, 144.1, 139.3, 134.8, 132.2, 129.6, 128.9, 127.4, 126.6, 119.2. HRMS (ESI-TOF) *m*/*z*: [M + H]+ calculated for C_11_H_8_N_3_O_6_: 278.0408; Found:. Purity- 98.81%.

#### 5-((5-(2-Fluorophenyl)-1*H*-pyrrol-2-yl)­methylene)­pyrimidine-2,4,6­(1*H*,3*H*,5*H*)-trione (**2h**)

5-(2-fluorophenyl)-1H-pyrrole-2-carbaldehyde 1.2 eq was used in accordance to general procedure 2 as described above to give **2h** as a yellow solid (57 mg, 0.45 mmol, 45% yield). MP: 302.6–305.1 °C. 1H NMR (400 MHz, DMSO-d6) δ 12.51 (s, 1H), 11.13 (s, 1H), 11.02 (s, 1H), 8.45 (s, 1H), 8.32 (s, 1H), 7.77 (t, *J* = 7.8 Hz, 1H), 7.70 (s, 1H), 7.39–7.27 (m, 3H). 13C NMR (75 MHz, DMSO-d6) δ 164.9, 163.2, 150.9, 149.3, 136.1, 128.4, 127.3, 125.4, 121.3, 117.0, 116.7, 110.3. HRMS (ESI-TOF) *m*/*z*: [M + H]+ calculated for C_15_H_11_FN_3_O_3_: 300.0779; Found: 300.0779. Purity- 98.96%.

#### (*E*)-5-(3-Phenylallylidene)­pyrimidine-2,4,6­(1*H*,3*H*,5*H*)-trione (**2i**)

Cinnamaldehyde 1.2 eq was used in accordance to general procedure 2 as described above to give **2i** a yellow solid (159.5 mg, 0.66 mmol, 66%).4 MP: 242.9–250.2 °C. 1H NMR (300 MHz, DMSO-d6) δ 11.28 (s, 1H), 11.22 (s, 1H), 8.52–8.30 (m, 1H), 7.99 (d, *J* = 11.9 Hz, 1H), 7.71–7.61 (m, 3H), 7.45 (dd, *J* = 4.5, 2.3 Hz, 3H). 13C NMR (75 MHz, DMSO-d6) δ 163.6, 163.5, 154.3, 153.2, 150.8, 135.7, 131.7, 129.7, 129.1, 124.7, 116.1. HRMS (ESI-TOF) *m*/*z*: [M + H]+ calculated for C_13_H_11_N_2_O_3_: 243.0764; Found: 243.0781. Purity- 95.29%.

#### 5-(3-Hydroxybenzylidene)­pyrimidine-2,4,6­(1*H*,3*H*,5*H*)-trione (**2g**)

A reaction tube (10 mL) was charged with barbituric acid (1.1 mmol, 0.1409 g, 1.1 equiv), glacial acetic acid (3 mL, 0.33 M) and 3-hydroxybenzaldehyde (1.0 mmol, 0.1221 g, 1 equiv), and then sealed. The reaction mixture was slowly heated to 100 °C and left stirring for 2 h. After that, acetic acid was removed under reduced pressure and the residual solid was washed with abundant ethanol. The yellow solid was dried at 100 °C in oven and stored at room temperature (133.2 mg, 0.57 mmol, yield = 57%).4 MP: 245.0–249.6 °C (dec.). 1H NMR (300 MHz, DMSO-d6) δ 11.39 (d, *J* = 1.8 Hz, 1H), 11.23 (d, *J* = 1.8 Hz, 1H), 8.17 (s, 1H), 7.60 (d, *J* = 2.0 Hz, 1H), 7.43 (d, *J* = 7.7 Hz, 1H), 7.27 (t, *J* = 7.9 Hz, 1H), 6.94 (dd, *J* = 8.1, 2.5 Hz, 1H). 13C NMR (75 MHz, DMSO-d6) δ 164.0, 162.0, 157.3, 155.4, 150.7, 134.2, 129.6, 125.3, 120.1, 119.6, 119.3. HRMS (ESI-TOF) *m*/*z*: [M + H]+ calculated for C_11_H_8_N_2_O_4_: 233.0557; Found: 233.0270. Purity- 96.71%.

### General Procedure 3

To a round-bottom flask (10 mL), 5-benzilidenopyrimidine-2,4,6­(1*H*,3*H*,5*H*)-trione (50 mg, 0.23 mmol) was added and solubilized in ethanol (3 mL), and then sodium borohydride (17.40 mg, 0.46 mmol) was added with stirring for 15 min. After this time, the solvent was distilled under reduced pressure. To the solid residue was added water (2 mL), and the pH was adjusted to 2, by using a 0.1 M hydrochloric acid solution. The formed precipitate was filtered out, washed with water, and dried to obtain **3a-3c, 3e-3i.**

#### 5-Benzylpyrimidine-2,4,6­(1H,3H,5H)-trione (**3a**)

5-benzilidenopyrimidine-2,4,6­(1*H*,3*H*,5*H*)-trione **2a**(50 mg, 0.23 mmol) was reduced using general procedure 3 to furnish **3a** a white solid (0.05 g, 0.2291 mmol, 50%). MP: 210.6–213.1 °C. 1H NMR (400 MHz, DMSO-d6) δ 11.19 (s, 2H), 7.30–7.19 (m, 3H), 7.08 (d, *J* = 7.0 Hz, 2H), 3.90 (t, *J* = 4.9 Hz, 1H), 3.25 (d, *J* = 4.8 Hz, 2H). 13C NMR (75 MHz, DMSO-d6) δ 170.4, 151.0, 137.9, 129.3, 128.8, 127.2, 49.8, 33.8. HRMS (ESI-TOF) *m*/*z*: [M + Na]+ calculated for C_11_H_10_N_2_NaO_3_: 241.0584; Found: 241.0584.

#### 4-((2,4,6-Trioxohexahydropyrimidin-5-yl)­methyl)­benzoic Acid (**3b**)

**4**-((2,4,6-trioxotetrahydropyrimidin-5­(2*H*)-ylidene)­methyl)­benzoic acid **2b** was reduced using general procedure 3 to obtain **3b** as a white solid (0.046 g, 0.1754 mmol, 93%). MP: 283.8–288.1 °C. 1H NMR (400 MHz, DMSO-d6) δ 12.89 (s, 1H), 11.23 (s, 2H), 7.83 (d, *J* = 7.9 Hz, 2H), 7.23 (d, *J* = 7.8 Hz, 2H), 4.07–3.96 (t, 1H), 3.33–3.27 (d, 2H). HRMS (ESI-TOF) *m*/*z*: [M+K]+ Calculated for C_12_H_10_KN_2_O_5_: 301.0221; Found: 301.0250.

#### 5-(4-Hydroxy-3-methoxybenzyl)­pyrimidine-2,4,6­(1*H*,3*H*,5*H*)-trione (**3c**)

5-(4-hydroxy-3-methoxybenzylidene)­pyrimidine-2,4,6­(1*H*,3*H*,5*H*)-trione **2c** was reduced using general procedure 3 to obtain **3c** as a beige solid (0.010 g, 0.037 mmol, 51%). MP: 193.6–197.1 °C. 1H NMR (400 MHz, DMSO-d6) δ 11.17 (s, 2H), 9.02 (s, 1H), 6.67 (d, *J* = 8.0 Hz, 1H), 6.59 (s, 1H), 6.43 (d, *J* = 8.5 Hz, 1H), 3.76 (t, *J* = 4.4 Hz, 1H), 3.68 (s, 3H), 3.15 (d, *J* = 7.0 Hz, 2H). 13C NMR (75 MHz, DMSO-d6) δ 170.4, 151.0, 137.9, 129.3, 128.8, 127.2, 73.9, 49.8, 33.7. HRMS (ESI-TOF) *m*/*z*: [M+2Na–H]+ Calculated for C_12_H_11_N_2_Na_2_O_5_: 309.0458; Found: 309.0448.

#### 5-(4-Morpholino-2-nitrobenzyl)­pyrimidine-2,4,6­(1*H*,3*H*,5*H*)-trione (**3e**)

5-(4-morpholino-2-nitrobenzylidene)­pyrimidine-2,4,6­(1*H*,3*H*,5*H*)-trione **2e** was reduced using general procedure 3 to obtain **3e** as a yellow solid (0.045 g, 0.1291 mmol, 90%). MP: 193.6–197.1 °C. FTIR (cm-1): 3519, 2977, 2862, 1739, 1625, 1537, 1358, 1240, 1121, 968, 883. 1H NMR (400 MHz, DMSO-d6) δ 11.19 (s, 2H), 10.80 (s, 1H), 7.39 (d, J = 2.7 Hz, 1H), 7.23 (s, 2H), 7.03 (t, *J* = 8.7 Hz, 1H), 3.92 (t, *J* = 6.1 Hz, 1H), 3.73 (m, 6H), 3.21–3.15 (m, 4H). 13C NMR (75 MHz, DMSO) δ 170.1, 151.3, 132.7, 130.4, 125.3, 122.4, 120.0, 119.4, 109.7, 87.0, 66.3, 49.4, 48.4, 48.0, 27.9. HRMS (ESI-TOF) *m*/*z*: [M + Na]+ Calculated for C_15_H_16_N_4_NaO_6_: 371.0962; Found: 371.0958.

#### 5-([1,1′-Biphenyl]-4-ylmethyl)­pyrimidine-2,4,6­(1*H*,3*H*,5*H*)-trione (**3f**)

5-([1,1’-biphenyl]-4-ylmethylene)­pyrimidine-2,4,6­(1*H*,3*H*,5*H*)-trione **2f** was reduced using general procedure 3 to obtain **3f** as a yellow solid (0.075 g, 0.257 mmol, 75%). MP: 269.8–273.2 °C. 1H NMR (400 MHz, DMSO-*d*6) δ 11.24 (s, 2H), 7.64 (d, *J* = 7.7 Hz, 2H), 7.57 (d, *J* = 7.9 Hz, 2H), 7.44 (t, *J* = 7.5 Hz, 2H), 7.34 (t, *J* = 7.4 Hz, 1H), 7.18 (d, *J* = 7.9 Hz, 2H), 3.97 (t, *J* = 4.9 Hz, 1H), 3.30 (d, *J* = 5.0 Hz, 2H).

#### 5-(3-Hydroxybenzyl)­pyrimidine-2,4,6­(1*H*,3*H*,5*H*)-trione (**3g**)

5-(3–5-(3-hydroxybenzylidene)­pyrimidine-2,4,6­(1*H*,3*H*,5*H*)-trione **2g** was reduced using the general procedure 3 to obtain as **3g** as a green solid (0.035g, 0.1494 mmol, 70%). MP: 194.1–197.1 °C. 1H NMR (300 MHz, DMSO-d6) δ 11.18 (s, 2H), 9.33 (s, 1H), 7.02 (t, *J* = 8.0 Hz, 1H), 6.54 (d, *J* = 24.0 Hz, 3H), 3.91–3.77 (m, 1H), 3.24–3.09 (m, 2H). 13C NMR (75 MHz, DMSO-d6) δ 172.0, 157.6, 151.0, 129.6, 119.8, 116.1, 113.7, 75.5, 49.6, 39.8, 33.6. HRMS (ESI-TOF) *m*/*z*: [M+ H]+ Calculated for C_11_H_11_N_2_O_4_: 235.0713; Found: 235.0724. Purity- 98.38%.

#### 5-((5-(2-Fluorophenyl)-1*H*-pyrrol-2-yl)­methyl)­pyrimidine-2,4,6­(1*H*,3*H*,5*H*)-trione (**3h**)

55-((5-(2-fluorophenyl)-1H-pyrrol-2-yl)­methylene)­pyrimidine-2,4,6­(1*H*,3*H*,5*H*)-trione **2h** was reduced using general procedure 3 to obtain **3h** as a purple-pink solid (0.036 g, 0.1194 mmol,73%). MP: 205.6–209.1 °C. FTIR (cm-1): 3483, 3243, 3100, 2851, 1729, 1443, 1363, 1217, 805, 757, 513. 1H NMR (400 MHz, DMSO-d6) δ 11.19 (s, 2H), 11.09 (s, 1H), 7.64 (dd, *J* = 8.5, 5.7, 3.1 Hz, 1H), 7.20 (tt, *J* = 6.1, 3.7 Hz, 3H), 6.57 (s, 1H), 6.29 (s, 1H), 3.73 (t, *J* = 4.4 Hz, 1H), 3.13 (d, *J* = 4.3 Hz, 2H). 13C NMR (75 MHz, DMSO-d6) δ 170.9, 160.1, 156.6, 151.1, 127.4, 126.3, 125.1, 120.8, 119.3, 118.9, 116.7, 116.4, 110.3, 49.8, 26.4. HRMS (ESI-TOF) *m*/*z*: [M+2Na–H]+ Calculated for C_15_H_11_FN_3_Na_2_O_3_: 346.0574; Found: 346.0575.

#### 5-Cinnamylpyrimidine-2,4,6­(1*H*,3*H*,5*H*)-trione (**3i**)

(*E*)-5-(3-Phenylallylidene)­pyrimidine-2,4,6­(1*H*,3*H*,5*H*)-trione **2i** using general procedure 3 to obtain **3i** as a white solid (0.028 g, 0.1146 mmol, 56%). MP: 209.6–213.1 °C. 1H NMR (400 MHz, DMSO-d6) δ 11.26 (s, 2H), 7.37–7.27 (m, 4H), 7.22 (t, *J* = 7.1 Hz, 1H), 6.44 (d, *J* = 15.8 Hz, 1H), 6.20–6.09 (m, 1H), 3.76 (t, *J* = 5.1 Hz, 1H), 2.82 (t, *J* = 6.0 Hz, 2H). 13C NMR (75 MHz, DMSO-d6) δ 170.3, 151.3, 137.0, 133.0, 129.0, 127.9, 126.5, 125.5, 48.6, 31.5. HRMS (ESI-TOF) *m*/*z*: [M + Na]+ Calculated for C_13_H_12_N_2_NaO_3_: 267.0740; Found: 267.0728.

#### 5-(3-Phenylpropyl)­pyrimidine-2,4,6­(1*H*,3*H*,5*H*)-trione (**3ii**)

(*E*)-5-(3-Phenylallylidene)­pyrimidine-2,4,6­(1*H*,3*H*,5*H*)-trione **2i** was suspended in 30 mL of methanol (100 mg, 2 mmol) and 5% Pd/C (10 mg) was stirred under hydrogen atmosphere at room temperature for 5 h. The catalyst was separated by filtration through Celite and was evaporated to afford **3ii** a white (96%). MP: 193.6–197.1 °C 1H NMR (400 MHz, DMSO-d6) δ 11.21 (s, 2H), 7.29–7.23 (m, 2H), 7.20–7.14 (m, 3H), 3.55 (t, *J* = 5.3 Hz, 1H), 2.55 (t, *J* = 7.6 Hz, 2H), 1.90 (p, *J* = 5.4, 5.0 Hz, 2H), 1.63–1.43 (m, 2H). 13C NMR (75 MHz, DMSO-d6) δ 172.1, 150.0, 141.5, 128.4, 128.3, 125.9, 74.7, 38.7, 34.6, 24.8.

### Protein Expression and Purification

The expression and purification of the *Lb*DHODH were carried out as previously described by Reis and co-workers.^32^ Briefly, *E. coli* BL21­(DE3) strain carrying pET-28a­(+)-*Lb*DHODH was grown at 37 °C (180 rpm) in in LB broth supplemented with 30 μg/mL until OD600 reached 0.6- 0.8. Then, 0.5 mM isopropyl-d-1-thiogalactopyranoside (IPTG) was added to the culture, and the temperature was reduced to 25 °C. After 5 h, the cells were harvested by centrifugation (Sorvall RC6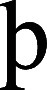 - Thermo Scientific), at 4 °C, 30 min, and then lysed by sonication in a lysis buffer consisting of 50 mM sodium phosphate, 300 mM NaCl (pH 8.0) and 1 mM phenylmethylsulfonyl-fluoride. The soluble fraction was separated by centrifugation at 16 000g for 30 min at 4 °C and then loaded onto Ni–NTA agarose affinity resin (Invitrogen) equilibrated with a lysis buffer. The column was washed with a lysis buffer supplemented with a step gradient of 0, 25, 50, and 250 mM imidazole. The protein fractions were pooled and dialyzed to 50 mM HEPES, 150 mM NaCl (pH 7.2). All purification steps followed by SDS-PAGE 14%, and the final protein concentration was estimated based on its molar extinction coefficient (11. 352 M^–1^cm^–1^ at 459 nm).

### Crystallization and Data Collection of *Lb*DHODH

For cocrystallization experiments the protein was dialyzed in 50 mM HEPES, 150 mM NaCl (pH 7.2), 0.1 mM FMN. *Lb*DHODH in complex with inhibitors were crystallized by sitting drop vapor diffusion method, following a 24 h incubation of 8 mg/mL *Lb*DHODH with each inhibitor (5 mM) at 21 °C. Prior to crystallization, the sample was clarified through centrifugation. The drop contained 1.0 μL of protein solution and 1 μL of reservoir solution. Crystals of *Lb*DHODH were obtained in the presence of 0.6–1.6 M ammonium sulfate, 0.25–1 M lithium sulfate, 0.1 M sodium citrate (pH 5.6). The *Lb*DHODH crystals were soaked in a cryoprotectant solution (0.6–1.6 M ammonium sulfate, 0.25–1 M lithium sulfate, 0.1 M sodium citrate (pH 5.6), 20% v/v glycerol), before cooling in liquid nitrogen. Data collection was performed in two beamlines. The PROXIMA 2 protein crystallography beamline at SOLEIL, France, at 100 K using an EIGER-X 9 M detector (Dectris, Baden, Switzerland). 3600 frames were collected using an exposure time of 0.1 s per image, with an oscillation step of 0.1°. The diffraction data were processed using XDSAPP3. Integration and scaling were done using XDS. The data were evaluated for radiation damage using XDSSTAT, pseudotranslational symmetry was assessed using SFCHECK and POINTLESS confirmed the absence of twinning. The resulting intensity file was then converted to structure factor amplitudes in mtz format using XDSCONV and f2mtz. In the Manaca protein crystallography beamline at SIRIUS, Brazil, at 100 K using a PILATUS 2 M detector (Dectris). 3600 frames were collected using an exposure time of 0.1 s per image, with an oscillation step of 0.1°. The images of X-ray diffraction were processed with the XDS package.

### Crystal Structure Determination and Refinement

The structure was solved by molecular replacement techniques implemented in PHASER-MR (27) with the *Lb*DHODH apo structure (PDB ID: 4WZH)^32^ as a search model. Model rebuilding and refinement were performed using Coot^46^ and phenix.refine,^47^ respectively. The quality of the model was regularly checked using MolProbity.^48^ Diffraction data and refinement statistics are summarized in Table 1S. All structural figures were produced using PyMOL (Schrödinger; http://www.pymol.org). The refined atomic coordinates and structure factors were deposited in the PDB with the accession code 9N67, 9CB8, 9N6O, 9N68, 9N6Q.

### Inhibition Assay

The experiments were performed in a 96-well microplate reader (SpectraMax Plus 384, Molecular Devices, USA), containing the 190 μM of the buffer 60 mM DCIP, 50 mM Tris pH 8.15, 150 mM KCl, 0.1% Triton X-100, 50 μM DHO and 10 μM of *Lb*DHODH at 165 nM. The reaction was monitored every 3 s at 610 nm over a period of 60 s, in triplicate, for the control and each concentration of the inhibitor. All assays were performed without incubation (0h) and with 4-h preincubation of the inhibitor with *Lb*DHODH, using 5% DMSO (v/v) as control. The initial screening was performed with 100 μM of compounds, and those that inhibited 50% or more of the enzyme activity followed to determine the IC_50_ values, a series of 12-point, 1:1 serial dilutions was performed from a highest starting concentration of 500 μM. To determine the IC_50_ values, the activity percentage data were fitted as a function of the concentration logarithm by nonlinear regression analysis (variable hillslope) using Prism 9.0 software (GraphPad Software, USA).^49^ To measure k*inact* and K*I*, complete enzymatic activity progress curves were generated using the same conditions described and the reaction was monitored every 3 s at 610 nm over a period of 30 min. These data were fit using nonlinear regression to a one-phase decay model^41^ to the equation P = (Vi/kobs)*­(1-e–kobs*t) to extract kobs values. The resulting k*obs* values were plotted as a function of inhibitor concentration, and the data in the initial linear region was fit to determine the slope, which is k*inact*/K*I*. All regressions were performed with GraphPad Prism 9.^49^

### Selectivity Assay

Human DHODH expression and purification were conducted following a protocol previously established in our laboratory.^50^ The purification was carried out by affinity chromatography on a Ni-NTA Agarose column (Qiagen□ Cat. No. 30210). For enzymatic assays, the purity of the protein was assessed by SDS-PAGE gel, and the protein was diluted to a final concentration of 800 nM in Buffer A based on the molar extinction coefficient ε454 nm of 14.26 mM^–1^ cm^–1^.Inhibitory assays were conducted by indirectly measuring enzymatic activity, monitoring the reduction of 2,6-dichlorophenolindophenol (DCIP), according to a previously established protocol.^42^ The reaction buffer containing 60 μM DCIP, 50 mM Tris pH 8.15, 150 mM KCl, 0.1% Triton X-100, 500 μM DHO, 100 μM Coenzyme-Q0, 2.5% DMSO, and 100 μM compound was used to assess inhibition. The reaction was performed in triplicate, initiated by adding 195 μL of reaction buffer to 5 μL of *Hs*DHODH protein solution for a final concentration of 20 nM enzyme. As a blank, 195 μL of reaction buffer was added to 5 μL of Buffer A. The reaction was monitored at 610 nm every 3 s over 60 s. As a control, 5 μL of enzyme was added to 195 μL of reaction medium without compound. Enzymatic velocity was calculated, and the percentage of relative enzymatic activity was determined relative to the control.

### Inhibition Assay

#### Sensitivity Tests to DHODH Inhibitors

Promastigotes of *Leishmania (V.) braziliensis* (MHOM/BR/1975/M2904) were grown and maintained in M199 medium (Thermo Fischer Scientific, #31100–027) supplemented with 10% dialyzed fetal bovine serum and 1 μM of biopterin. The cultures were adjusted to 4 × 10^6^ parasites per mL, and 50 μL of each culture was added on a test plate with 384 wells (Corning, #CLS3658), along with the controls and compounds to be tested. Previously diluted compounds in DMSO were added to plates in decreasing concentrations, with a maximum concentration of 200 μM. Parasites were exposed to a final concentration of DMSO in various dilutes (final = 0.5%), and the culture medium was used as the control for the experiment. After 72 h of incubation in the presence of the compounds, 15 μL of a lysis solution containing Tris-HCl 30 mM, pH 7.4, EDTA 7.5 mM, 0.012% saponin, 0.6% Triton X-100, and SYBR Green (Thermo Fischer Scientific, #S-7563) in a 1:2000 dilution was added to each well. After adding the lysis buffer, the plates were incubated in the dark for 2 h with gentle shaking. The readings were done using a SpectraMax M3 reader (excitation at 485 nm and emission at 535 nm). The half maximal effective concentration (EC_50_) and confidence intervals at 95% were calculated in triplicate measurements by nonlinear fitting (four parameters) using Prism 10 (GraphPad).

### Early ADME Profile

#### Parallel Artificial Membrane Permeability Assay (PAMPA)

To determine the passive permeability of the compounds, a 96-well plate containing precoated membranes was used (Corning Gentest # 353015). Solutions of the compounds were prepared in duplicate by diluting the stock solutions (10 mM) in phosphate buffered saline (PBS) pH 7.4 at a final concentration of 10 μM (5% DMSO). The solutions diluted in PBS pH 7.4 were then added to the donor portion of the plate (300 μL/well), while in the acceptor portion PBS pH 7.4 (200 μL/well) was added. The two portions of the plate were then coupled, and the system was incubated for 6 h at room temperature. Samples of the initial donor solution (T0) were collected at the beginning (T0) and stored at 5 °C. The final concentrations of compounds in the donor, acceptor and T0 wells were quantified by LC-MS/MS.

#### Microsomal Stability

For the experimental stability determination of test compounds in mouse liver microsomes in the presence of NADPH a clearance rate is determined. Test system: 0.4 mg/mL liver microsomal mouse protein, 1 μM test compound, at pH 7.4 and 37 °C. Samples were taken at 0, 5, 10, 20, 30, and 60 min in duplicate. Time point samples were analyzed by LC/MS/MS.

#### Kinetic Solubility Assay

To determine kinetic solubility, samples of each compound were transferred to a 96-well plate (incubation plate) in duplicate and 0.01 M PBS was added until a final concentration of 200 μM was reached (1% DMSO). The plate was incubated for 2 h at 25 °C. The precipitates on the incubation plate were removed by filtration and the filtrates were quantified by LC-MS/MS. Calibration curves were prepared for each compound by using 5–6 points. Samples were analyzed by UV–vis.

### Antiparasitic Activity

#### Cell Viability Assays

THP-1 cells (American Type Cell Collection, #TIB-202) were acquired in 2019 and stored frozen in liquid nitrogen. The cells were then maintained in in RPMI 1640 (Thermo Fisher Scientific, #S31800–022) at 37 °C in a 5% CO_2_ atmosphere and routinely tested for *Mycoplasma* contamination by PCR using the primers 5′-GGCGAATGGGTGAGTAACACG and 5′-CGGATAACGCTTGCGACCTAT. To test the toxicity of compounds, 5000 cells were added to 96-well plates with a final volume of 80 μL centrifuged at 400*g* for 2 min and incubated with 150 ng/mL of phorbol myristate acetate (Merck, #P8139) for 48 h. The medium was replaced, and after 24 h, the compounds were added and tested at decreasing concentrations starting from 200 μM (10 μL/well). After 72 h of incubation, 10 μL of PrestoBlue Cell Viability Reagent (Thermo Fisher Scientific, #A13262) was added per well. After adding the reagent, the cells were incubated for 2 h at 37 °C in a 5% CO_2_ atmosphere, and fluorescence readings were performed using the SpectraMax M3 reader (excitation at 560 nm and emission at 590 nm). The 50% toxicity levels (CC_50_) and confidence intervals at 95% were calculated in triplicate measurements by nonlinear fitting (four parameters) using Prism 10 (GraphPad).

### Theoretical Studies

#### Density Functional Theory Studies

Density functional theory (DFT) calculations were performed using the Amsterdam Density Functional (ADF) software package. Initially, the compounds were drawn using AMSjobs, followed by preliminary geometry optimization in AMSinput. Subsequently, the ground-state geometry of representative barbituric derivatives was optimized at the B3LYP/TZ2P level of theory. The HOMO and LUMO orbitals were analyzed, focusing on the occupied and the lowest unoccupied molecular orbitals (MOs), which are critical for understanding the intrinsic reactivity of the compounds.

## Supplementary Material







## Notes

Data collection and processing; results of the single dose assays for HsDHODH and compound characterization data; ^1^H and ^13^C NMR for all compounds and HPLC spectra for **2i**; data from the inhibition assay, DFT calculations, and PDB validation report (PDF)
Raw supporting information with pharmacokinetic data (XLS)
Molecular formula strings (CSV)

## Abbreviations

ADF: Amsterdam density functional
BARB: barbituric acid ring
CC50: cytotoxic concentration 50%
CL: cutaneous leishmaniasis
CNPEM: Centro Nacional de Pesquisa em Energia e Materiais (Brazilian Center for Research in Energy and Materials)
Coot: crystallographic object-oriented toolkit
Da: Dalton
DCIP: 2,6-dichlorophenolindophenol
DCM: dichloromethane
DFT: density functional theory
DHO: dihydroorotate
DNDi: Drugs for Neglected Diseases initiative
DHODH: dihydroorotate dehydrogenase
DMSO: dimethyl sulfoxide
EC50: half-maximal effective concentration
EDTA: ethylenediaminetetraacetic acid
EDG: electron-donating groups
EWG: electron-withdrawing groups
FMN: flavin mononucleotide
FMNH_2_: reduced flavin mononucleotide
GP: general procedure
HsDHODH: human dihydroorotate dehydrogenase
HOMO: highest occupied molecular orbital
HRMS: high-resolution mass spectrometry
HTS: high-throughput screening
IC50: half-maximal inhibitory concentration
IPTG: isopropyl-β-d-thiogalactopyranoside
J: coupling constant (NMR)
KI: inhibitory constant
Kinact: rate constant of inactivation
LbDHODH: *Leishmania braziliensis* dihydroorotate dehydrogenase
LC-MS/MS: liquid chromatography-tandem mass spectrometry
LUMO: lowest unoccupied molecular orbital
NMR: nuclear magnetic resonance
Ni-NTA: nickel-nitrilotriacetic acid
ORO: orotate
PDB: Protein Data Bank
ppm: parts per million
RMSD: root-mean-square deviation
SAR: structure–activity relationship
SDS-PAGE: sodium dodecyl sulfate-polyacrylamide gel electrophoresis
THF: tetrahydrofuran
TLC: thin layer chromatography
WHO: World Health Organization
XDS: X-ray detector software
