# Supplementary material for: Barbituric Acid Derivatives as Covalent Inhibitors of Leishmania braziliensis Dihydroorotate Dehydrogenase
Source: J Med Chem. 2025 Jul 14;68(18):18869–84. doi: 10.1021/acs.jmedchem.5c00462 (PMC12481576; doi:10.1021/acs.jmedchem.5c00462)
Supplement: Supplementary file 1 [file jm5c00462_si_001.pdf]

Barbituric acid derivatives as covalent inhibitors of *Leishmania braziliensis* dihydroorotate dehydrogenase

SUPPORTING INFORMATION

Thamires Quadros Froes [†‡], Temitayo Omowumi Alegbejo Price [†#], Bruna Fleck Godoi [†#], Miguel de Menezes Vaidergorn[†‡], Thiago dos Santos[†], Pedro Ivo Palacio Leite[†], Daniel Gedder Silva[†], Aline Dias da Purificação[†‡], Leonardo Loch[§], Sergio Schenkman[§], Jadel M. Kratz[&], Flavio da Silva Emery[†], Maria Cristina Nonato[†‡]**

[†] Center for the Research and Advancement in Fragments and molecular Targets (CRAFT), School of Pharmaceutical Sciences at Ribeirao Preto, University of São Paulo, Ribeirão Preto 14040-903, SP, Brazil

[‡] Protein Crystallography Laboratory, Department of Biomolecular Sciences, School of Pharmaceutical Sciences of Ribeirao Preto, University of São Paulo, Ribeirão Preto 14040-903, SP, Brazil

[§] Universidade Federal de São Paulo, Departamento de Microbiologia Imunobiologia e Parasitologia, Disciplina de Biologia Celular. R. Pedro de Toledo 669 6A, Vila Clementino, 04039032 - São Paulo, SP – Brasil

[&] Drugs for Neglected Diseases initiative (DNDi) Latin America, Rio de Janeiro 20010-020, SP, Brazil;

Corresponding authors: *Maria Cristina Nonato, cristy@fcfrp.usp.br, orcid: 0000-0002-4916-1505; *Flavio da Silva Emery, flavioemery@usp.br, orcid: 0000-0002-8652-7123; Center for the Research and Advancement in Fragments and Molecular Targets (CRAFT), School of Pharmaceutical Sciences at Ribeirao Preto, University of São Paulo, Ribeirão Preto 14040-903, SP, Brazil.

Table of Contents

Figure 1S: The sequences of *Lb*DHODH and *Hs*DHODH are grouped by green and pink, respectively. The residues conserved between both classes are shown in bold, and residues of the active site are highlighted in a yellow square. On top and on the bottom of the sequences are shown the secondary structures of *Lb*DHODH and *Hs*DHODH, respectively. Structural alignment was performed with the MULTALIN tool[1] and graphically displayed using ESPript[2]. 5

Figure 2S: Superposition of the *Lb*DHODH (PDB ID: 4WZH) and *Hs*DHODH (PDB ID: 2PRM) structures. *Lb*DHODH is shown in green and forms a dimer, while *Hs*DHODH, shown in light pink, is a monomer. The main structural differences are found in the additional N-terminal helical domain (dark pink) of the human enzyme. This domain, characteristic of class 2 DHODHs, is responsible for membrane association and forms a tunnel leading to the active site. 6

Table 1S: Data collection and processing 7

Table 2S. Results of the Single Dose Assays of 2a-i and 3a-3ii against *Hs*DHODH 9

Figure 3S. IC50 determination without (0h) and with (4h) incubation for barbituric acid derivatives. 10

Figure 4S. K*inact*/K*I* determination for 2a, 2d, 2g and 2i 11

1H NMR (400MHz, DMSO-d6) of pyrimidine-2,4,6(1H,3H,5H)-trione (1) 12

13C NMR (75MHz, DMSO-d6) of pyrimidine-2,4,6(1H,3H,5H)-trione (1) 13

1H NMR (400MHz, DMSO-d6) of 5-benzylidenepyrimidine-2,4,6(1H,3H,5H)-trione (2a) 14

^{1}H NMR (400MHz, DMSO-d_6) of 4-((2,4,6-trioxotetrahydropyrimidin-5(2H)-ylidene)methyl)benzoic acid (2b) 15

^{1}H NMR (400MHz, DMSO-d_6) of 5-(4-hydroxy-3-methoxybenzylidene)pyrimidine-2,4,6(1H,3H,5H)-trione (2c) 16

^{1}H NMR (400MHz, DMSO-d_6) of 5-(4-hydroxy-3-nitrobenzylidene)pyrimidine-2,4,6(1H,3H,5H)-trione (2d) 17

1H NMR (400MHz, DMSO-d6) of 5-(4-morpholino-2-nitrobenzylidene)pyrimidine-2,4,6(1H,3H,5H)-trione (2e) 18

^{1}H NMR (400MHz, DMSO-d_6) of 5-([1,1'-biphenyl]-4-ylmethylene)pyrimidine-2,4,6(1H,3H,5H)-trione (2f) 19

^{1}H NMR (400MHz, DMSO-d_6) of 5-(3-hydroxybenzylidene)pyrimidine-2,4,6(1H,3H,5H)-trione (2g) 20

^{1}H NMR (400MHz, DMSO-d_6) of 5-((5-(2-fluorophenyl)-1H-pyrrol-2-yl)methylene)pyrimidine-2,4,6(1H,3H,5H)-trione (2h) 21

^{1}H NMR (400MHz, DMSO-d$_6$) of (E)-5-(3-phenylallylidene)pyrimidine-2,4,6(1H,3H,5H)-trione (2i) 22

^{13}C NMR (75 MHz, DMSO-d$_6$) of (E)-5-(3-phenylallylidene)pyrimidine-2,4,6(1H,3H,5H)-trione (2i) 23

^{1}H NMR (400MHz, DMSO-d$_6$) of 5-benzylpyrimidine-2,4,6(1*H*,3*H*,5*H*)-trione (3a): 24

^{1}H NMR (400MHz, DMSO-d$_6$) of 4-((2,4,6-trioxohexahydropyrimidin-5-yl)methyl)benzoic acid (3b) 25

FTIR spectra of 4-((2,4,6-trioxohexahydropyrimidin-5-yl)methyl)benzoic acid (3b) 26

^{1}H NMR (400MHz, DMSO-d$_6$) of 5-(4-hydroxy-3-methoxybenzyl)pyrimidine-2,4,6(1H,3H,5H)-trione (3c) 27

^{1}H NMR (400MHz, DMSO-d$_6$) of 5-(4-morpholino-2-nitrobenzyl)pyrimidine-2,4,6(1H,3H,5H)-trione (3e) 28

FTIR of 5-(4-morpholino-2-nitrobenzyl)pyrimidine-2,4,6(1H,3H,5H)-trione (3e) 29

^{1}H NMR (400MHz, DMSO-d$_6$) of 5-([1,1'-biphenyl]-4-ylmethyl)pyrimidine-2,4,6(1H,3H,5H)-trione (3f) 30

^{1}H NMR (400MHz, DMSO-d$_6$) of 5-(3-hydroxybenzyl)pyrimidine-2,4,6(1H,3H,5H)-trione (3g) 31

^{1}H NMR (400MHz, DMSO-d$_6$) of 5-((5-(2-fluorophenyl)-1H-pyrrol-2-yl)methyl)pyrimidine-2,4,6(1H,3H,5H)-trione (3h) 32

FTIR of 5-((5-(2-fluorophenyl)-1H-pyrrol-2-yl)methyl)pyrimidine-2,4,6(1H,3H,5H)-trione (3h) 33

^{1}H NMR (400MHz, DMSO-d$_6$) of 5-(3-phenylpropyl)pyrimidine-2,4,6(1*H*,3*H*,5*H*)-trione (3i). 34

^{1}H NMR (400MHz, DMSO-d$_6$) of 5-cinnamylpyrimidine-2,4,6(1H,3H,5H)-trione (3ii) 35

Purity of compounds qNMR 35

Table 3S. Determination of the purity of tested compounds using quantitative NMR (absolute internal calibrant method). 37

HPLC data of compound 2i 48

Figure 5S: The relative energies of the frontier molecular orbitals of barbituric derivatives: (a) 2d and (b) 2g. The HOMO orbitals are highlighted in blue and red, while the LUMO orbitals are shown in green and cyan. 49

Figure 1S: The sequences of *Lb*DHODH and *Hs*DHODH are grouped by green and pink, respectively. The residues conserved between both classes are shown in bold, and residues of the active site are highlighted in a yellow square. On top and on the bottom of the sequences are shown the secondary structures of *Lb*DHODH and *Hs*DHODH, respectively. Structural alignment was performed with the MULTALIN tool[1] and graphically displayed using ESPript[2].

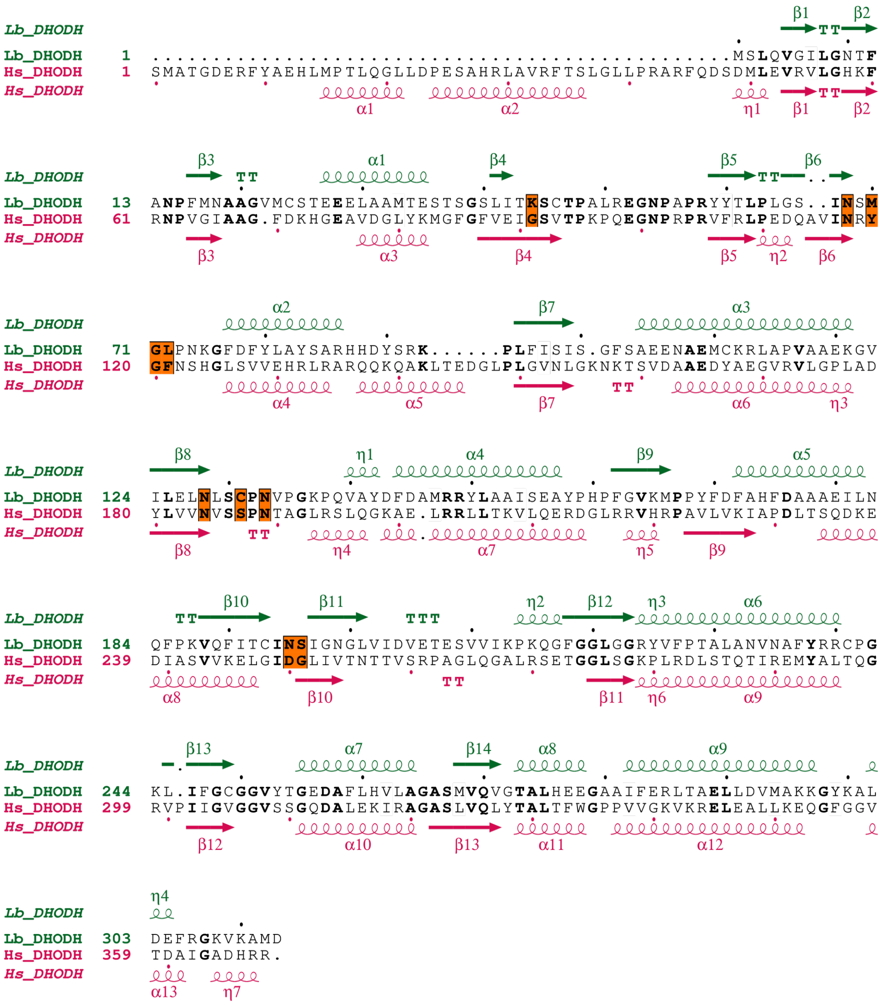

Figure 2S: Superposition of the *Lb*DHODH (PDB ID: 4WZH) and *Hs*DHODH (PDB ID: 2PRM) structures. *Lb*DHODH is shown in green and forms a dimer, while *Hs*DHODH, shown in light pink, is a monomer. The main structural differences are found in the additional N-terminal helical domain (dark pink) of the human enzyme. This domain, characteristic of class 2 DHODHs, is responsible for membrane association and forms a tunnel leading to the active site.

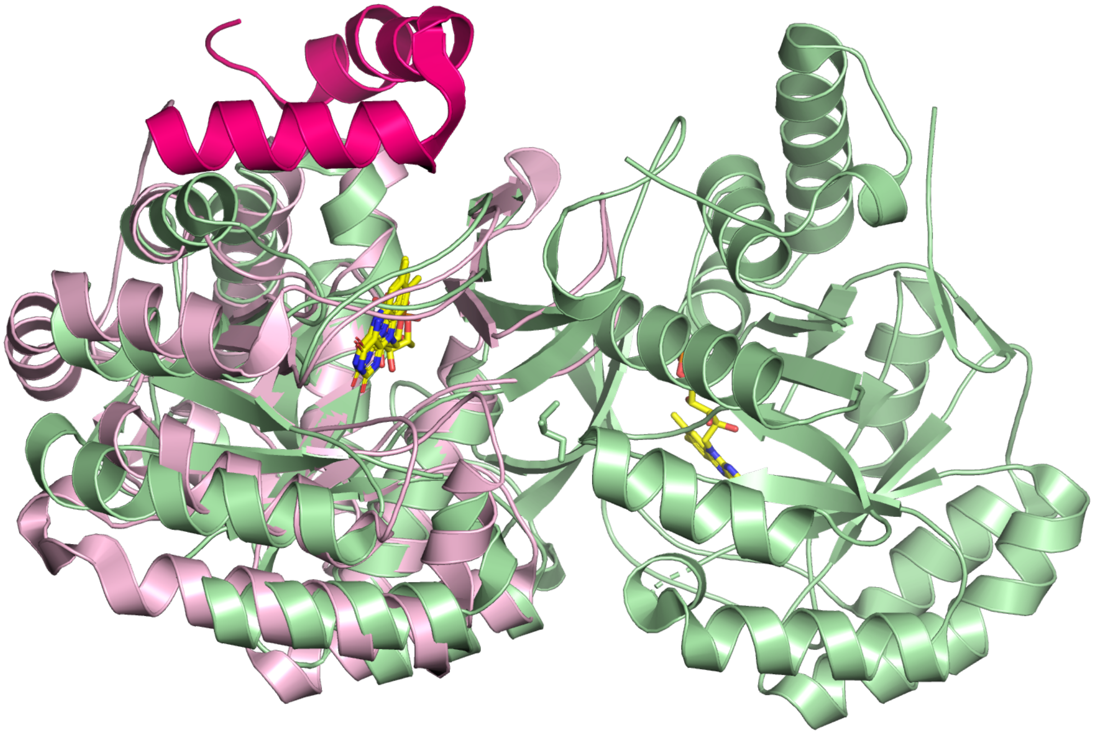

Table 1S: Data collection and processing

**Data collection**					
	**Orotate**	**2a**	**2c**	**2g**	**3c**
PDB ID	9N67	9CB8	9N6O	9N68	9N6Q
Resolution range (Å)	47.72 - 1.8 (1.9 - 1.8)	39.89 - 1.75 (1.77 - 1.75)	47.74 - 1.90 (1.92- 1.9)	47.57 - 1.448 (1.46 - 1.45)	47.6 - 1.85 (1.87 - 1.85)
Space group	P 21 21 21	P 21 21 21	P 21 21 21	P 21 21 21	P 21 21 21
**Unit cell**					
a, b, c (Å)	61.98 105.456 106.095	61.54 104.78 106.84	62.04 104.29 107.21	61.57 104.89 106.74	61.72 105.47 106.12
α, β, γ (º)	90 90 90	90 90 90	90 90 90	90 90 90	90 90 90
Total reflections	759133 (102992)	953797 (30095)	745081 (24362)	1619358 (253102)	805598 (25858)
Unique reflections	65215 (9371)	133352 (4337)	105966 (3555)	235369 (19235)	114236 (3836)
Completeness (%)	99.98 (100.00)	99.04 (97.19)	99.95 (99.16)	98.88 (97.5)	99.98 (99.92)
Multiplicity	11.6 (11.0)	7.2 (6.9)	7.0 (6.9)	6.9 (6.9)	7.1 (6.7)
Mean I/sigma(I)	14.9 (2.1)	10.67 (1.0)	7.7 (1.1)	12.59 (1.1)	12.0 (1.0)
R-merge	0.1973 (1.607)	0.08265 (3.541)	0.2163 (4.869)	0.05982 (3.067)	0.09794 (4.074)
$CC_{1/2}$	0.998 (0.696)	0.999 (0.482)	0.997 (0.436)	0.999 (0.521)	0.999 (0.461)

CC*	0.999 (0.854)	1 (0.58)	0.999 (0.47)	1 (0.596)	1 (0.475)
**Refinement**					
Solvent molecules	564	263	130	424	266
R-work	0.1648 (0.2258)	0.1689 (0.4106)	0.1820 (0.3949)	0.1457 (0.4141)	0.1738 (0.4080)
R-free	0.2025 (0.2656)	0.1955 (0.4101)	0.2165 (0.3745)	0.1722 (0.4643)	0.2099 (0.4625)
**Number of non-hydrogen atoms**	5356	5029	4835	5206	4936
Macromolecules	4670	4636	4583	4650	4555
Ligands	122	130	122	132	115
Solvent	564	263	130	424	266
**B-factors**					
Macromolecules	27.98	43.92	41.92	33.03	50.58
Ligands	30.58	48.25	48.47	38.56	59.74
Solvent	38.18	48.32	47.71	40.20	51.30
Average	29.11	44.26	42.08	33.76	50.83
**r.m.s. deviations**					
Bond length	0.014	0.008	0.008	0.009	0.007
Bond angles	1.26	0.98	0.99	1.17	0.85
**Ramachandran plot**					
Favored (%)	96.89	96.68	97.04	97.50	97.00

Allowed (%)	3.11	3.32	2.96	2.50	3.00
Outliers (%)	0.00	0.00	0	0.00	0.00
Rotamer outliers (%)	0.00	0.00	0	0.00	0.00

Table 2S. Results of the Single Dose Assays of **2a-i** and **3a-3ii** against *Hs*DHODH

**ID**	**Single dose (0h) % activity**	**Single dose (4h) % activity**	**ID**	**Single dose (0h) % activity**	**Single dose (4h) % activity**
**2a**	87±10	79±8	**3b**	92 ±10	83±8
**2b**	105 ±12	99±10	**3c**	87±10	79±8
**2c**	80±9	83±8	**3e**	105 ±12	89±9
**2d**	90±10	92±9	**3f**	93±11	102±10
**2e**	80±9	86±8	**3g**	87±10	86±10
**2f**	103±12	78±8	**3h**	89 ± 10	91±9
**2g**	101± 11	96±9	**3i**	102±12	98±10
**2h**	100±11	71± 7	**3ii**	99±11	96±9
**2i**	93 ± 11	91±9	**Teriflunomide**	0.6±0.1	0.39±0.04
**3a**	109±12	97±9			

Compounds were tested at [100 μM]. Teriflunomide was used as experimental positive control

Figure 3S. IC50 determination without (0h) and with (4h) incubation for barbituric acid derivatives.

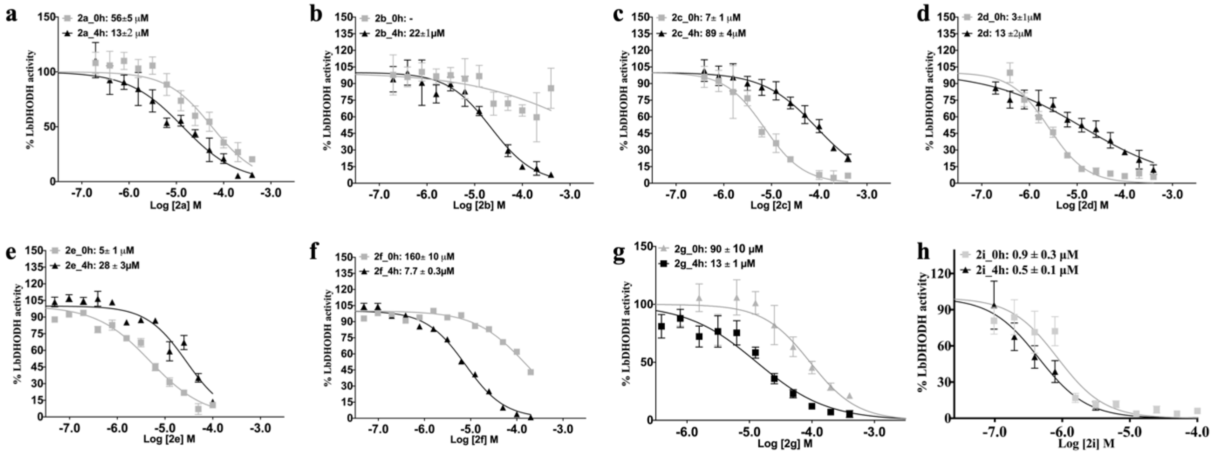

Figure 4S. K*inact*/K*I* determination for **2a**, **2d**, **2g** and **2i**.

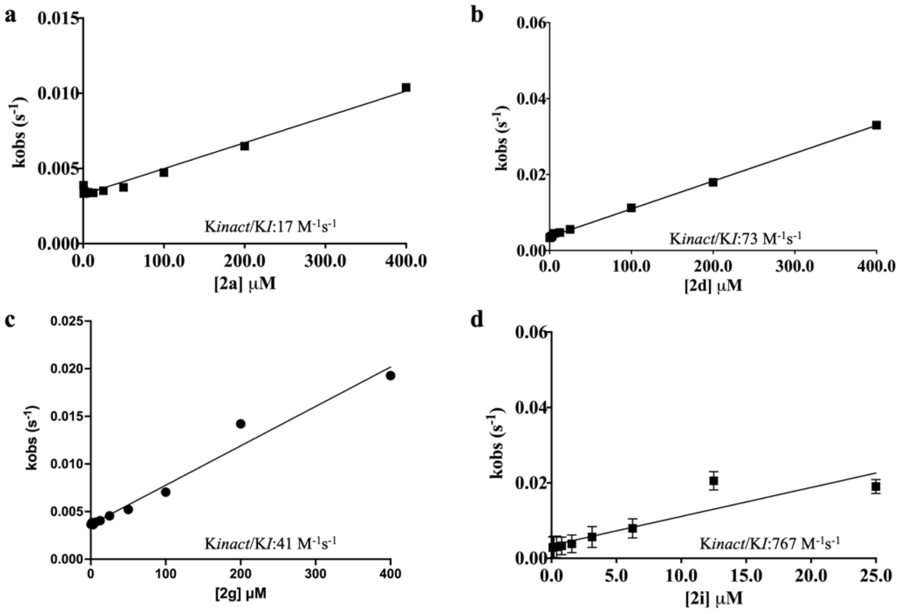

1H NMR (400MHz, DMSO-d6) of pyrimidine-2,4,6(1H,3H,5H)-trione (1)

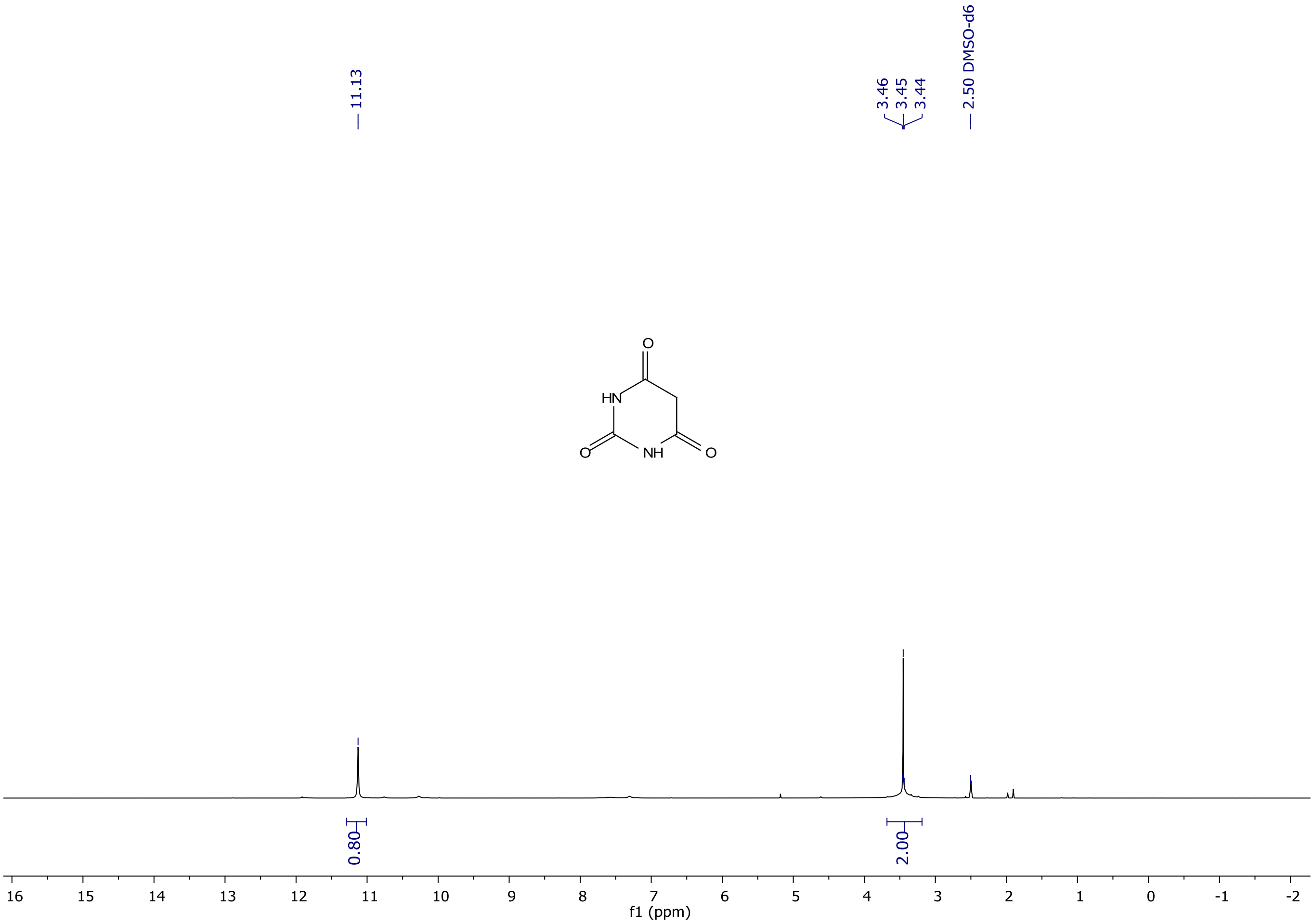

13C NMR (75MHz, DMSO-d6) of pyrimidine-2,4,6(1H,3H,5H)-trione (1)

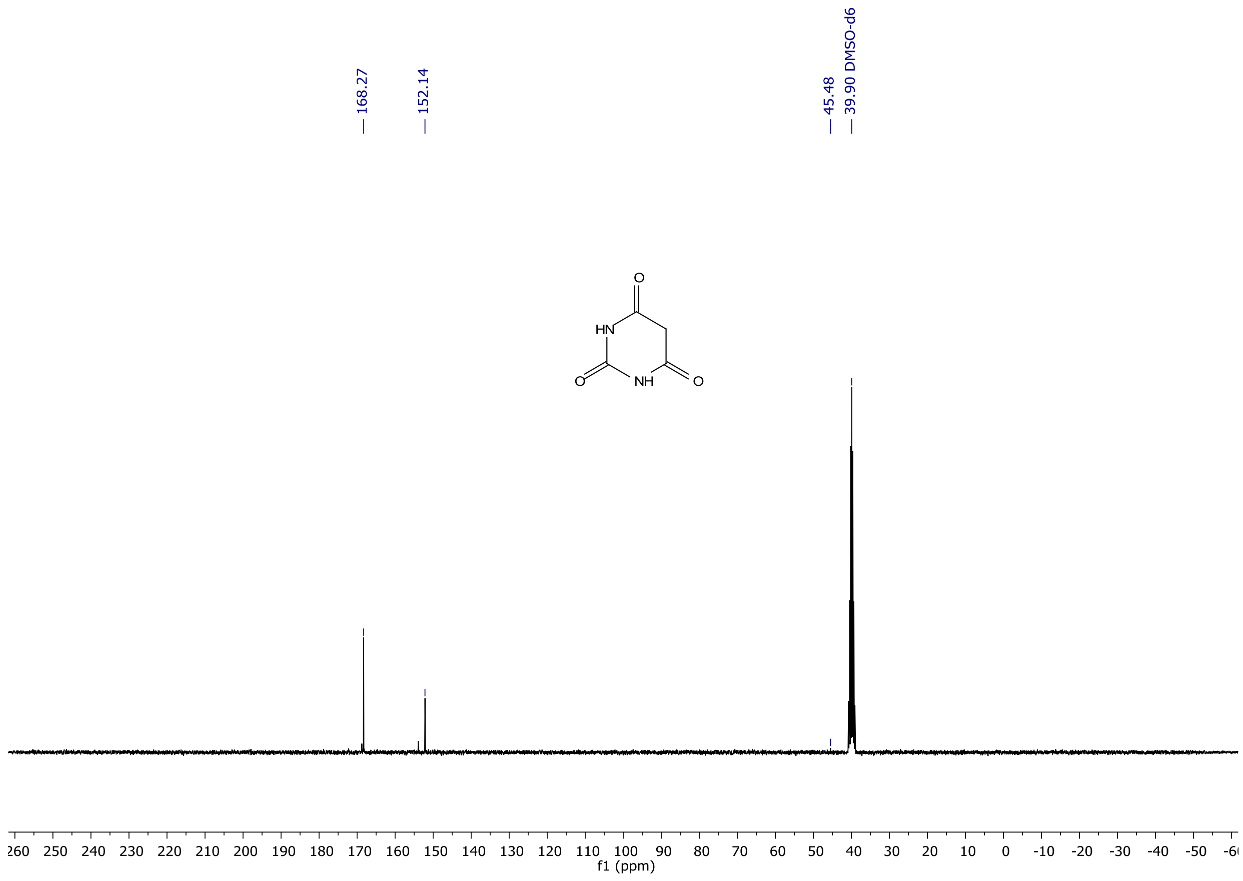

1H NMR (400MHz, DMSO-d6) of 5-benzylidenepyrimidine-2,4,6(1H,3H,5H)-trione (2a)

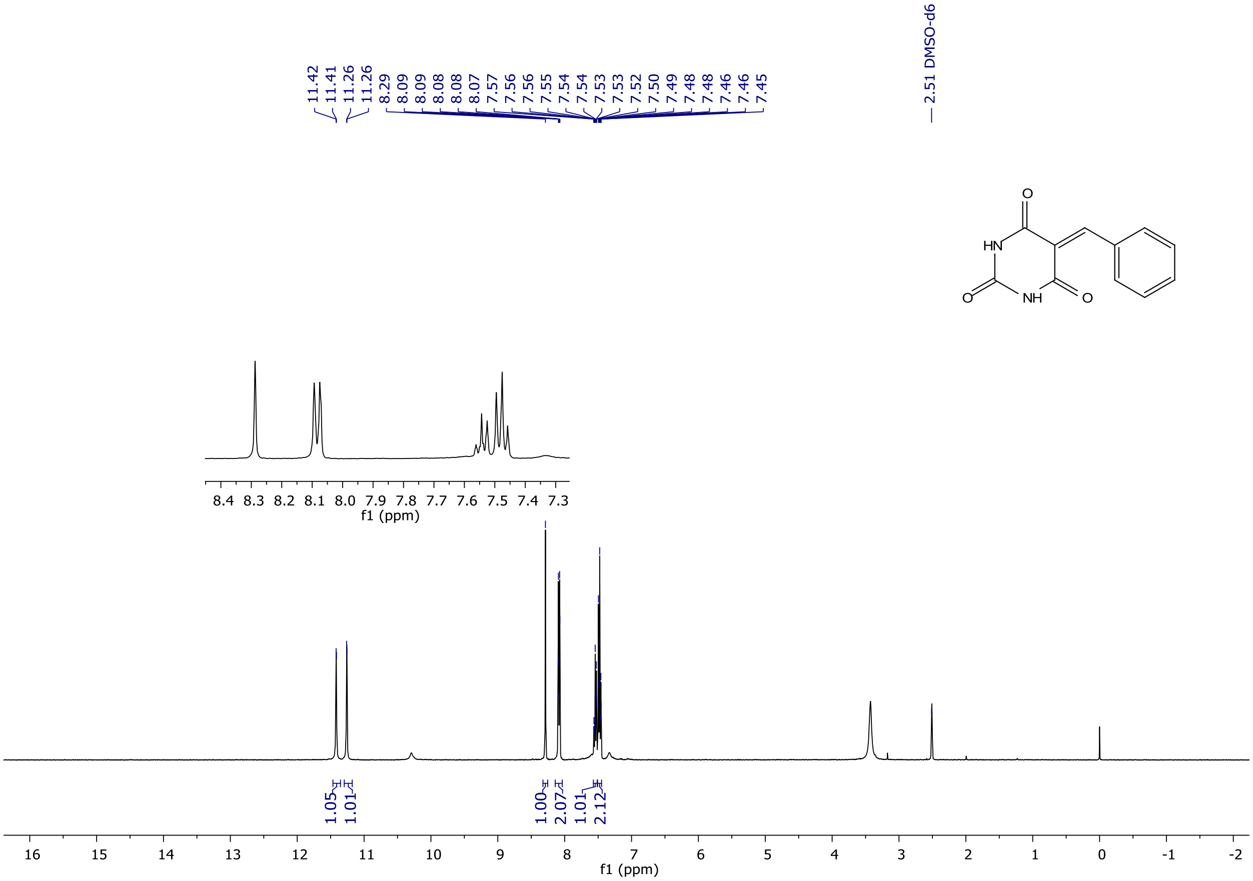

^{1}H NMR (400MHz, DMSO-d$_6$) of 4-((2,4,6-trioxotetrahydropyrimidin-5(2H)-ylidene)methyl)benzoic acid (**2b**)

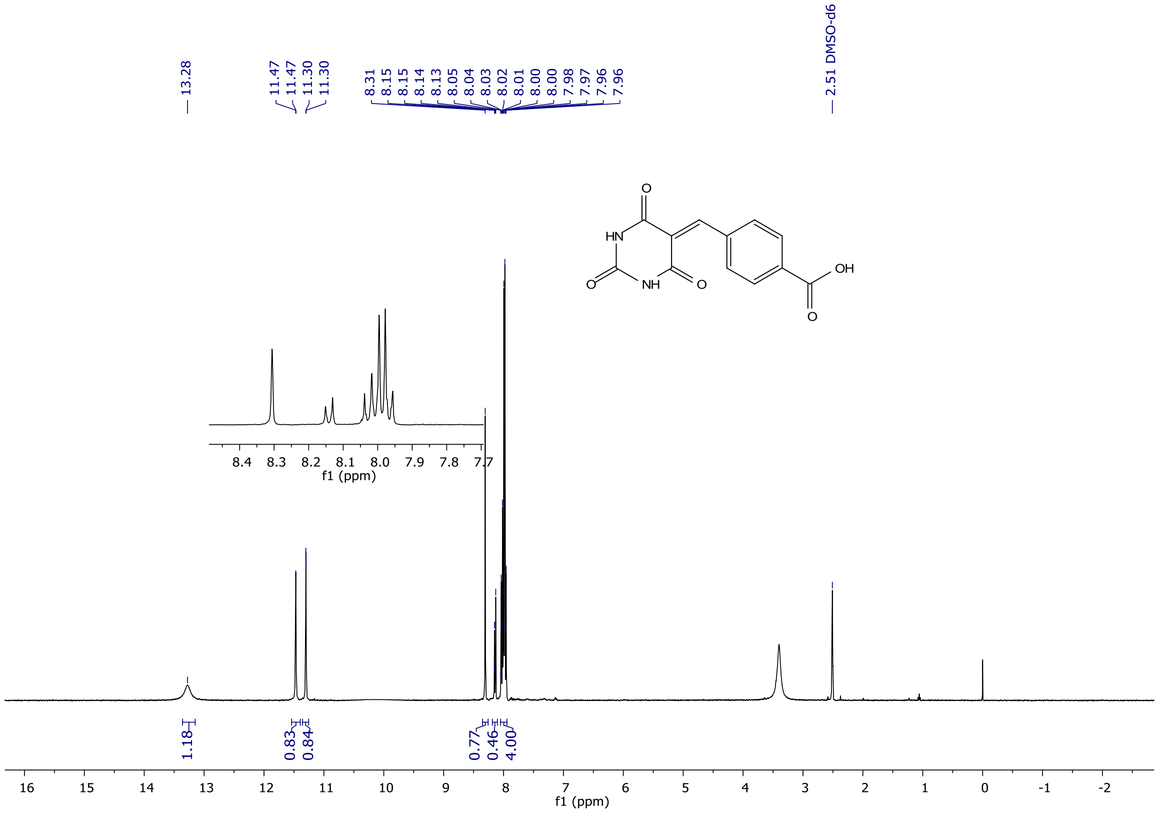

^{1}H NMR (400MHz, DMSO-d6) of 5-(4-hydroxy-3-methoxybenzylidene)pyrimidine-2,4,6(1H,3H,5H)-trione (**2c**)

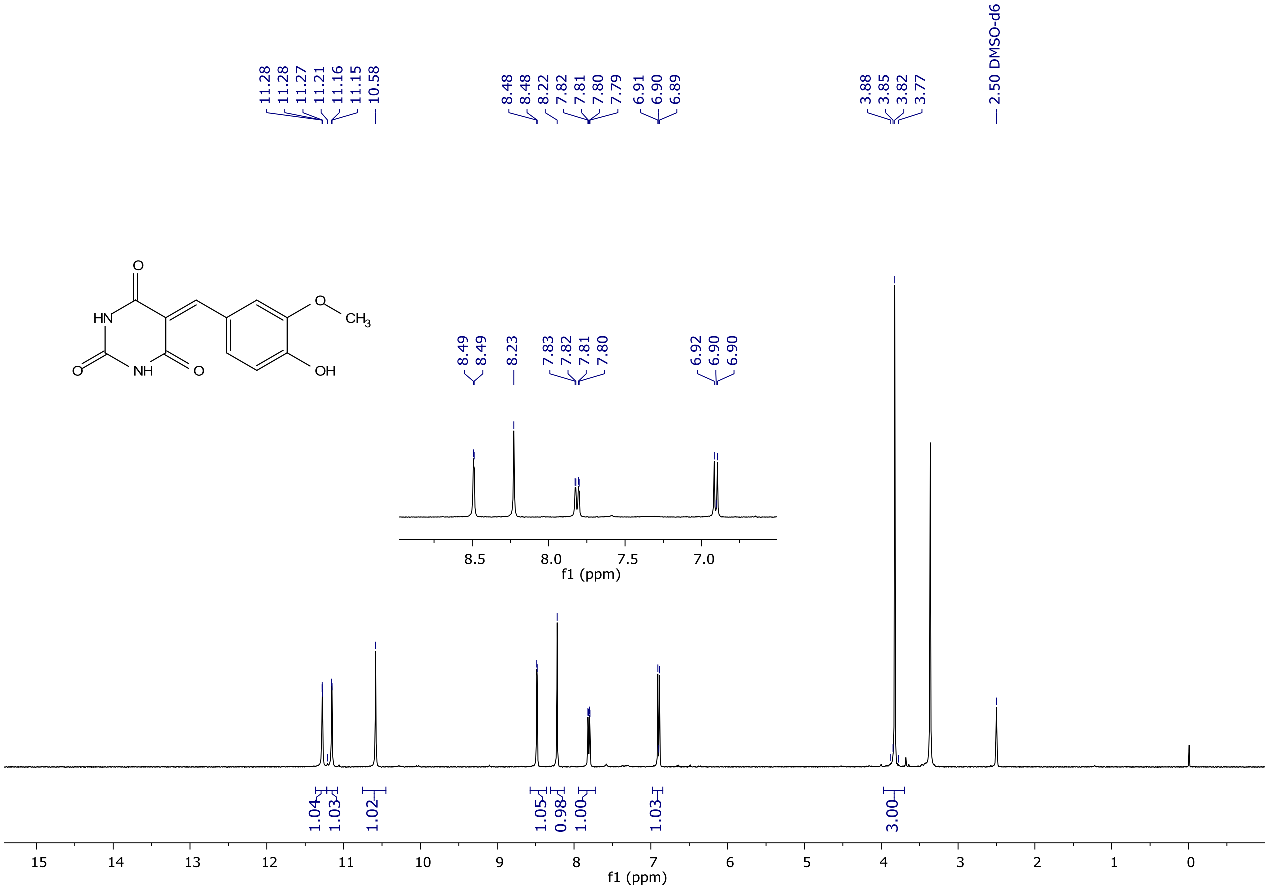

^{1}H NMR (400MHz, DMSO-d_6) of 5-(4-hydroxy-3-nitrobenzylidene)pyrimidine-2,4,6(1H,3H,5H)-trione (**2d**)

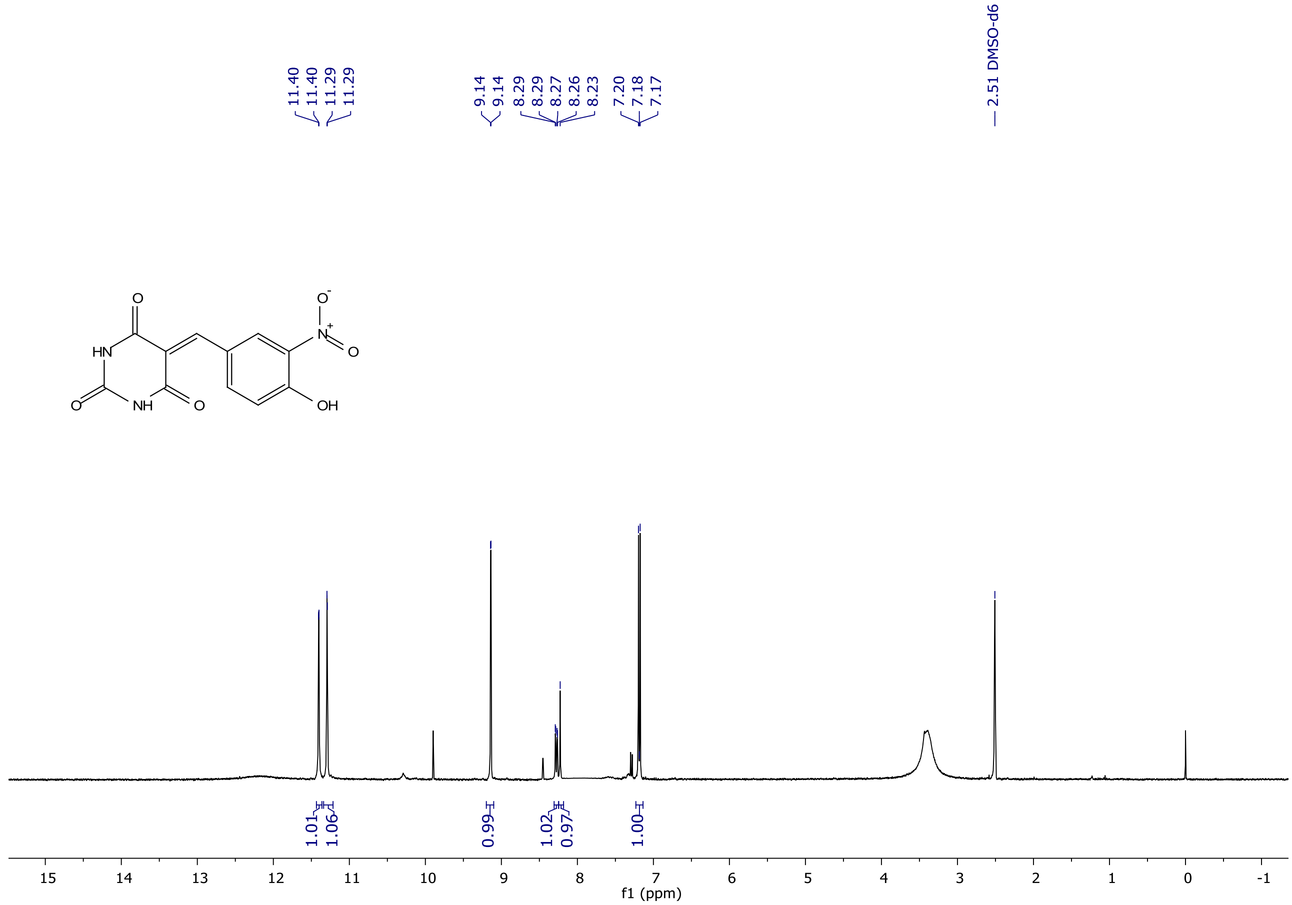

1H NMR (400MHz, DMSO-d6) of 5-(4-morpholino-2-nitrobenzylidene)pyrimidine-2,4,6(1H,3H,5H)-trione (2e)

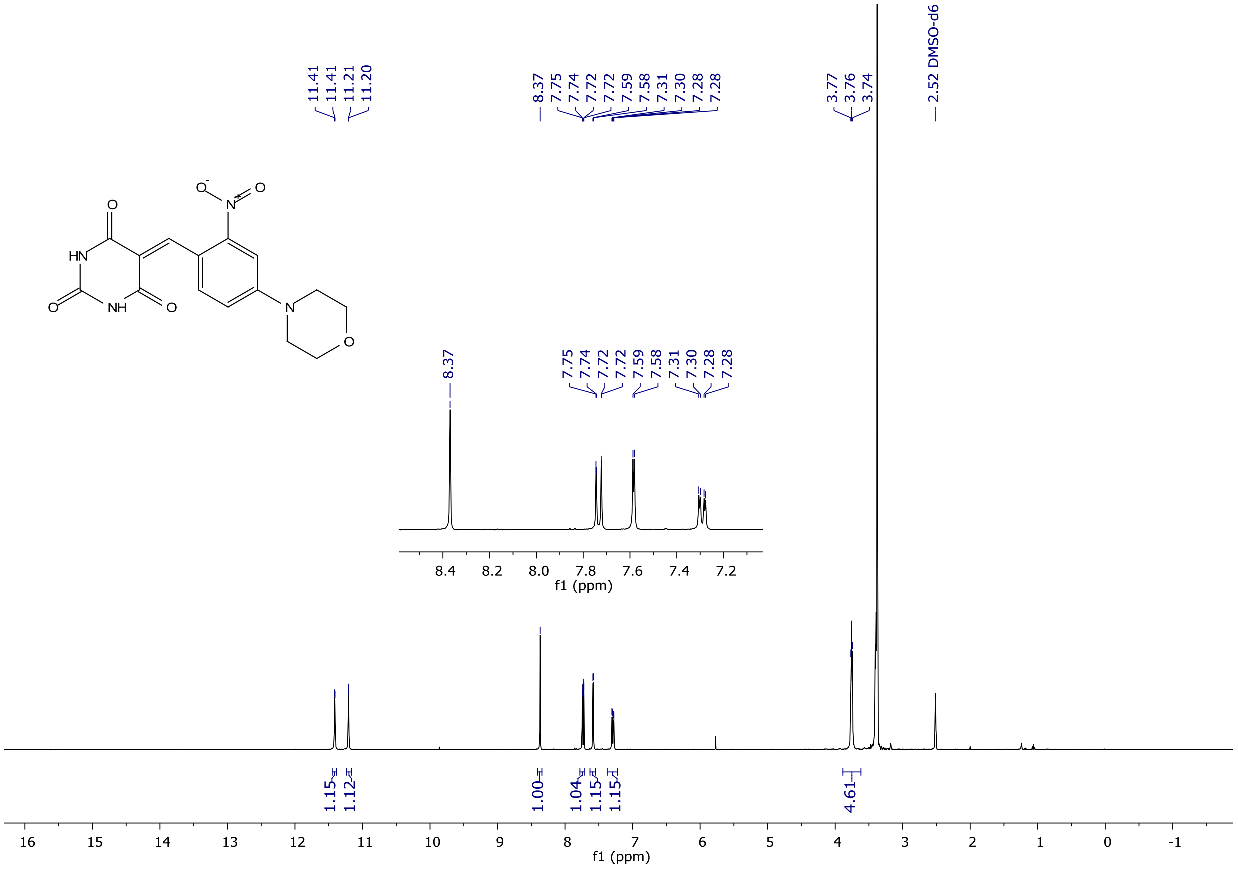

^1H NMR (400MHz, DMSO-d_6) of 5-([1,1'-biphenyl]-4-ylmethylene)pyrimidine-2,4,6(1H,3H,5H)-trione (**2f**)

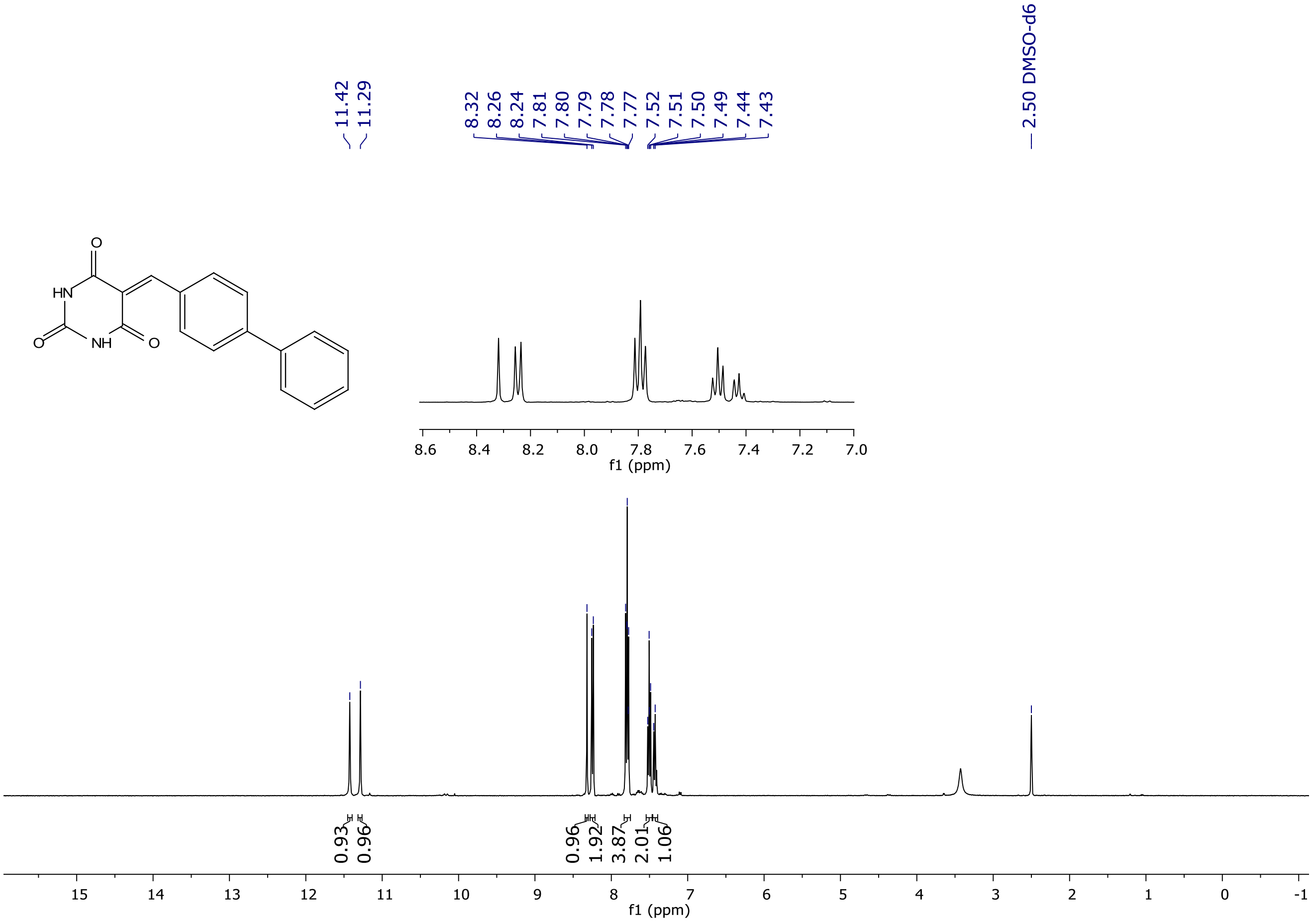

^{1}H NMR (400MHz, DMSO-d_6) of 5-(3-hydroxybenzylidene)pyrimidine-2,4,6(1H,3H,5H)-trione (**2g**)

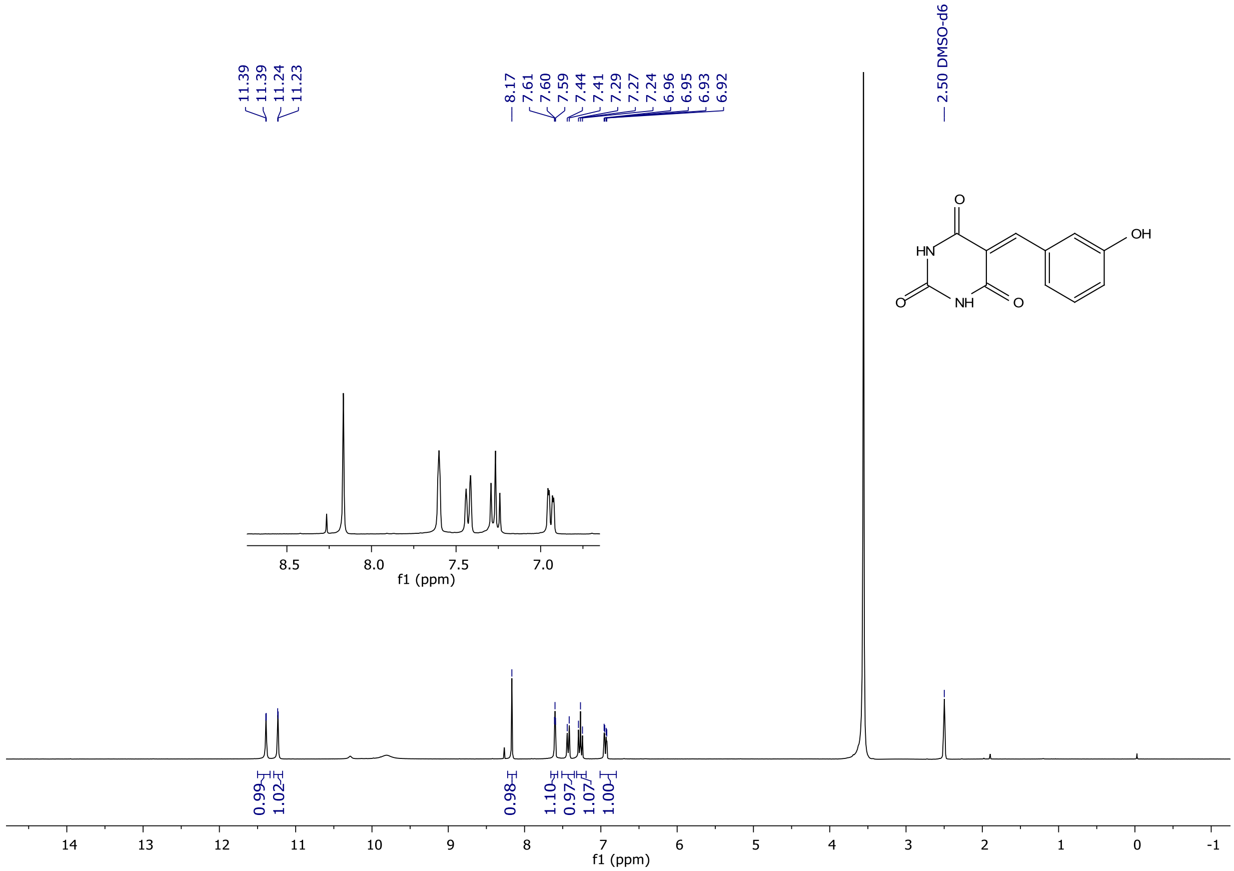

^{1}H NMR (400MHz, DMSO-d_6) of 5-((5-(2-fluorophenyl)-1H-pyrrol-2-yl)methylene)pyrimidine-2,4,6(1H,3H,5H)-trione (**2h**)

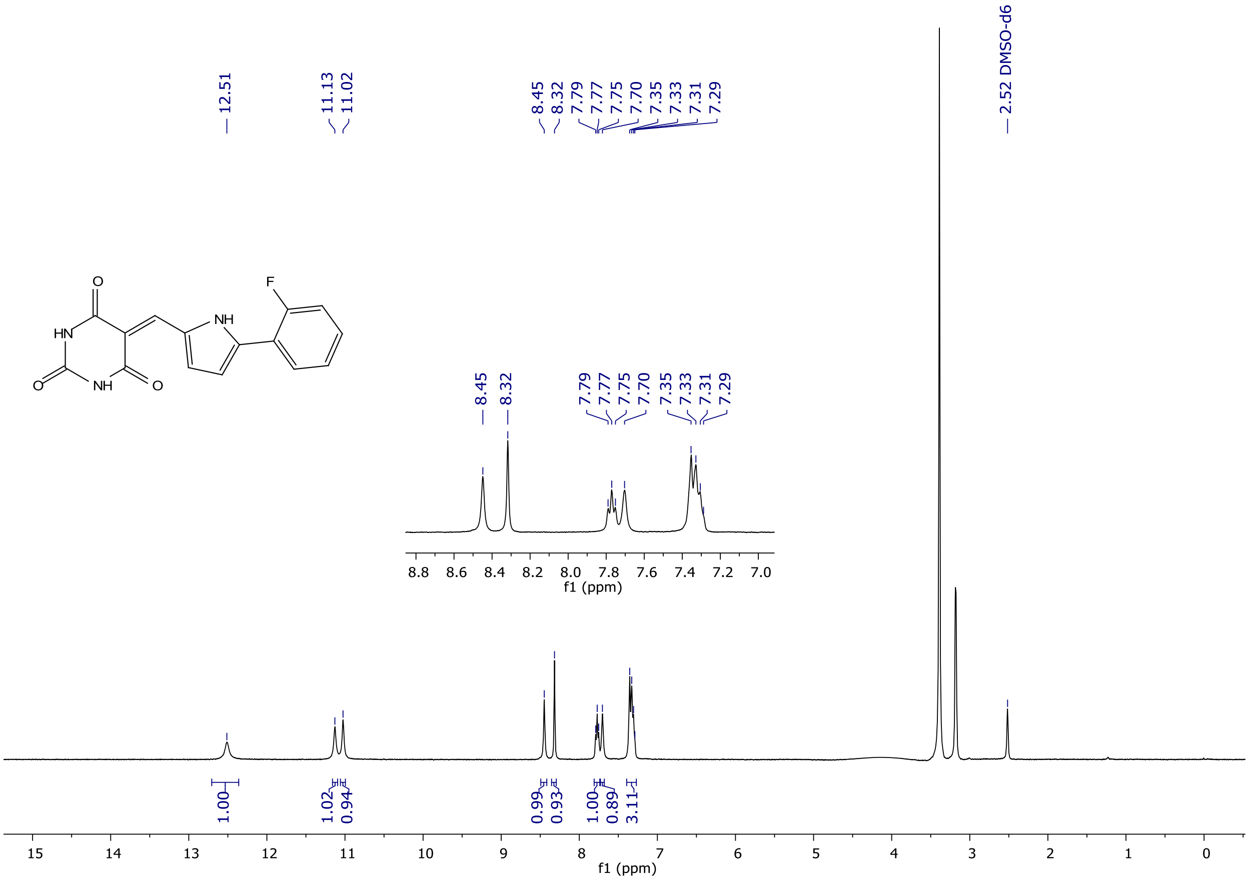

^{1}H NMR (400MHz, DMSO-d$_6$) of (E)-5-(3-phenylallylidene)pyrimidine-2,4,6(1H,3H,5H)-trione (**2i**)

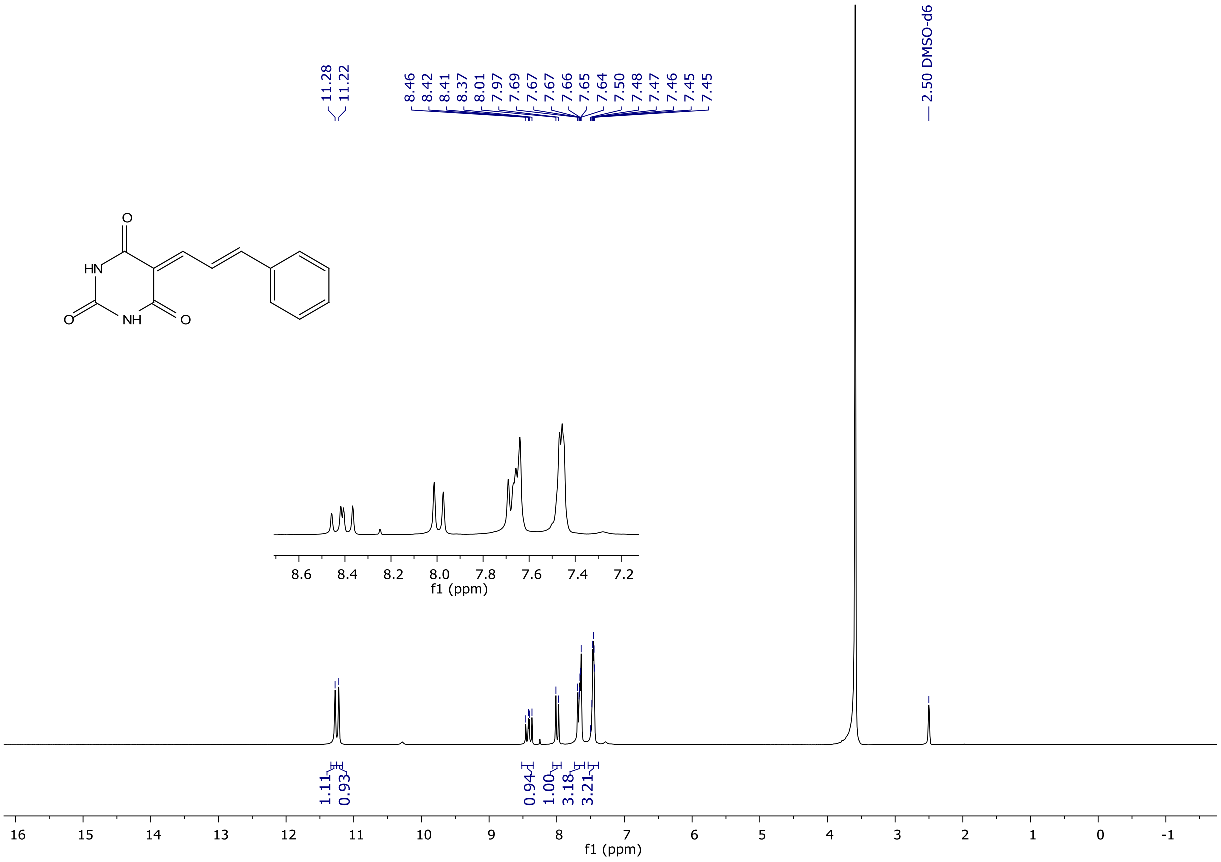

^{13}C NMR (75 MHz, DMSO-d_6) of (E)-5-(3-phenylallylidene)pyrimidine-2,4,6(1H,3H,5H)-trione (**2i**)

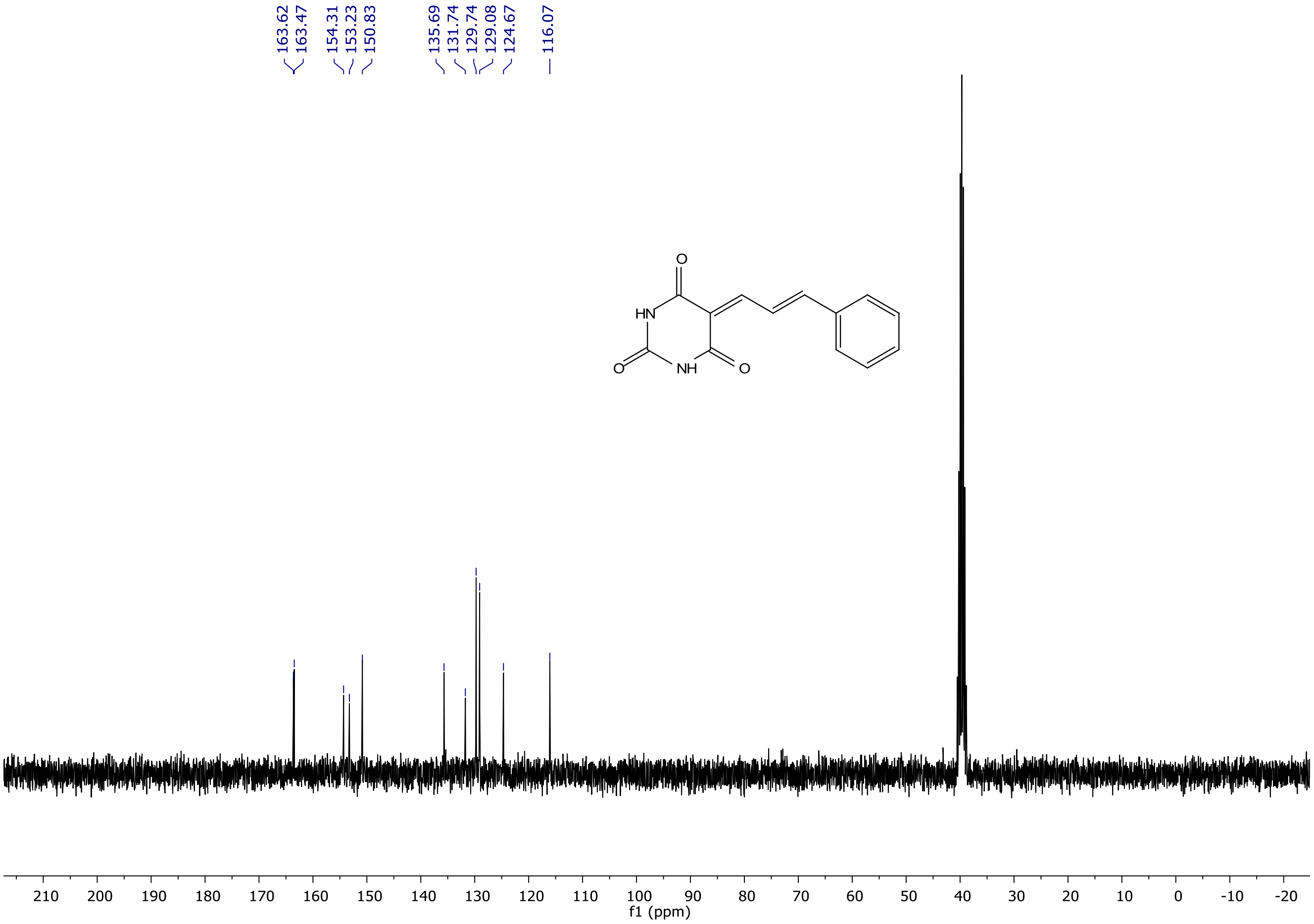

^{1}H NMR (400MHz, DMSO-d$_6$) of 5-benzylpyrimidine-2,4,6(1*H*,3*H*,5*H*)-trione (**3a**):

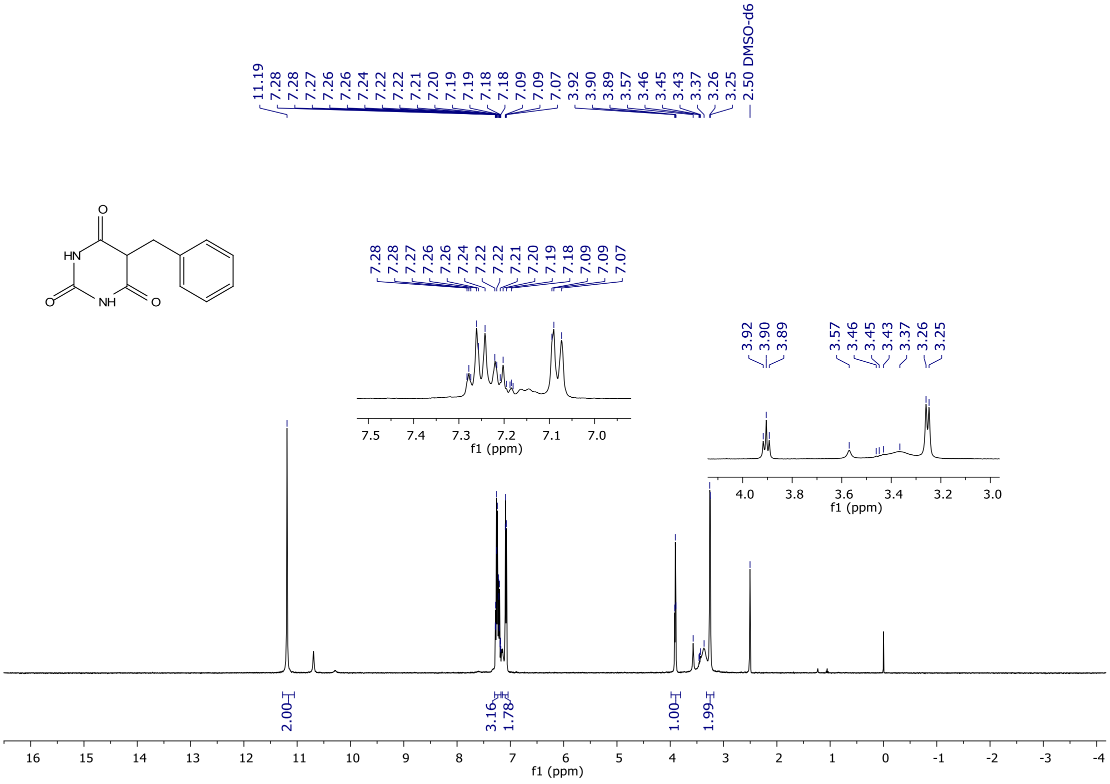

^{1}H NMR (400MHz, DMSO-d$_6$) of 4-((2,4,6-trioxohexahydropyrimidin-5-yl)methyl)benzoic acid (**3b**)

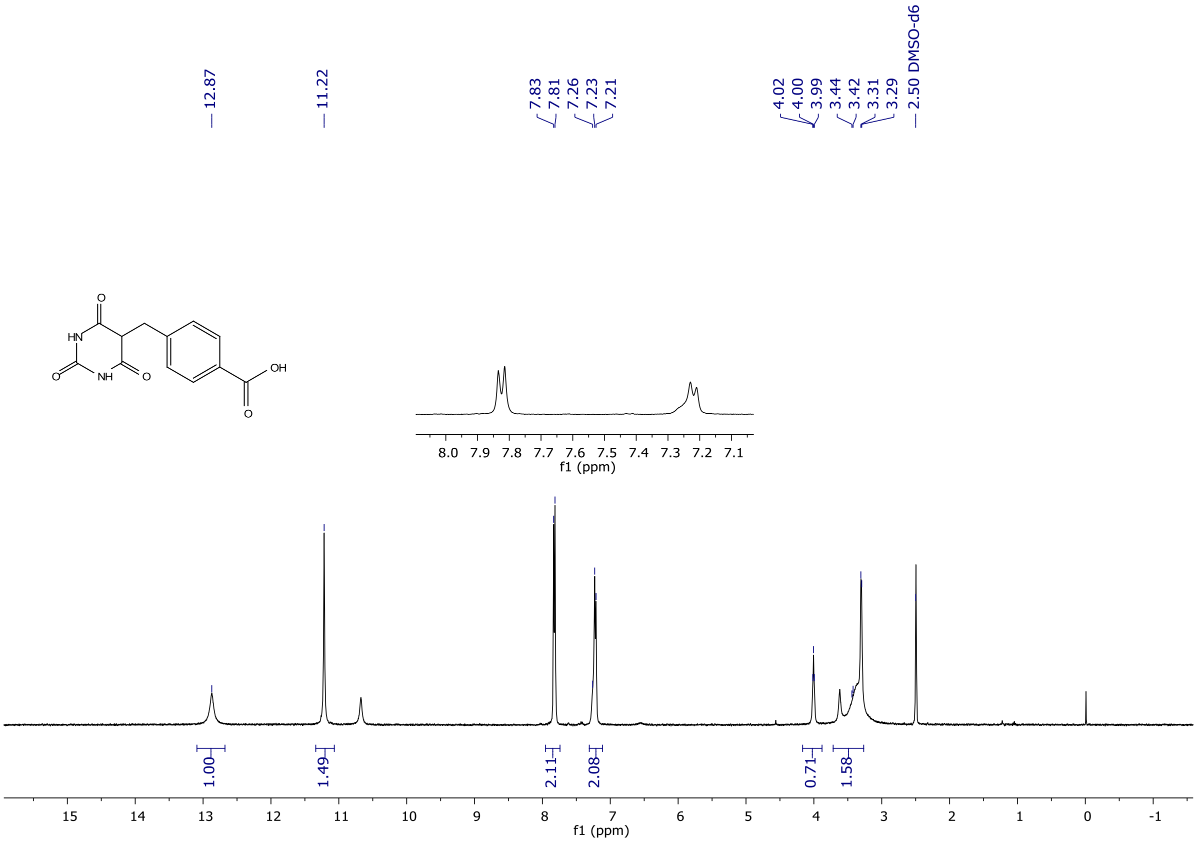

FTIR spectra of 4-((2,4,6-trioxohexahydropyrimidin-5-yl)methyl)benzoic acid (**3b**)

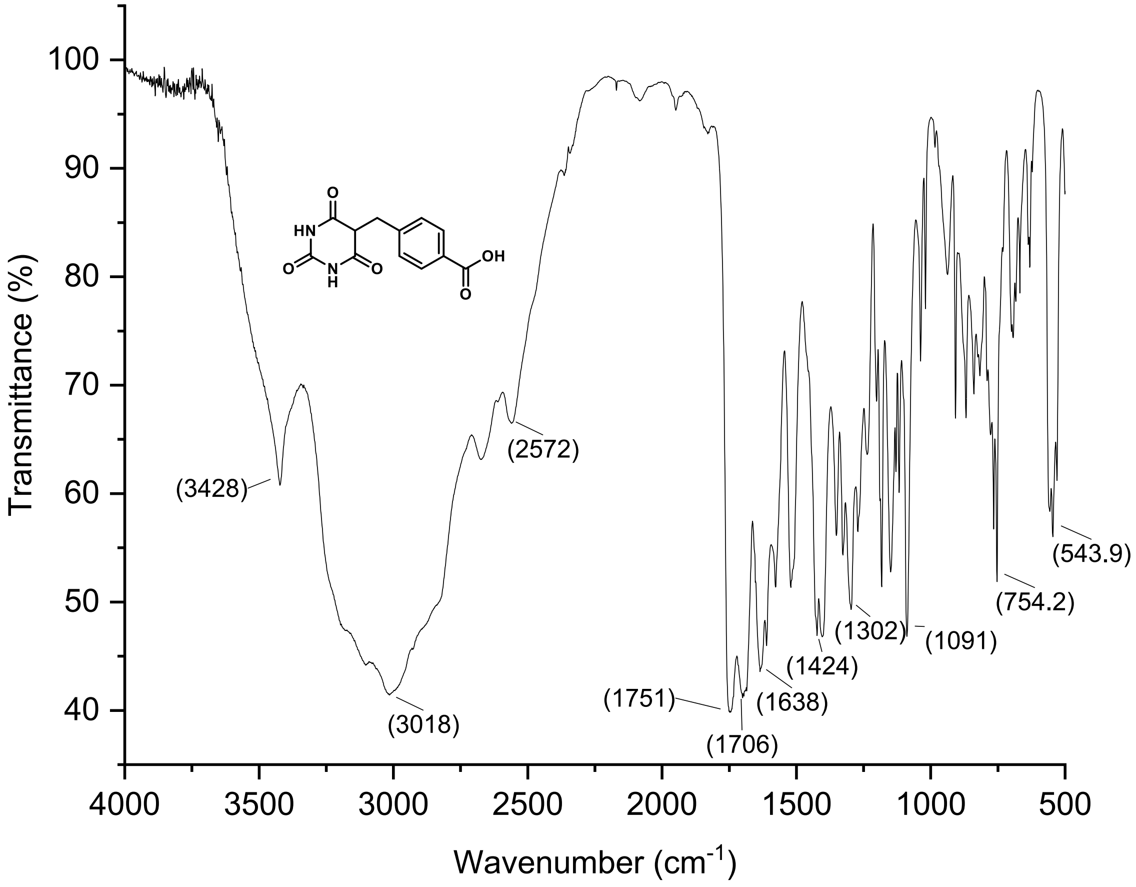

^{1}H NMR (400MHz, DMSO-d$_6$) of 5-(4-hydroxy-3-methoxybenzyl)pyrimidine-2,4,6(1H,3H,5H)-trione (**3c**)

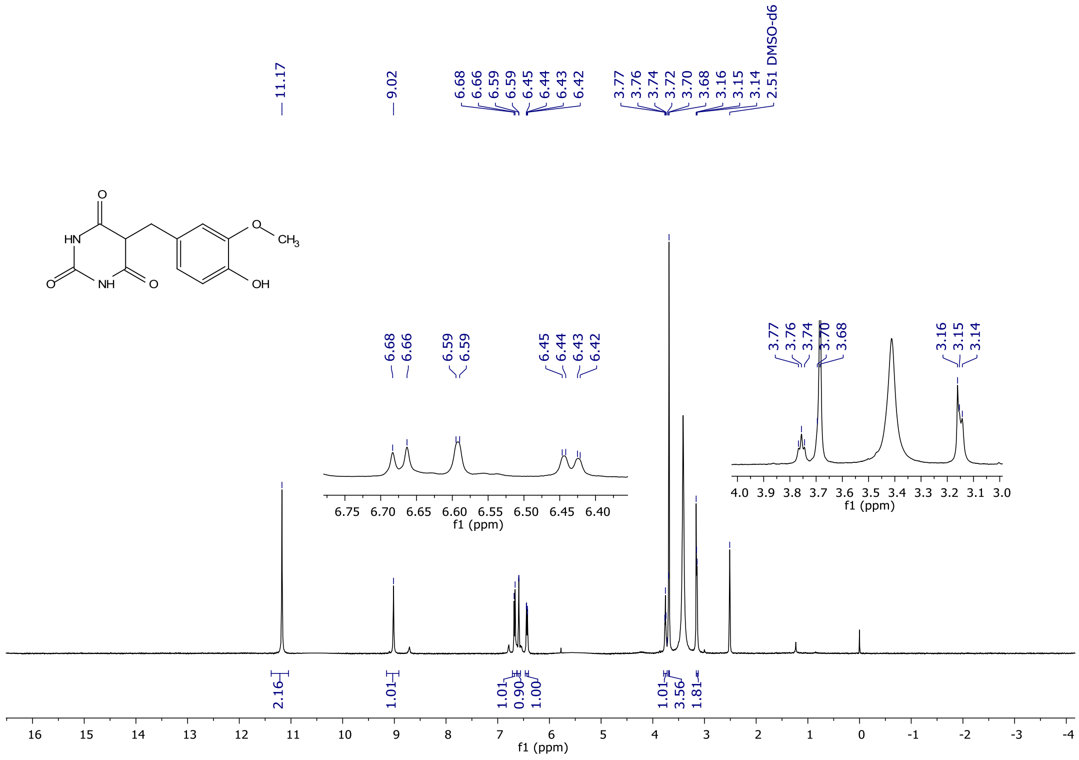

^{1}H NMR (400MHz, DMSO-d_6) of 5-(4-morpholino-2-nitrobenzyl)pyrimidine-2,4,6(1H,3H,5H)-trione (**3e)**

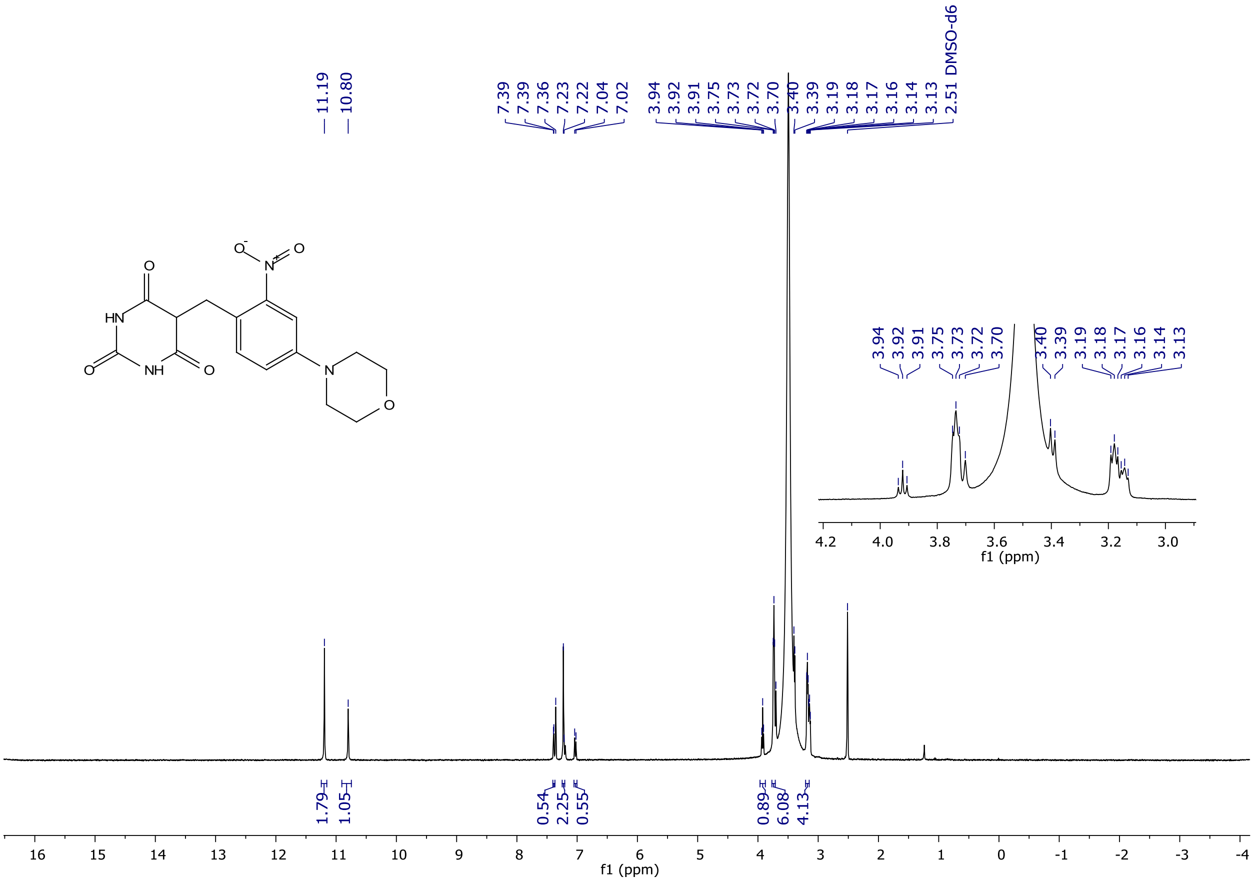

FTIR of 5-(4-morpholino-2-nitrobenzyl)pyrimidine-2,4,6(1H,3H,5H)-trione (**3e**)

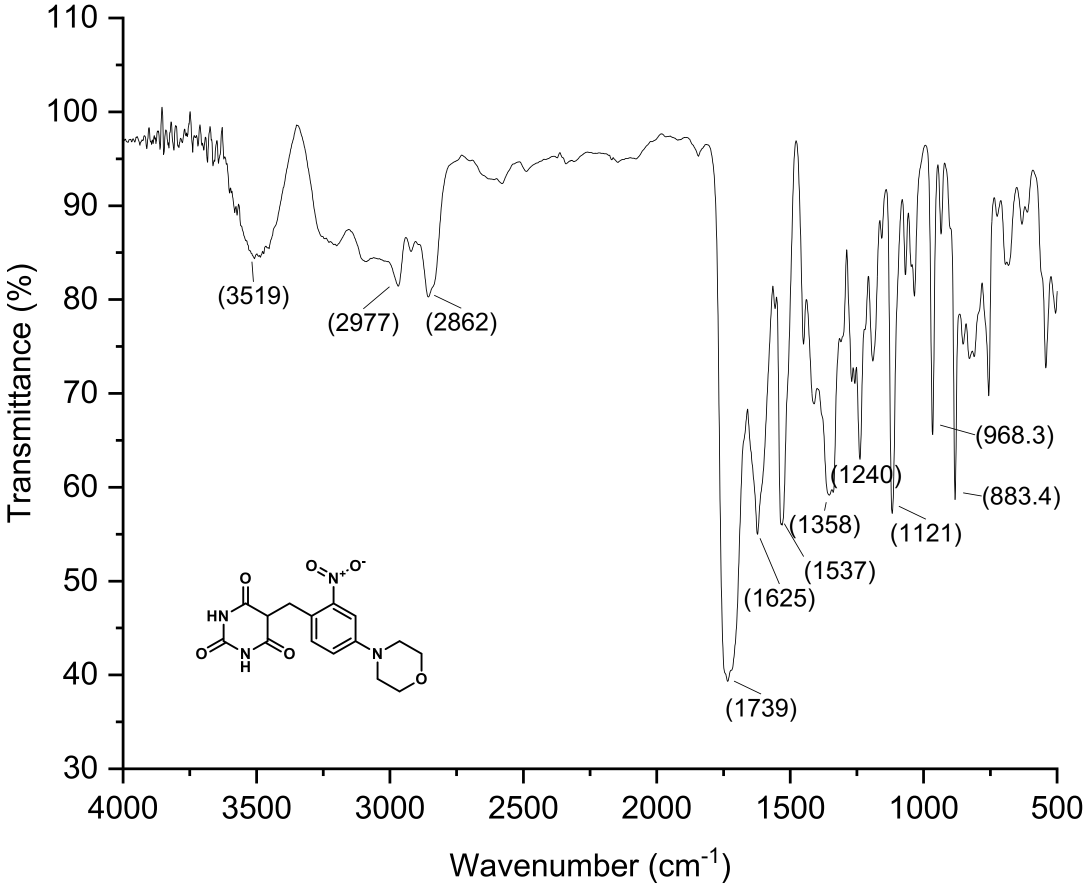

^{1}H NMR (400MHz, DMSO-d_6) of 5-([1,1'-biphenyl]-4-ylmethyl)pyrimidine-2,4,6(1H,3H,5H)-trione (**3f**)

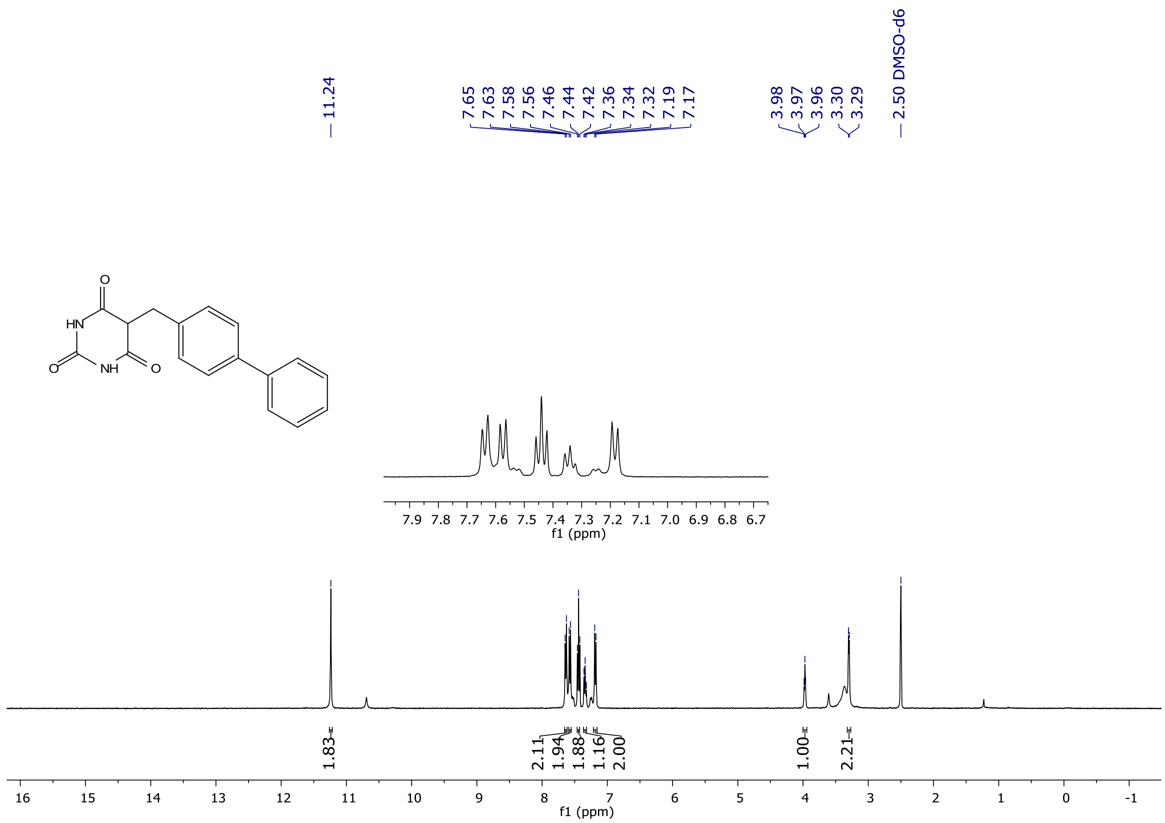

^{1}H NMR (400MHz, DMSO-d$_6$) of 5-(3-hydroxybenzyl)pyrimidine-2,4,6(1H,3H,5H)-trione (**3g**)

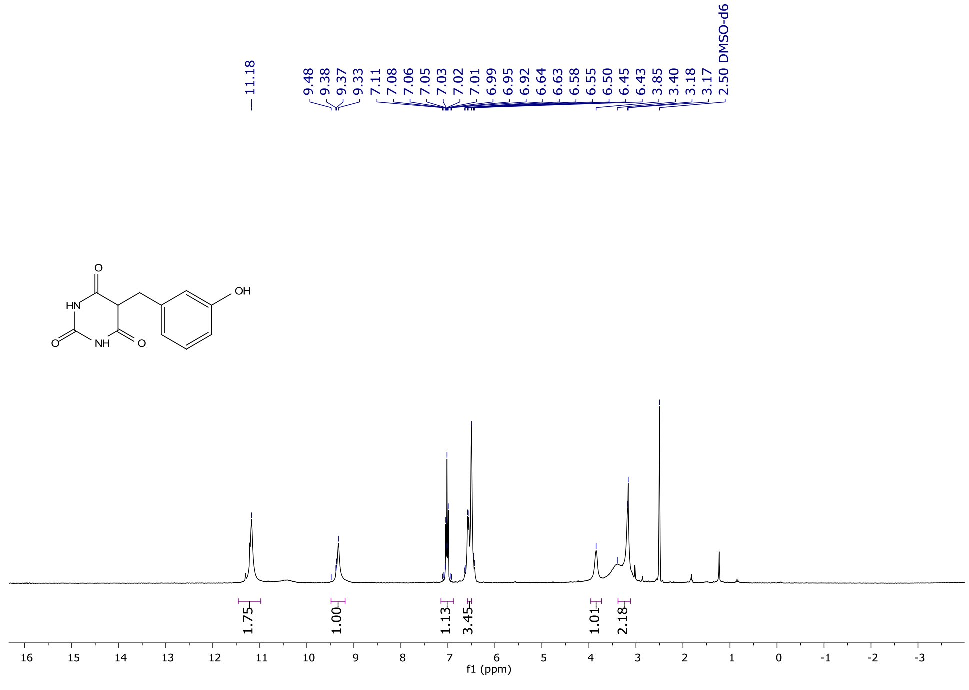

^{1}H NMR (400MHz, DMSO-d$_6$) of 5-((5-(2-fluorophenyl)-1H-pyrrol-2-yl)methyl)pyrimidine-2,4,6(1H,3H,5H)-trione (**3h)**

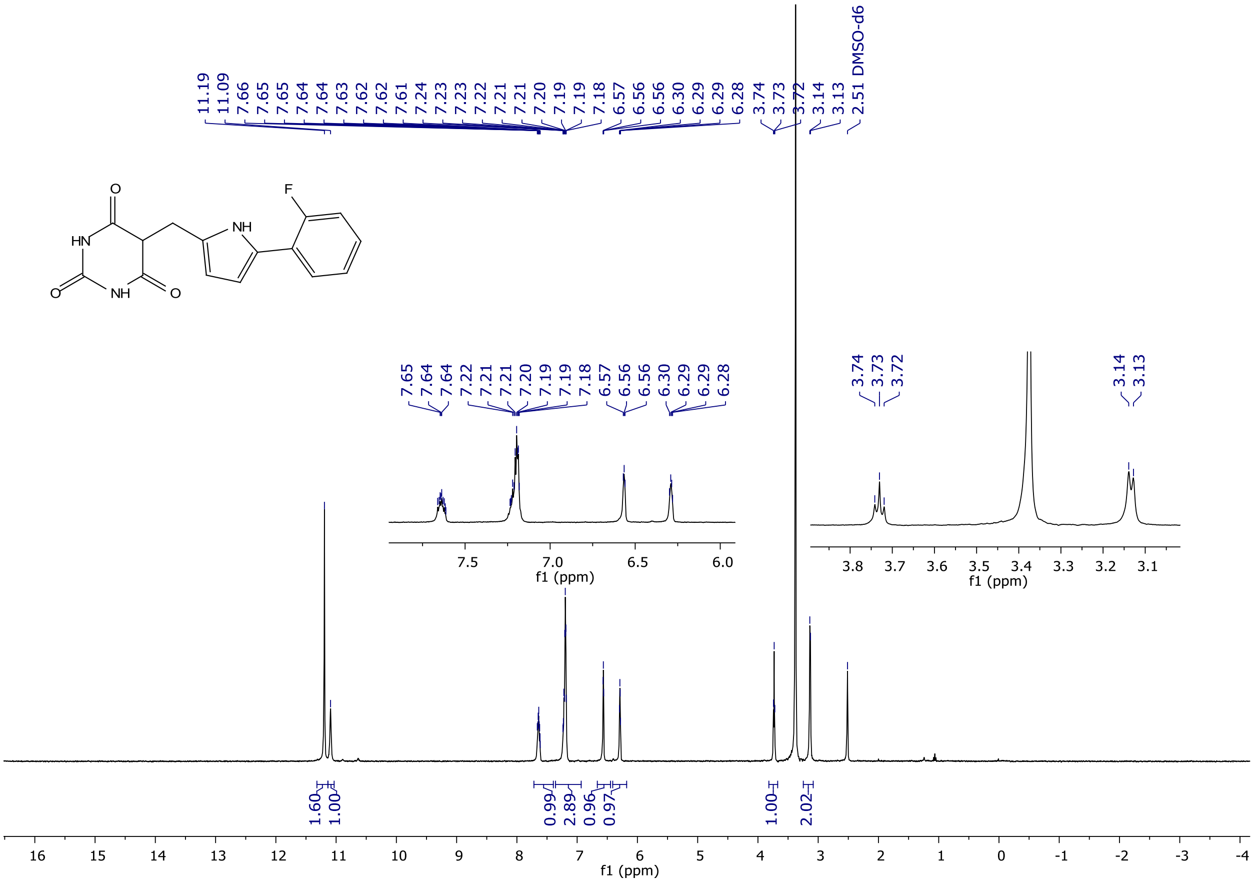

FTIR of 5-((5-(2-fluorophenyl)-1H-pyrrol-2-yl)methyl)pyrimidine-2,4,6(1H,3H,5H)-trione (**3h)**

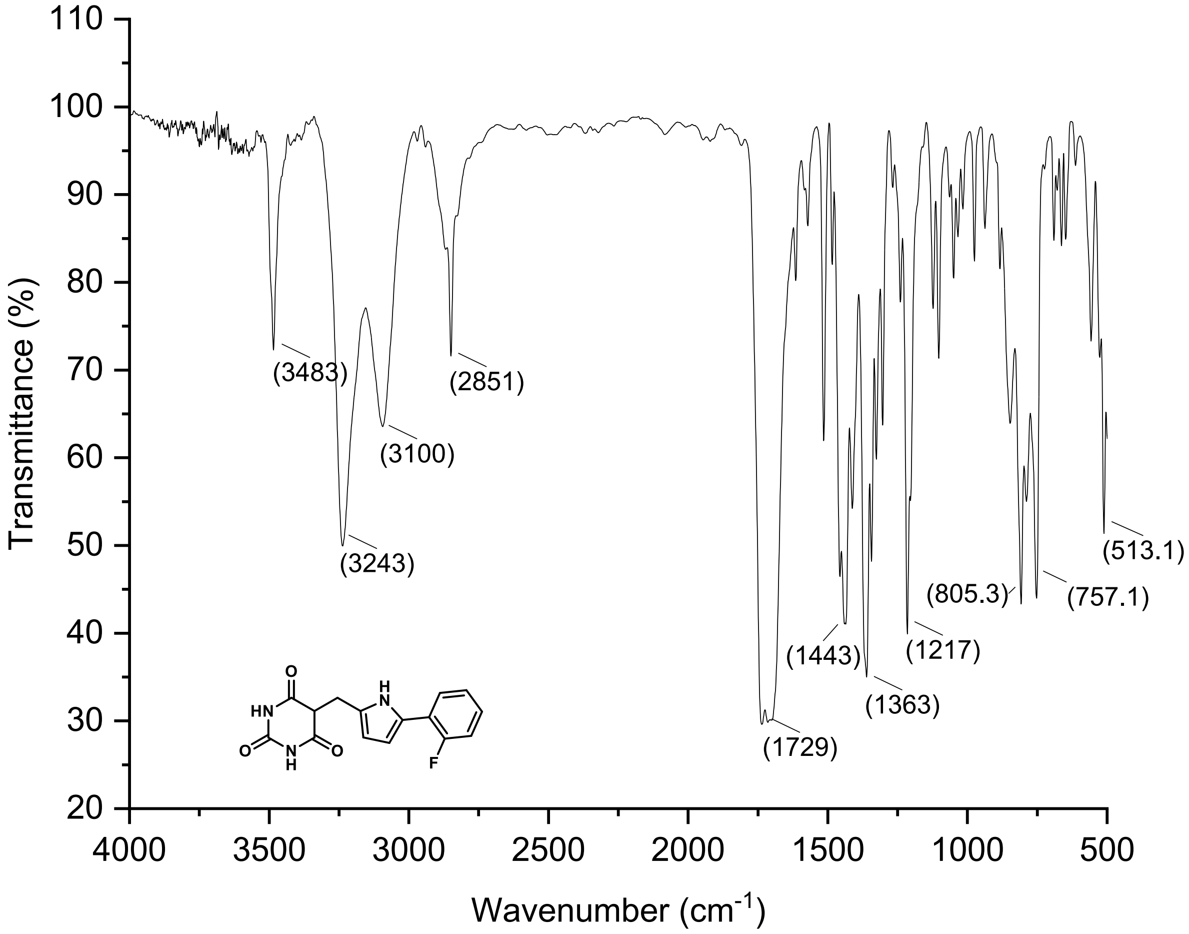

^{1}H NMR (400MHz, DMSO-d$_6$) of 5-(3-phenylpropyl)pyrimidine-2,4,6(1*H*,3*H*,5*H*)-trione **(3i)**

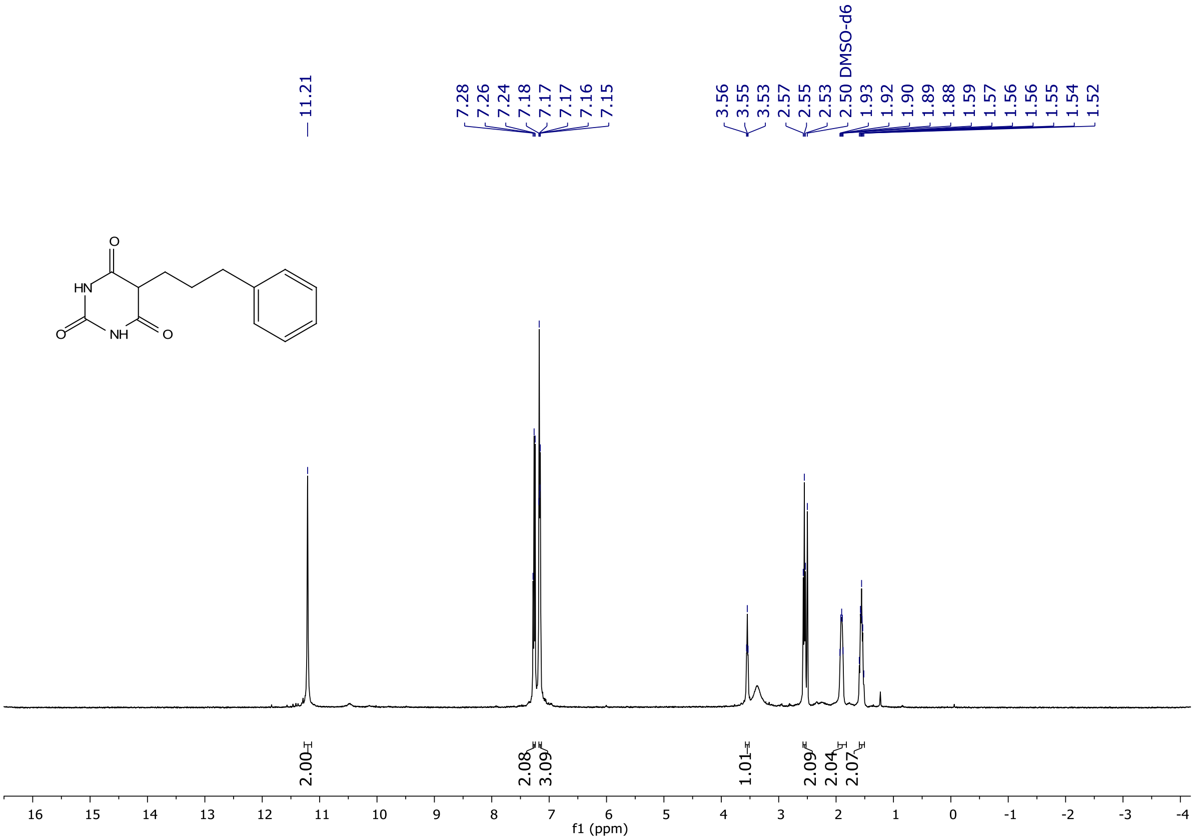

^{1}H NMR (400MHz, DMSO-d_6) of 5-cinnamylpyrimidine-2,4,6(1H,3H,5H)-trione (**3ii**)

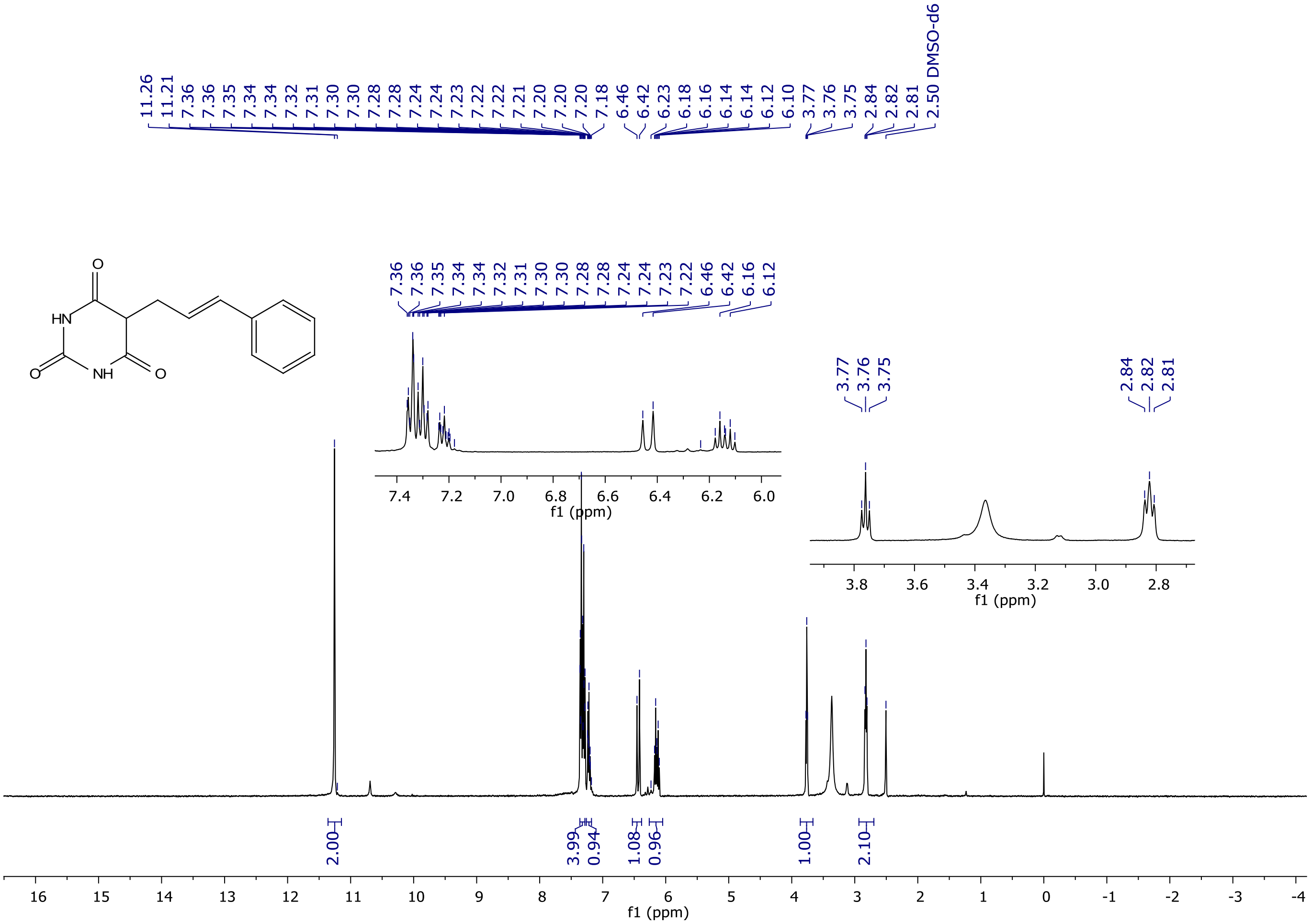

Purity of compounds qNMR

Software-controlled and hardware-dependent Parameters:

NMR Instrument: Bruker Model Fourier300 - Ultra Shield®, with 7.05" magnet T, and dual direct detection at 300.83 MHz for proton, 75.48 MHz for carbon

Pulse Program/ width : zg 90° - P1

Sample Temperature: 298 K, regulated ± 0.1 K

Data Points (acquired): 64 K

Zero-Filling (SI or FN): to 256 K

Dummy Scans: 4

Number of Scans (NS): 32

Relaxation delay (D1): 60s

Acquisition time (AQ or AT): 4s

Spectral window: -3 to 16ppm

Transmitter Offset: 7.5ppm

Sample preparation:

Internal calibrant/ standard (St): maleic acid (Lot n° BCC6481; Expiry date: Jan 2024; Chemical shift: 6.3 ppm) or 1,2,4,5-tetrachloro-3-nitrobenzene (Lot n° BCCH8567; Expiry date: Sep 2026; Chemical Shift: 8.5ppm) purchased from Sigma Aldrich (see table)

NMR tube size: 5mm

Samples (x) and the internal calibrant (st) were weighed (see table) and dissolved in 500 µL of DMSO-d6.

Post-acquisition processing:

Zero filling: to 256K real data

Phasing: manual phasing

Baseline correction: 5 th order polynomial

Integration: all the compound/analyte signals were integrated and averaged

Table 3S. Determination of the purity of tested compounds using quantitative NMR (absolute internal calibrant method).

	Sample (x)			Internal Calibrant (st)					
Compound	m_x (mg)	MW_x (g/mol)	n	M_{st} (mg)	MW_{st} (g/mol)	Int_{st}	N_{st}	P_{st} (%)	Purity (%)
2a[a]	6.93	216.20	2	1.36	116.07	0.76	2	99.94	96.14
2b[a]	5.50	250.30	1	1.18	116.07	1.04	2	99.94	95.18
2c[a]	8.73	262.22	1	1.71	116.07	0.91	2	99.94	97.20
2d[a]	6.40	277.20	1	1.57	116.07	1.23	2	99.94	95.20
2f[b]	4.84	292.30	1	1.71	260.89	0.40	1	99.85	98.81
2g[b]	5.33	232.20	1	0.58	260.89	0.10	1	99.85	96.71
2h[b]	4.84	299.26	1	0.92	260.89	0.22	1	99.85	98.96
2i[a]	5.93	242.24	1	1.49	116.07	1.10	2	99.94	95.29
3f[a]	6.18	294.31	1	1.23	116.07	1.06	2	99.94	95.16
3g[b]	4.48	234.21	1	0.62	260.89	0.12	1	99.85	98.38

[a:] analyzed with maleic acid as internal standard; [b]: analyzed with 1,2,4,5-tetrachloro-3-nitrobenzene as internal standard

2a – qNMR

2a

Parameter	Value	
Ax	2.00	
Ast	0.76	
nst	2.00	
nx	2.00	
MWx	216.20	
MWst	116.07	
Wst	1.36	
Wx	6.93	
Pst	99.94	
Potency x	0.9613845	**96.14%**

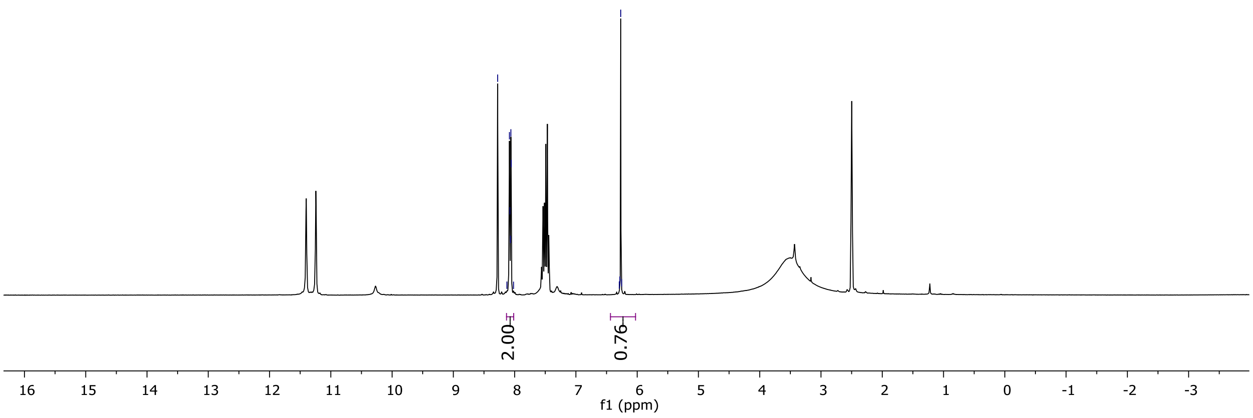

2b – qNMR

**2b**		
Parameter	Value	
Ax	1.00	
Ast	1.04	
nst	2.00	
nx	1.00	
MWx	260.21	
MWst	116.07	
Wst	1.18	
Wx	5.50	
Pst	99.94	
Potency x	0.951837778	95.18%

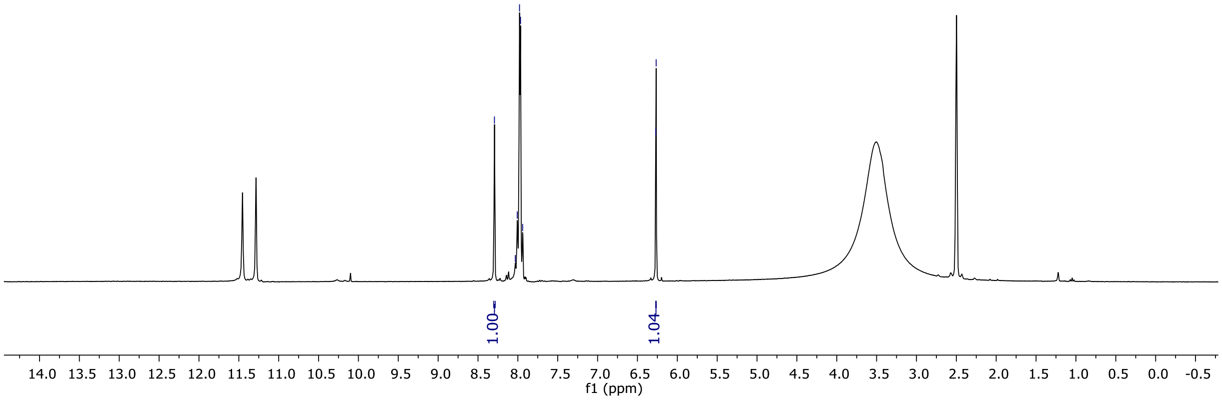

2c- qNMR

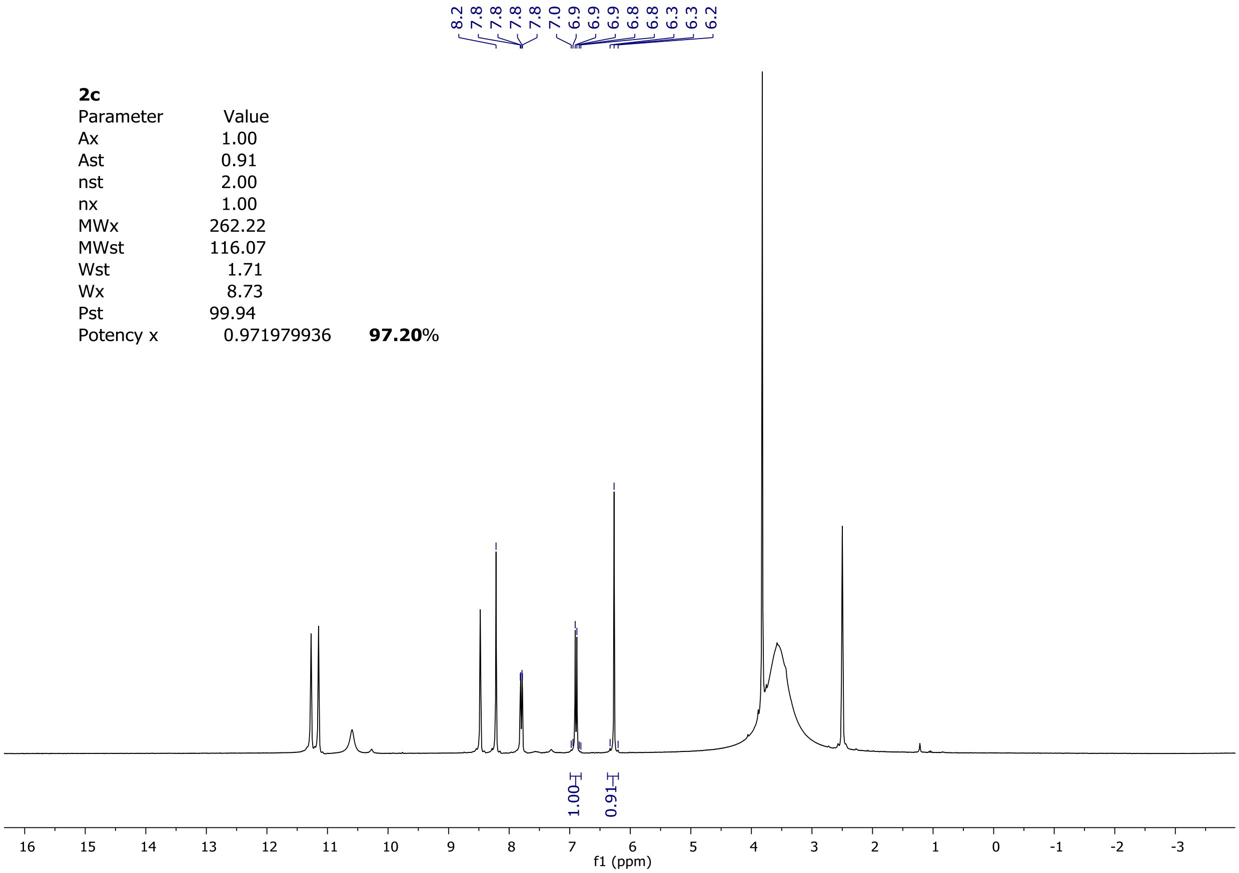

2d – qNMR

2d

Parameter	Value	
Ax	1.00	
Ast	1.23	
nst	2.00	
nx	1.00	
MWx	277.20	
MWst	116.07	
Wst	1.57	
Wx	6.40	
Pst	99.94	
Potency x	0.952044	**95.20%**

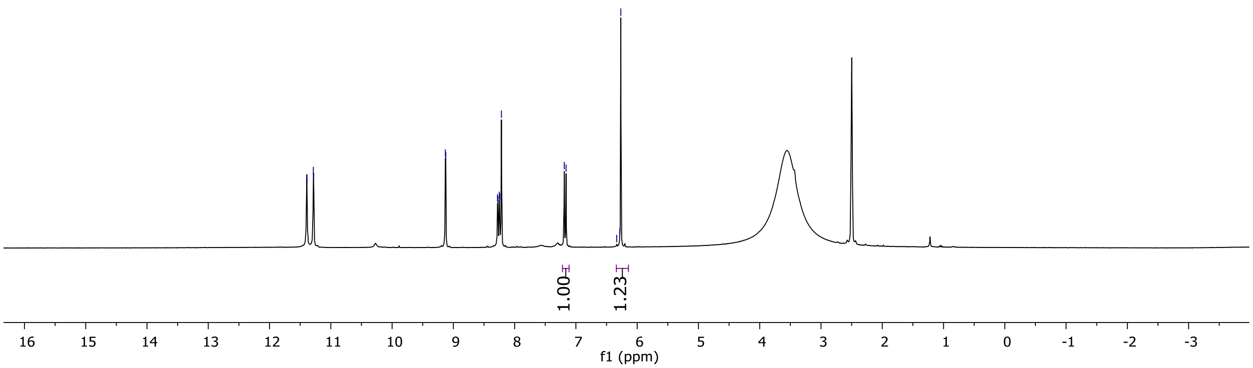

2f – qNMR

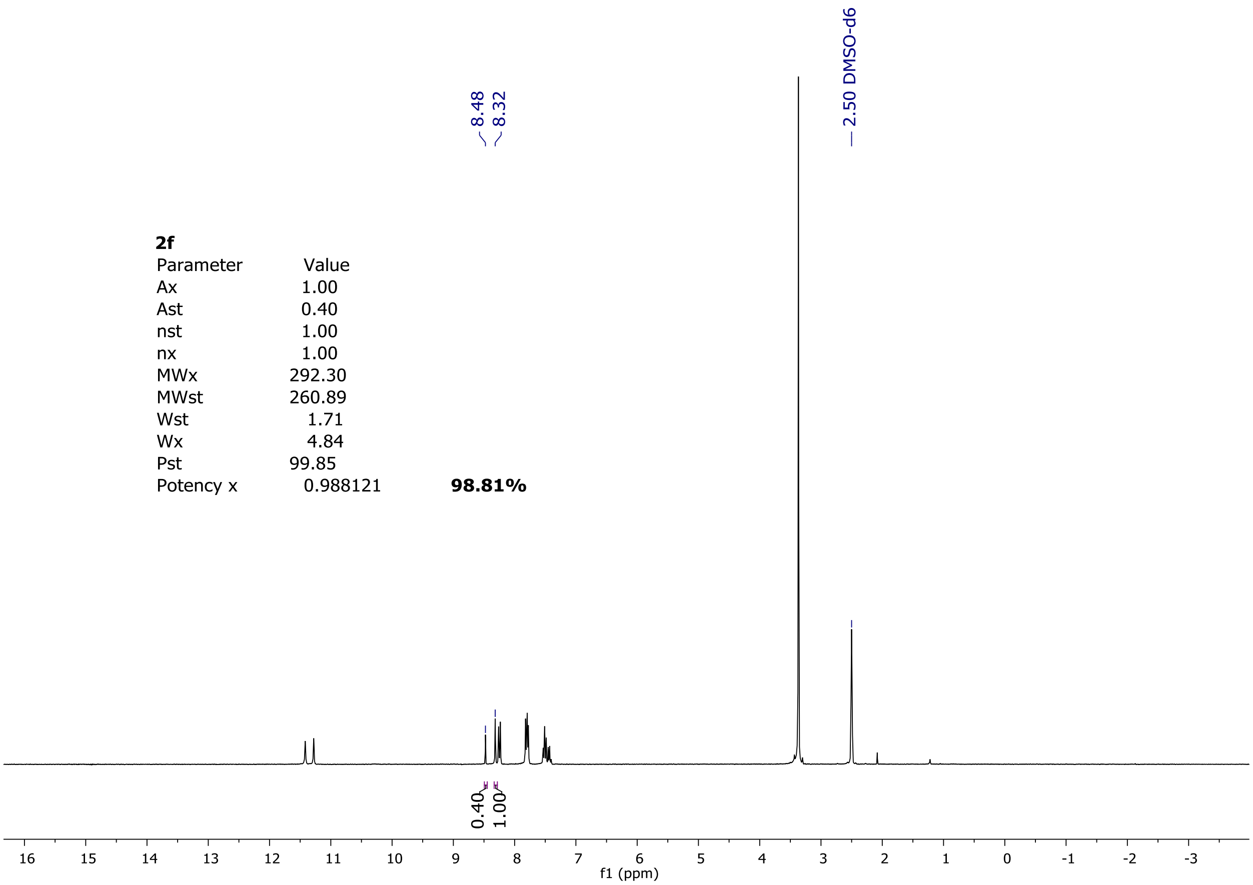
2f
Parameter Value
Ax 1.00
Ast 0.40
nst 1.00
nx 1.00
MWx 292.30
MWst 260.89
Wst 1.71
Wx 4.84
Pst 99.85
Potency x 0.988121 98.81%
8.48
8.32
2.50 DMSO-d6
0.40
1.00
16 15 14 13 12 11 10 9 8 7 6 5 4 3 2 1 0 -1 -2 -3
f1 (ppm)

2g – qNMR

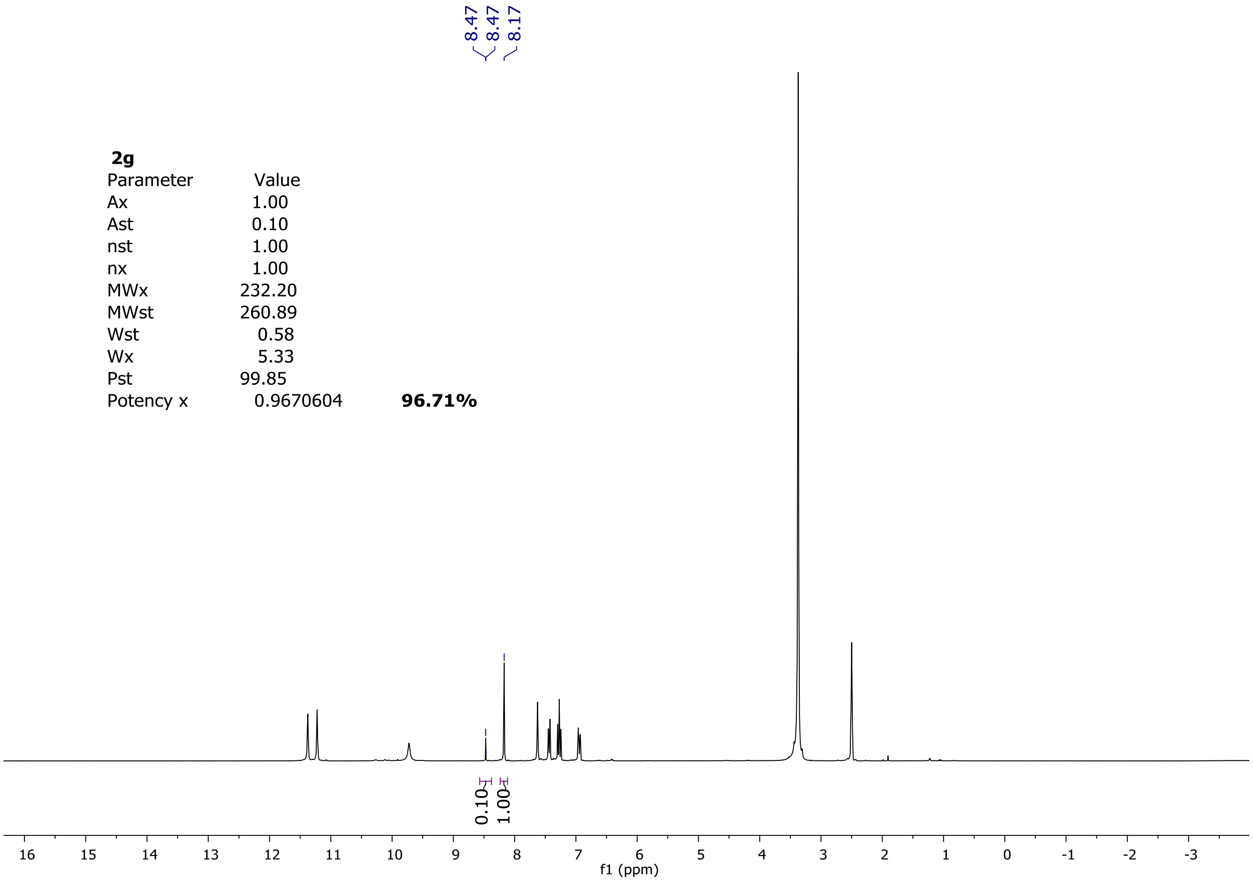

2g

Parameter	Value	
Ax	1.00	
Ast	0.10	
nst	1.00	
nx	1.00	
MWx	232.20	
MWst	260.89	
Wst	0.58	
Wx	5.33	
Pst	99.85	
Potency x	0.9670604	**96.71%**

2h – qNMR

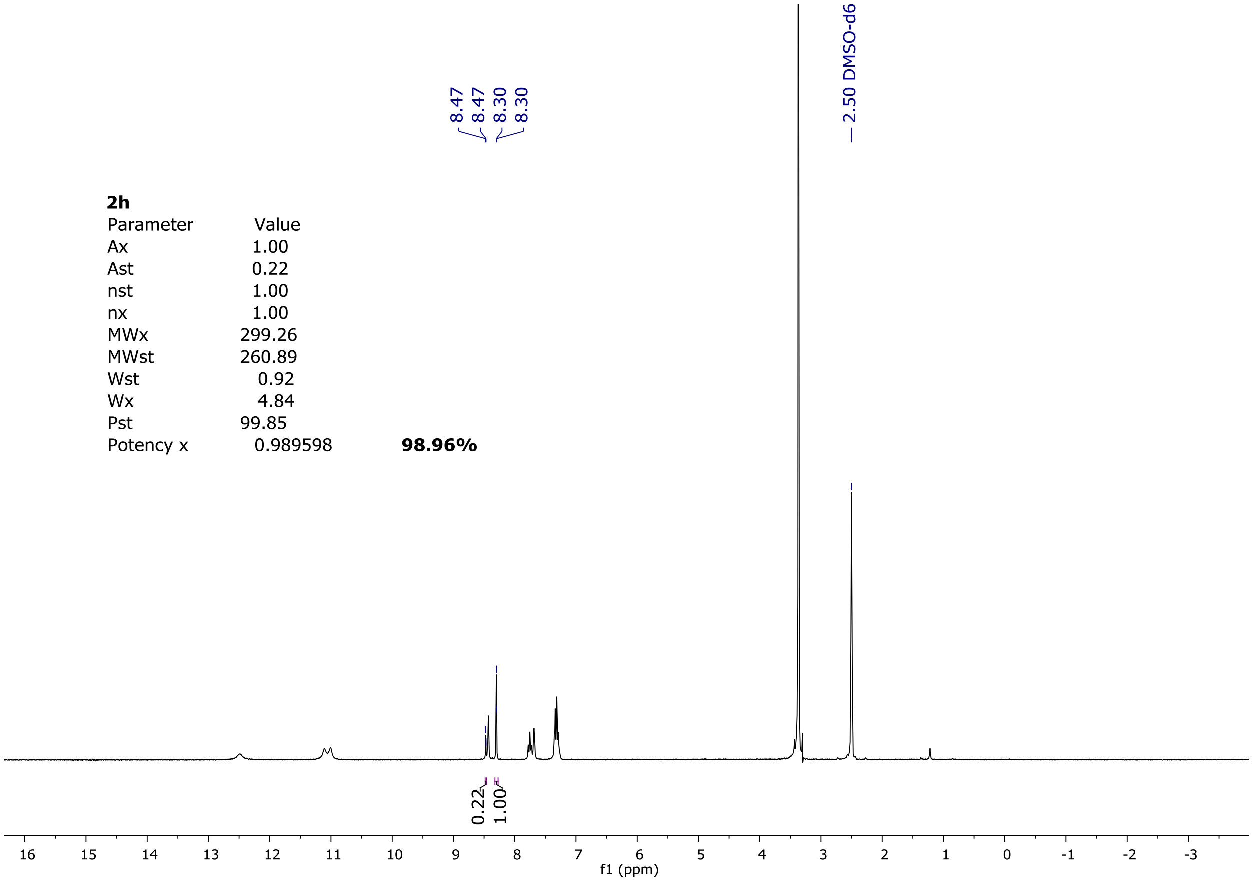
8.47
8.47
8.30
8.30
2.50 DMSO-d6
2h
Parameter Value
Ax 1.00
Ast 0.22
nst 1.00
nx 1.00
MWx 299.26
MWst 260.89
Wst 0.92
Wx 4.84
Pst 99.85
Potency x 0.989598 98.96%
0.22
1.00
16 15 14 13 12 11 10 9 8 7 6 5 4 3 2 1 0 -1 -2 -3
f1 (ppm)

2i - qNMR

2i

Parameter	Value	
Ax	1.00	
Ast	1.10	
nst	2.00	
nx	1.00	
MWx	242.23	
MWst	116.07	
Wst	1.49	
Wx	5.93	
Pst	99.94	
Potency x	0.9528709	**95.29%**

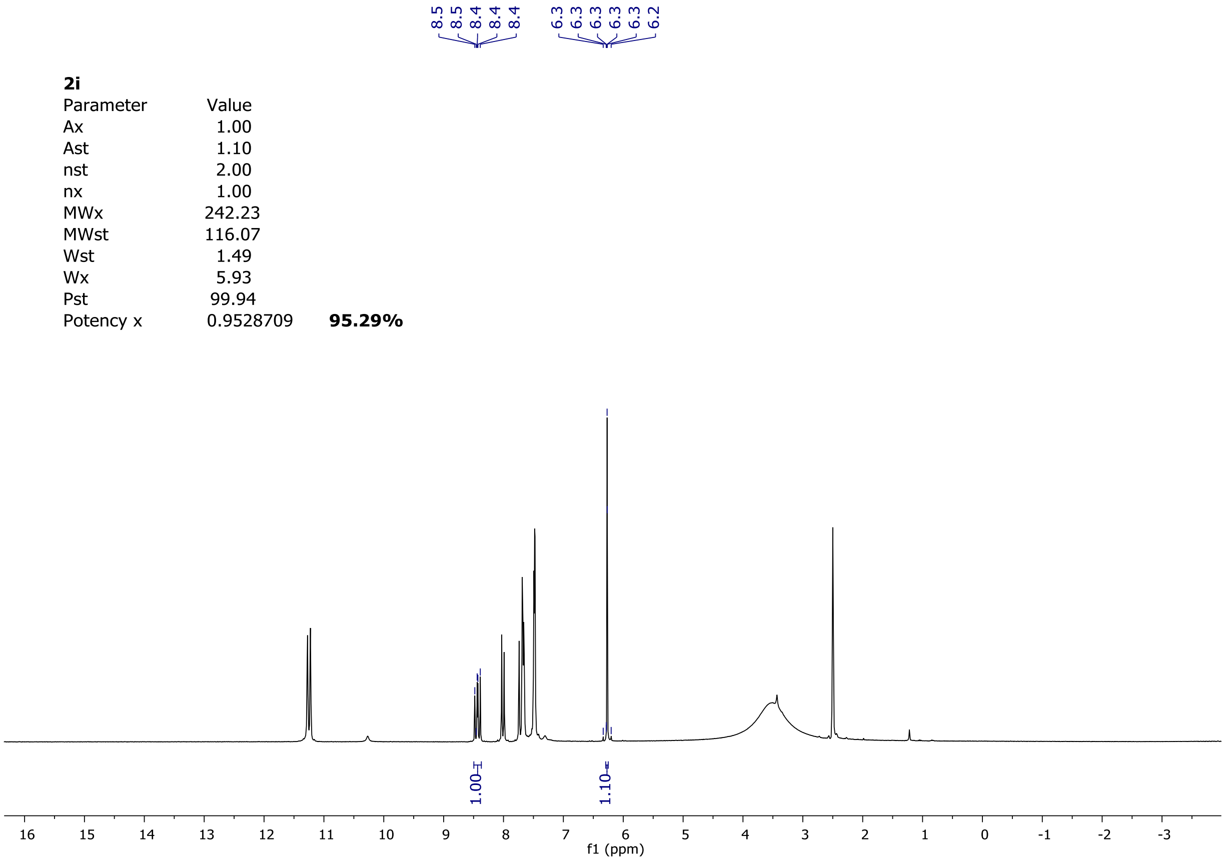

3f – qNMR

3f

Parameter	Value	
Ax	1.00	
Ast	1.06	
nst	2.00	
nx	1.00	
MWx	294.31	
MWst	116.07	
Wst	1.23	
Wx	6.18	
Pst	99.94	
Potency x	0.951623473	**95.16%**

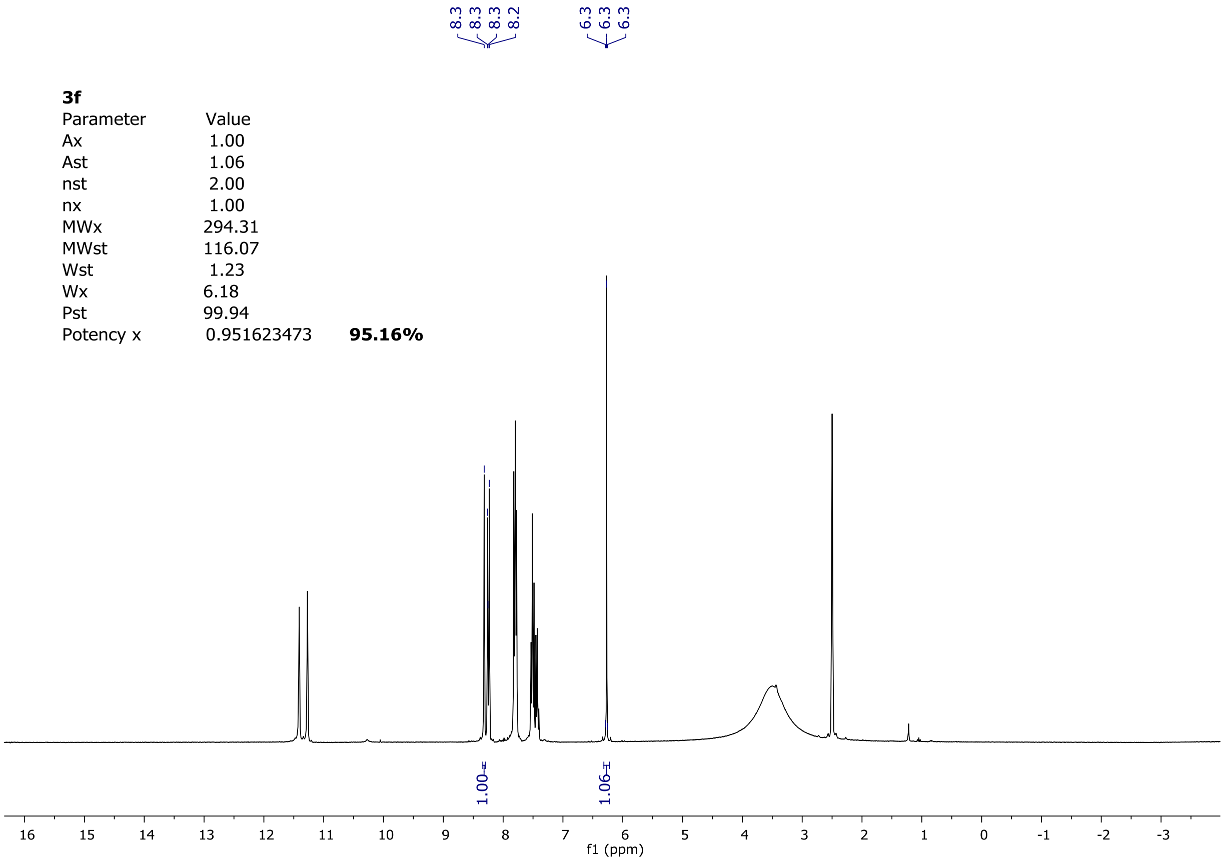

3g – qNMR

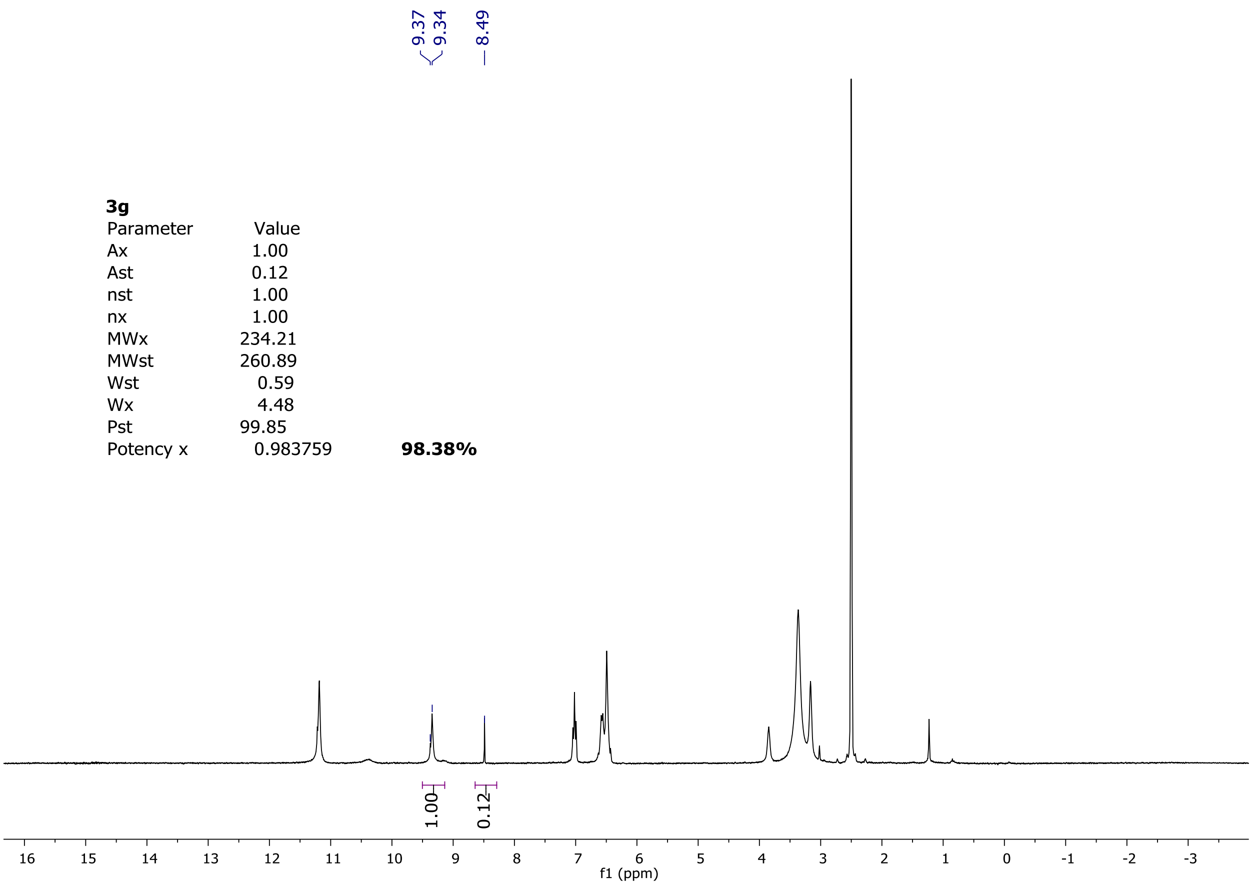
9.37
9.34
8.49
3g
Parameter Value
Ax 1.00
Ast 0.12
nst 1.00
nx 1.00
MWx 234.21
MWst 260.89
Wst 0.59
Wx 4.48
Pst 99.85
Potency x 0.983759 98.38%
1.00
0.12
16 15 14 13 12 11 10 9 8 7 6 5 4 3 2 1 0 -1 -2 -3
f1 (ppm)

HPLC data of compound 2i

Method development for the characterization of compound 2i was carried out with an HPLC system. The detector was set at 254 nm. The system was controlled, and data analyses were performed using the LC solutions software. The solvents were filtered through a 0.45 µm Merck-Millipore filter before use and degassed in an ultrasonic bath.
Volumes of 50 µL (analytical) were injected. Quantification was carried out at 254 nm, and the chromatographic run time varied according to each sample. Compound 2i is judged to be more than 95% pure by HPLC (UV at 254 nm).

Purity (HPLC):
Equipment: UFLC Shimadzu Prominence equipment; UV detector: SPD-M20A; auto sampler: SIL-20A; pump: LC-20AD.

solvent: A: water with 0.05 % (v/v) trifluoroacetic acid

B: CH3CN

guard column: Shim-pack GIST C18 Guard Column
column: Shim-pack GIST C18 Capillary Column – 250 mm × 4.6 mm × 5µm, Endcapped, pore size 10 nm or 100 Å; surface area 350 m²/g,
pH range: 1.0 to 10.0, carbon loading: 10%.

temperature: 35 °C
gradient:

**time [min]**	**solvent A [%]**	**solvent B [%]**
6.0	95.0	5.0
15.0	0.0	100
19.0	95.0	5.0
20.0	95.0	5.0
40.0	90	10

flow rate: 0.5 mL/min
injection: 50.0 uL
detection: wavelength: 254 nm
stop time: 20 min

calculation: integration: manual
calculation method: area %

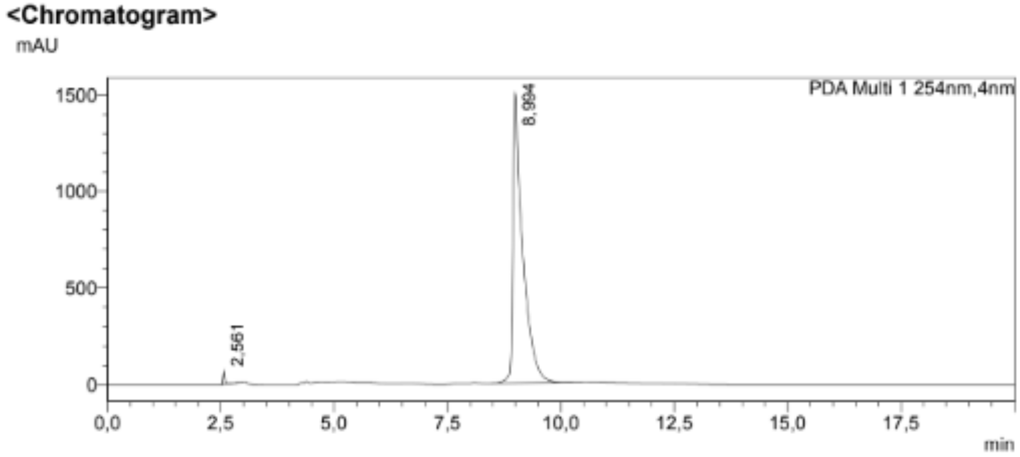

Peak Table

PDA Ch1 254nm

Peak#	Ret. Time	Area	Height	Area%
1	2,561	158530	67090	0,707
2	8,994	22260899	1497068	99,293
Total		22419429	1564157	100,000

Figure 5S: The relative energies of the frontier molecular orbitals of barbituric derivatives: (a) 2d and (b) 2g. The HOMO orbitals are highlighted in blue and red, while the LUMO orbitals are shown in green and cyan.

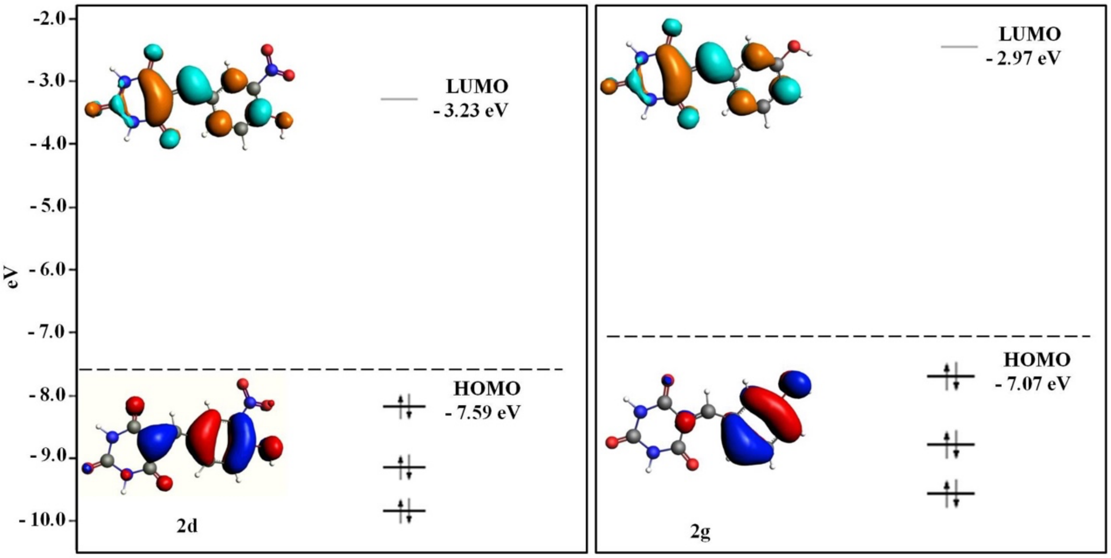

Density functional theory (DFT) calculations were performed using the Amsterdam Density Functional (ADF) software package. Initially, the compounds were drawn using AMSjobs, followed by a preliminary geometry optimization in AMSinput. Subsequently, the ground-state geometry of representative barbituric derivatives was optimized at the B3LYP/TZ2P level of theory. The HOMO and LUMO orbitals were analyzed, focusing on the occupied and the lowest unoccupied molecular orbitals (MOs), which are critical for understanding the intrinsic reactivity of the compounds.

References

(1) Corpet, F. Multiple Sequence Alignment with Hierarchical Clustering. *Nucleic Acids Res* **1988**, *16* (22), 10881. https://doi.org/10.1093/NAR/16.22.10881.

(2) Gouet, P.; Courcelle, E.; Stuart, D. I.; Métoz, F. ESPript: Analysis of Multiple Sequence Alignments in PostScript. *Bioinformatics* **1999**, *15* (4), 305–308. https://doi.org/10.1093/BIOINFORMATICS/15.4.305.
